# Reaction of FcC≡CC(O)R (Fc = ferrocenyl) with Ru_3_(CO)_12_ leading to unexpected nitro-group reduced ruthenoles and 1,2-CO-inserted triruthenium clusters†

**DOI:** 10.1039/c8ra04548h

**Published:** 2018-07-16

**Authors:** Lei Xu, Liping Jiang, Shasha Li, Guofang Zhang, Weiqiang Zhang, Ziwei Gao

**Affiliations:** Key Laboratory of Applied Surface and Colloid Chemistry, MOE/School of Chemistry and Chemical Engineering, Shaanxi Normal University Xi'an 710062 China gfzhang@snnu.edu.cn

## Abstract

The reaction of Ru_3_(CO)_12_ with ferrocene-containing alkynyl ketones FcC≡CC(O)R (Fc = ferrocenyl; R = Ph (1); 2-thienyl (2); 4-CH_3_O–Ph (3); 4-NH_2_–Ph (4); 4-NO_2_–Ph (5); ferrocenyl (6)) proceeds in toluene with the formation of triruthenium clusters (1a–6a), ruthenoles (1b–5b, 5c and 1d–5d) and unexpected 1,2-CO-inserted triruthenium clusters (1c–4c). 1a–6a were isolated from the reaction of Ru_3_(CO)_12_ with one equivalent of 1–6, respectively. Ruthenoles 1b–5b, 5c and 1d–5d were collected by adding 1–5 to the corresponding 1a–5a in a molar ratio of 1 : 1, respectively. Unexpectedly, the nitro group in one of the two phenyl rings in both 5c and 5d molecules was reduced to an amino group, while their ruthenole skeletons are retained. When 1–4 were added to the corresponding 1a–4a in a molar ratio of 1 : 1, respectively, the unusual triruthenium clusters (1c–4c) were isolated, involving 1,2-insertion of a terminal coordinated carbonyl between two C≡C units of the ynone molecules. No reaction between 6a and 6 was observed. And the familiar cyclotrimerization products were not found. All new compounds were characterized by NMR, FT-IR, and MS-ESI and most of them were structurally confirmed by single crystal X-ray diffraction. The results suggested that the ferrocenyl groups in the 1,3-ynones exhibit strong electron and steric effects on the reaction process and product distribution during their reactions with Ru_3_(CO)_12_.

## Abstract 2

Unexpected nitro-group reduced ruthenoles and 1,2-CO-inserted π-coordinated triruthenium clusters were isolated during the reactions of Ru_3_(CO)_12_ with ferrocene-containing 1,3-ynones.

## Graphical abstract

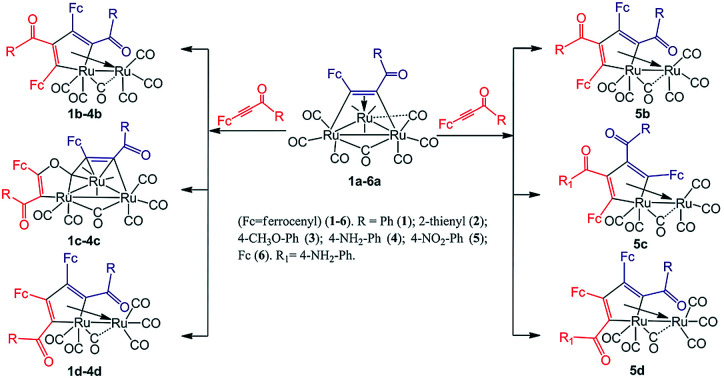

## Introduction

Ru_3_(CO)_12_ as a potent catalyst precursor has attracted great interest of researchers due to its unique activity in homogeneous catalytic reactions.^1^ It was usually used for activation and conversion of chemical bonds for the construction of diverse C–X (X = C, N, O, Si, *etc.*) bonds.^2^ To understand the related activated mechanisms, reactions of Ru_3_(CO)_12_ with NHCs,^3^ arenes,^4^ alkenes^5^ and alkynes^6^ were extensively investigated. In recent years, some carbonyl ruthenium compounds formed *via* Ru_3_(CO)_12_ and unsaturated hydrocarbons have been used in catalytic reactions.^7^ These reactions provide more possibilities for Ru_3_(CO)_12_ to become a potential catalyst for the conversion of alkyne compounds.

Ruthenium clusters containing alkyne-derived ligands have been studied for many years and a variety of coordination modes were reported.^8^ The coordination patterns are usually relevant to many catalytic processes involving polynuclear species and unsaturated organic molecules.^9^ Previously P. J. Low studied the reactions of Ru_3_(CO)_12_ with 1,6-bis(trimethylsilyl)-hexa-1,3,5-triyne and separated a series of Ru_2_–Ru_4_ clusters.^10^ In the meantime, S. W. Lau synthesized ruthenium diyne clusters with diverse Ru-diyne coordination modes *via* reaction of Ru_3_(CO)_12_ with 1,4-bis(1-hydroxycyclopentyl)-1,3-butadiyne.^11^ Then R. Rosseto systematically investigated the reaction of some asymmetrical alkynes with Ru_3_(CO)_12_ and revealed the electron effects of the groups in the aromatic ring(s).^12^ M. Li reported the trinuclear complexes (3,4-R_2_C_5_H_2_)_2_(μ_3_-C_4_Ph_2_) Ru_3_(CO)_6_(μ-CO)_2_ (R = Me, Ph) and the dinuclear complex (3,4-Ph_2_C_5_H_2_)_2_(μ-C_4_Ph_2_)Ru_2_(CO)_5_(μ-CO) by reaction of Ru_3_(CO)_12_ with {η^5^-[1,2-R_2_-4-(PhC≡C)C_5_H_2_]}_2_ZrCl_2_ (R = Me, Ph), *via* the unexpected cleavage of the two Cp–Zr bonds.^13^ Recently, P. Mathur reported a series of [Ru(CO)_3_(η^4^-ruthenole)] derivatives during the reaction of Ru_3_(CO)_12_ with FcC_2_C_2_Ph (Fc = ferrocenyl).^14^ Therefore, the reactions of Ru_3_(CO)_12_ with different acetylenes can afford a variety of unexpected products,^10–15^ which motivates us to explore the activated mechanisms *via* experienced sophisticated transformations of alkynes.

Alkynyl ketones as important functional alkynes, are often employed as key templates in modern chemical synthesis.^16^ For understanding reactive activity of 1,3-ynones with Ru_3_(CO)_12_, reactions of a few 1,3-diphenylprop-2-yn-1-one derivatives with Ru_3_(CO)_12_ have been systematically investigated in our recent studies, and a series of Ru_2_–Ru_4_ clusters were isolated.^17^ After a detailed analysis of the results, we believed that, during the reaction of a 1,3-ynone with Ru_3_(CO)_12_, not only the group at its carbonyl side exerts effects on its reactivity and transformation process, but the group at its C≡C side influences the reaction direction as well. As a continuing work in the chemistry of Ru_3_(CO)_12_ with alkynyl ketones, we introduced a ferrocenyl group at the C≡C side of a 1,3-diphenylprop-2-yn-1-one derivative to substitute the phenyl group, and investigated the structures of the intermediates and final products during its reactions with Ru_3_(CO)_12_.

In this paper, we examined in detail the reaction processes of Ru_3_(CO)_12_ with six FcC≡CC(O)R (Fc = ferrocenyl; R = Ph (1); 2-thienyl (2); 4-CH_3_O–Ph (3); 4-NH_2_–Ph (4); 4-NO_2_–Ph (5); ferrocenyl (6)) compounds. In the compounds 1–6, the selected groups at their carbonyl sides are phenyl (1) and 2-thienyl (2) rings with no substituents, phenyl rings with electron-donating groups (3 and 4) and electron-withdrawing group (5), and a sterically hindered ferrocenyl group (6). The coordination and couplings of the alkynyl ketones with Ru_3_(CO)_12_ formed a series of ruthenium clusters. Through a detailed examination of the reaction processes, we found that the ferrocenyl groups in 1–6 play important roles in the reaction process and formation of the products. Compared with the studied 1,3-diphenylprop-2-yn-1-one derivatives,^17^ the reactions of the ferrocene-containing 1,3-ynones 1–6 with Ru_3_(CO)_12_ afforded unexpected 1,2-CO-inserted π-coordinated triruthenium clusters (1c–4c), the head-to head ruthenoles (5c and 5d) with reduction of half of the nitro groups into amino groups. The common cyclotrimerization products were not isolated, however.

## Results and discussion

### Syntheses and characterization

**Scheme 1:**
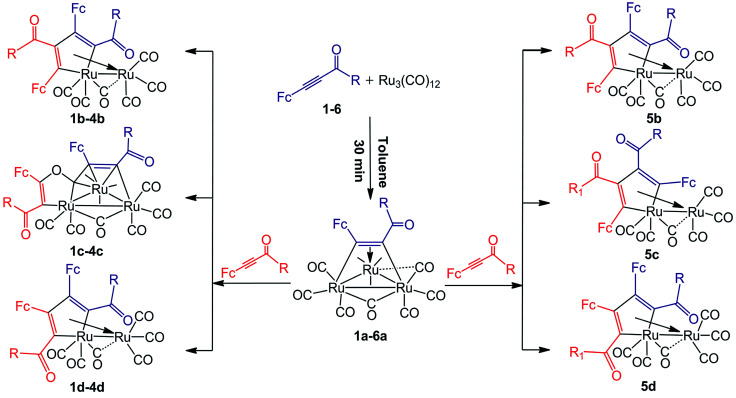
The product distribution of Ru_3_(CO)_12_ with FcC≡CC(O)R (Fc = ferrocenyl) (1–6). R = Ph (1); 2-thienyl (2); 4-CH_3_O–Ph (3); 4-NH_2_–Ph (4); 4-NO_2_–Ph (5); Fc (6).

The thermal reactions of 1,3-ynones (1–6) with Ru_3_(CO)_12_ were carried out in toluene at 90 °C under nitrogen atmosphere. The reaction courses were monitored by TLC technique. After slow cooling, the unreacted Ru_3_(CO)_12_ was filtered and recovered, the solvents were removed and the residues were chromatographed on silica gel with dichloromethane and it was found that a mixture of ruthenium clusters was obtained. Although the yield of each product is low, the yield of the mixture in each reaction is not very low. If the recovered Ru_3_(CO)_12_ was taken into account, the recovery of the ruthenium in each reaction is substantially high. According to the experimental results, the product distribution was illustrated in Scheme 1.

Formation of the clusters Ru_3_(CO)_9_(μ_2_-CO)(μ_3_-η^1^:η^2^:η^1^-triruthenium) derivatives (1a–6a) were observed in the reactions of 1–6 with Ru_3_(CO)_12_ in toluene at 90 °C for 30 min. These clusters were formed by binding of the C≡C bonds of the 1,3-ynones with Ru metal skeletons. This reaction course was similar to that of Ru_3_(CO)_12_ with a 1,3-diphenylprop-2-yn-1-one derivative.^17^ Since the molecular structures of 1a–6a are similar, 4a was taken as an example, the FT-IR absorption in the range of 2011–2098 cm^−1^ and 1878 cm^−1^ were assigned to its terminal and bridging CO groups, respectively. The chemical shift of the C≡C bond moved downwards to 152.51 ppm, confirming a strong interaction between the C≡C bond and the three Ru atoms. Since the terminal and bridging CO groups are fluxional in solution, the carbonyl carbon atoms cannot be distinguished by ^13^C{1H} NMR spectroscopy.

**Fig. 1:**
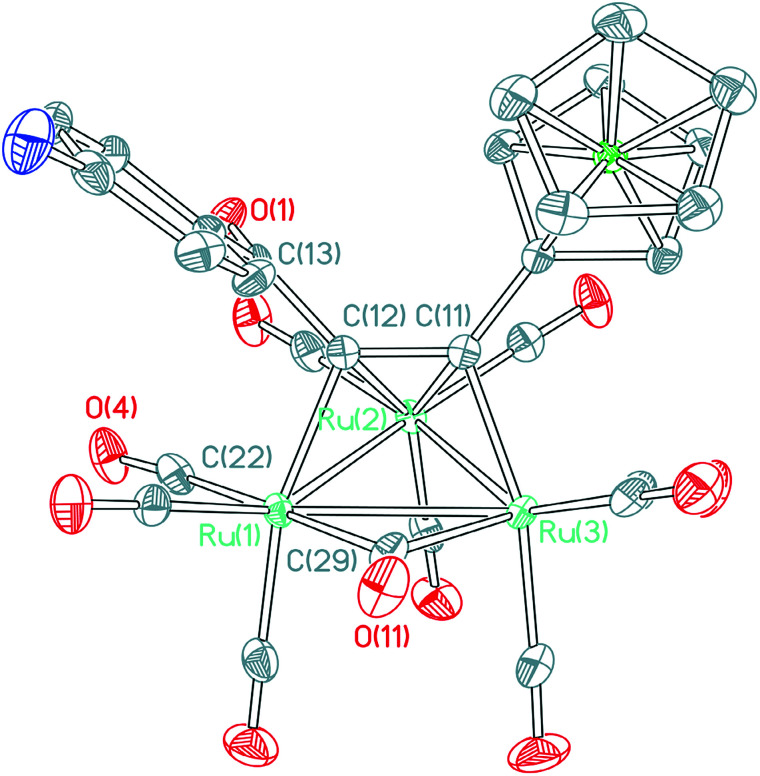
ORTEP view of cluster 4a showing 50% ellipsoids. Selected bond lengths (Å) and bond angles (°): Ru_1_–Ru_2_ = 2.7256(9); Ru_2_–Ru_3_ = 2.7191(9); Ru_1_–Ru_3_ = 2.8130(9); Ru_1_–C_22_ = 1.9143(36); Ru_1_–C_29_ = 2.1337(36); Ru_1_–C_12_ = 2.1235(34); Ru_2_–C_11_ = 2.2893(35); Ru_2_–C_12_ = 2.2415(32); Ru_2_–C_22_ = 2.9786(41); Ru_3_–C_11_ = 2.1147(36); Ru_3_–C_29_ = 2.1612(43); C_11_–C_12_ = 1.3947(46); C_12_–C_13_ = 1.4987(50); C_13_–O_1_ = 1.2258(44); C_22_–O_4_ = 1.1356(43); C_29_–O_11_ = 1.1540(5); Ru_1_–C_22_–Ru_2_ = 63.391(11); Ru_1_–C_29_–Ru_3_ = 81.830(13).

The structure of 4a (Fig. 1) consists of a triangular arrangement of the three ruthenium atoms, in which the Ru–Ru bond distances are in the range of 2.7191(9)–2.8130(7) Å. The coordination of the alkynyl ketone with Ru_2_ is in a η^2^ mode, but with Ru_1_ and Ru_3_ is in a η^1^ mode. The distances of Ru_1_–C_22_ and Ru_2_–C_22_ are 1.9143(36) and 2.9786(41) Å, respectively, indicative of the existence of a semi-bridging carbonyl group between Ru_1_ and Ru_2_ atoms. The C_11_–C_12_ bond length is 1.3947(46) Å, which is between the typical C–C single bond and double bond and is elongated significantly.^18^ The angle of Ru_1_–C_22_–O_4_ is 172.534(310)°.

**Fig. 2:**
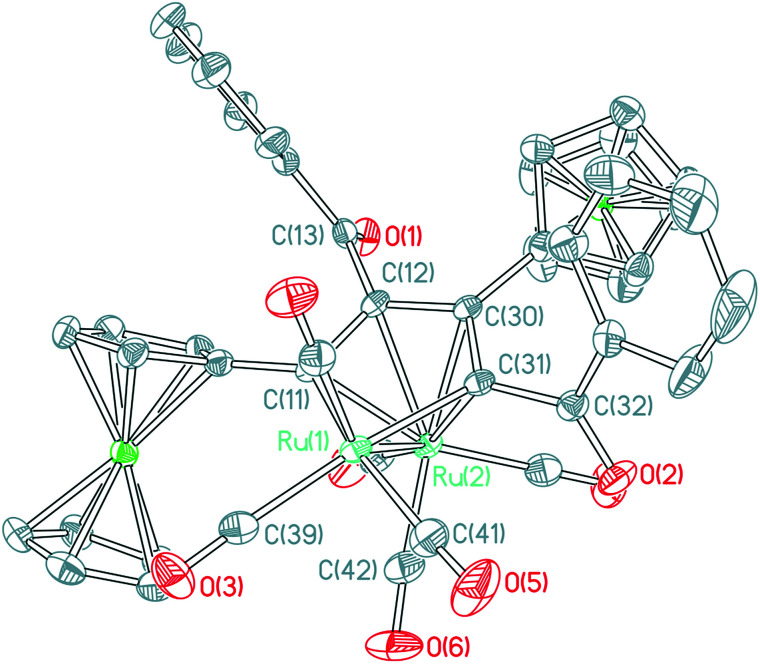
ORTEP view of cluster 1b showing 50% ellipsoids. Selected bond lengths (Å) and bond angles (°): Ru_1_–Ru_2_ = 2.7543(6); Ru_1_–C_11_ = 2.0920(28); Ru_1_–C_31_ = 2.0851(29); Ru_1_–C_39_ = 1.9625(37); Ru_1_–C_42_ = 2.7660(3); Ru_2_–C_11_ = 2.2598(21); Ru_2_–C_12_ = 2.2809(24); Ru_2_–C_31_ = 2.2066(24); Ru_2_–C_30_ = 2.2917(27); Ru_2_–C_42_ = 1.8989(45); C_11_–C_12_ = 1.4394(43); C_12_–C_30_ = 1.4439(39); C_30_–C_31_ = 1.4380(41); C_31_–C_32_ = 1.4877(39); C_32_–O_2_ = 1.2215(30); C_12_–C_13_ = 1.5148(44); C_13_–O_1_ = 1.2166(39); Ru_1_–C_42_–Ru_2_ = 69.550(12); Ru_2_–C_42_–O_6_ = 168.948(34).

By increasing the reaction time of 1,3-ynones 1–5 with Ru_3_(CO)_12_ to 2 h, three types of the common Ru(CO)_3_(η^4^-ruthenole) derivatives 1b–5b, 5c, 1d–5d and unusual 1,2-CO-inserted π-coordinated triruthenium clusters 1c–4c were isolated. The structural characterizations showed that all ruthenoles each contains a metallacyclopentadienyl framework,^19^ similar in structure to the corresponding ruthenoles we reported previously,^17^ in which two 1,3-ynone molecules couple by the C≡C units in modes head-to-tail coupling (1b–5b), head-to-head coupling (5c) and tail-to-tail coupling (1d–5d).^17^ Accordingly, we choose 1b (Fig. 2) from 1b–5b as an example to describe the molecular structures of these clusters. The structure of 1b consists of five terminal and one semi-bridging carbonyls. The distance of the Ru–Ru bond is 2.7543(6) Å. The lengths of the C–C bonds [1.4380(41)–1.4439(39) Å] in the metallacyclopentadiene (Ru_1_C_11_C_12_C_30_C_31_) reflect the interactions between the Ru_2_ atom and the 1,3-ynone, and most of the Ru–C bond lengths related with the metallacyclopentadiene fall into two distinct ranges, 2.0851(29)–2.0920(28) Å and 2.2066(24)–2.2917(27) Å. In addition, the dihedral angles between Ru_1_–Ru_2_–C_42_plane__ and Ru_1_–Ru_2_–C_39_plane__, Ru_1_–Ru_2_–C_41_plane__ are 58.4(2)° and 41.0(2)°, respectively.

Upon comparison the FT-IR, NMR and ESI-MS data of 5c with those of the characterized ruthenoles, we proposed that 5c has a similar structure to the ruthenoles with the head-to-head coupling mode of two 1,3-ynone molecules in our previous studies,^17^ with the nitro group in one phenyl ring being reduced to an amino group. Since no single crystals suitable for single-crystal X-ray diffraction were grown for 5c, DFT calculations were carried out to confirm the structure of 5c. The optimized structure of 5c and its calculated IR and NMR spectra are shown in Fig. S6,† NMR and FT-IR spectra in the ESI,† respectively. They showed that the calculated NMR and IR spectra of 5c were consistent with its determined NMR and IR spectra, confirming the structure of 5c.

**Fig. 3:**
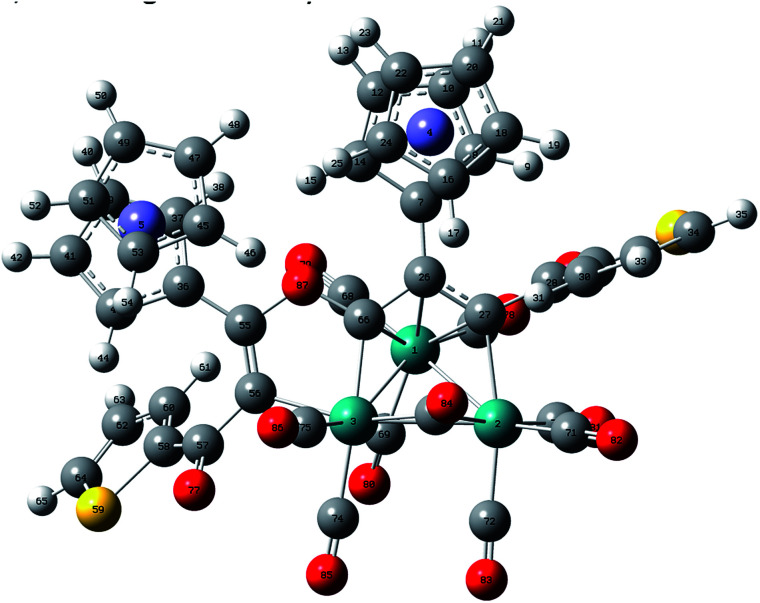
DFT-optimized structure of 2c at the level of B3LYP/LanL2DZ/6-31G. Selected bond lengths (Å) and bond angles (°): Ru_1_–Ru_2_ = 2.8093, Ru_2_–Ru_3_ = 2.9157, Ru_1_–Ru_3_ = 2.9325, Ru_1_–C_26_ = 2.4356, Ru_1_–C_66_ = 2.3153, Ru_1_–C_27_ = 2.2934, Ru_2_–C_73_ = 2.1992, Ru_3_–C_56_ = 2.1019, Ru_3_–C_73_ = 2.0764, Ru_3_–C_66_ = 2.0405, C_26_–C_27_ = 1.4355, C_26_–C_66_ = 1.4693, C_66_–O_87_ = 1.4052, C_55_–O_87_ = 1.4032, C_55_–C_56_ = 1.3601, C_56_–C_57_ = 1.4786, C_57_–O_77_ = 1.2621, C_27_–C_28_ = 1.4937, C_28_–O_76_ = 1.2599, Ru_2_–C_73_–Ru_3_ = 85.9399, Ru_3_–C_66_–O_87_ = 113.3175, Ru_3_–C_66_–C_26_ = 131.1049.

Unexpectedly, the characterization results of 1c–4c are completely different from those of the usual ruthenole such as 5c. Fortunately, crystal structure of 2c (ORTEP view of 2c is displayed in Fig. S4†) was established, although the w*R*_2_ in the crystal data of 2c was larger than 0.15. Then we employed DFT calculations to verify its structure. The calculated molecular structure of 2c (shown in Fig. 3) is identical to the established one, indicating that the crystal structure of 2c is correct.

Although a similar skeleton to the structure of 2c has been reported in the reaction of Os_3_(CO)_10_(MeCN)_2_ with terminal acetylene ligands,^20^ this type of structure formed by ruthenium atoms has never been reported until now. The structure of 2c consists of a triangular arrangement of the ruthenium atoms with the Ru–Ru bond distances in the range of 2.7681(17)–2.8236(16) Å. Two 1,3-ynone molecules are coupled by the 1,2-insertion of a terminal coordinated CO molecule in two carbon–carbon triple bonds. The dimerized 1,3-ynone ligands are bound to the triruthenium core by three σ-bonds Ru_1_–C_11_, Ru_1_–C_12_ and Ru_1_–C_35_ and its allyl moiety C_11_C_12_C_35_ is π-coordinated by the Ru_1_ atom. Two five-membered cycles Ru_3_C_35_O_3_C_28_C_29_ and Ru_2_C_12_C_11_C_35_Ru_3_ are fused *via* the Ru_3_–C_35_ bond and do not display considerable deviations from planarity (maximum deviations from their mean planes are 0.2187(16) and 0.1013(16) Å, respectively), the dihedral angle formed by their planes is equal to 24.415(34)°. The angles of Ru_3_–C_35_–O_3_, C_11_–C_35_–O_3_ and Ru_3_–C_35_–C_11_ are 115.182(10)°, 116.011(13)° and 128.792(11)°, respectively.

**Fig. 4:**
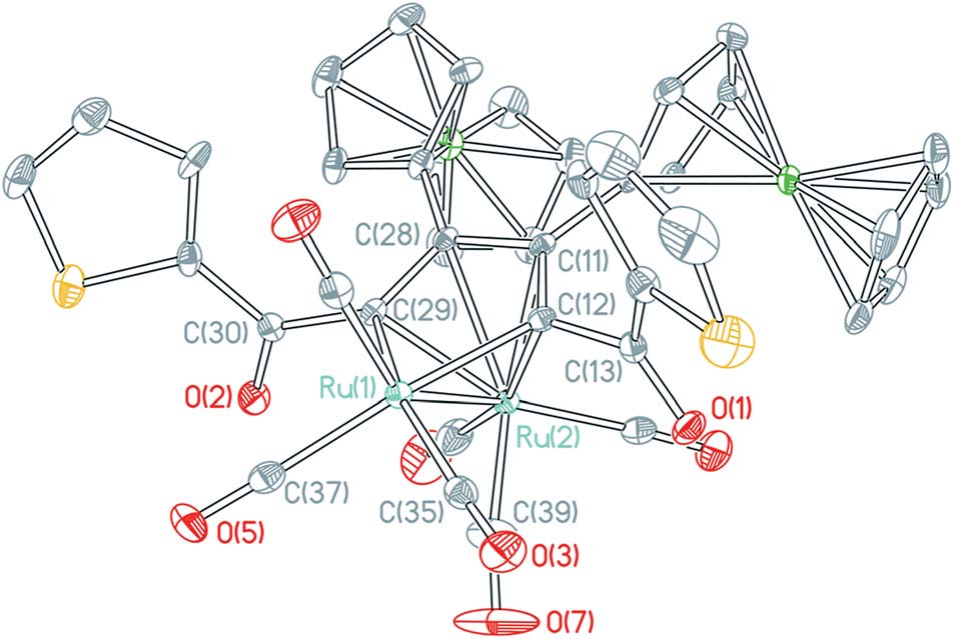
ORTEP view of clusters 2d showing 50% ellipsoids. Selected bond lengths (Å) and bond angles (°): Ru_1_–Ru_2_ = 2.7285(3); Ru_1_–C_29_ = 2.0860(28); Ru_1_–C_12_ = 2.0873(25); Ru_1_–C_39_ = 2.7101(30); Ru_2_–C_29_ = 2.2053(25); Ru_2_–C_28_ = 2.3248(24); Ru_2_–C_11_ = 2.2914(21); Ru_2_–C_12_ = 2.2319(22); Ru_2_–C_39_ = 1.9039(34); C_28_–C_29_ = 1.4275(36); C_28_–C_11_ = 1.4661(39); C_11_–C_12_ = 1.4210(37); C_12_–C_13_ = 1.4877(40); C_29_–C_30_ = 1.4862(39); C_13_–O_1_ = 1.2272(34); C_30_–O_2_ = 1.2328(27); Ru_2_–C_39_–O_7_ = 167.362(29); Ru_1_–C_39_–Ru_2_ = 70.028(10).

**Fig. 5:**
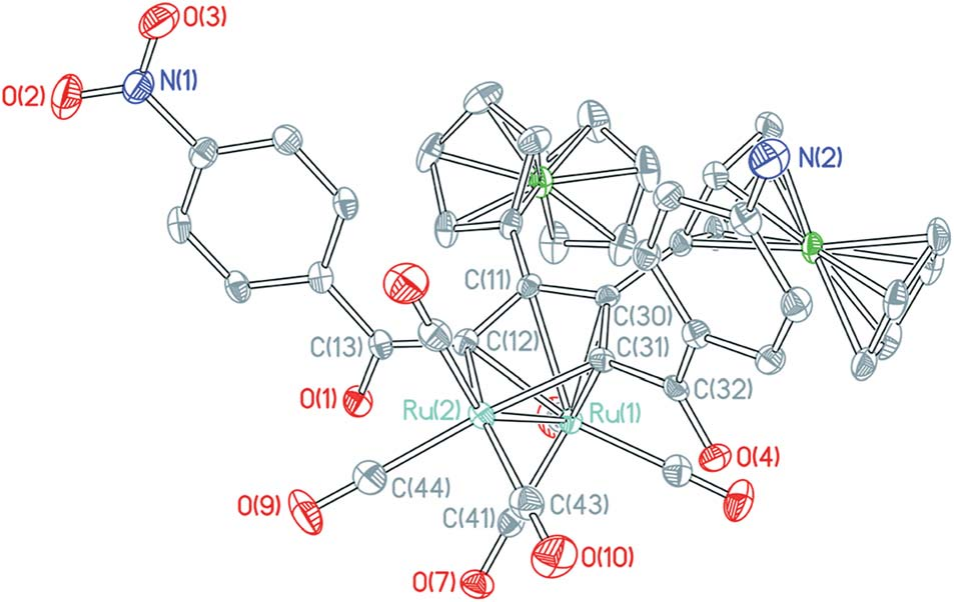
ORTEP view of clusters 5d·CH_2_Cl_2_ showing 50% ellipsoids (solvent molecules have been omitted for clarity). Selected bond lengths (Å) and bond angles (°): Ru_1_– Ru_2_ = 2.7504(3); Ru_1_–C_41_ = 1.9080(24); Ru_2_–C_41_ = 2.8071(24); Ru_2_–C_12_ = 2.0928(22); Ru_2_–C_31_ = 2.0811(23); C_11_–C_12_ = 1.4275(32); C_11_–C_30_ = 1.4611(30); C_30_–C_31_ = 1.4274(31); C_31_–C_32_ = 1.4954(30); C_32_–O_4_ = 1.2316(28); C_12_–C_13_ = 1.4829(31); C_13_–O_1_ = 1.2280(28); Ru_1_–C_41_–O_7_ = 173.338(21); Ru_1_–C_41_–Ru_2_ = 68.331(62).

The molecular structures of ruthenoles 1d–5d (tail-to-tail coupling mode) are similar, therefore the structures of both 2d and 5d·CH_2_Cl_2_ are taken as examples and their ORTEP views are shown in Fig. 4 and 5. The distances of the Ru–Ru bonds in 2d and 5d·CH_2_Cl_2_ are 2.7285(3) and 2.7504(3) Å, respectively, and the differences between most of the Ru–C bond distances and of the C–C bond lengths bound with the Ru atoms are within a small ranges, a similar semi-bridging carbonyl group existed between two Ru atoms. In structure of 2d the angle between C_29_–C_28_–C_11_–C_12_ plane and Ru_1_–Ru_2_ is 39.145°, and the angle between C_31_–C_30_–C_11_–C_12_ plane and Ru_1_–Ru_2_ in 5d is 39.882°, which are significantly different from that in the rhodium compound (η-C_5_H_5_)_2_Rh_2_(μ-CO){μ-η^2^:η^2^-C(CF_3_)H=C(CF_3_)CMe=CH_2_} reported by R. S. Dickson.^21^ Although, the cluster 5d·CH_2_Cl_2_ has a similar skeleton structure to 1d–4d, but in the molecule 5d·CH_2_Cl_2_ a nitro group at the *para* position of a phenyl ring was reduced to an amino group, as encountered in 5c. Its NMR, FT-IR and MS spectra support the existence of the amino group.

### Transformation process from 1,3-ynones to final products

The reaction of Ru_3_(CO)_12_ with ferrocene-containing 1,3-ynones (1–6) were studied in detail. Experimental results show that the reaction processes during the formation of 1a–6a were similar to those reported in our work earlier.^17^ However, the successive reaction of 1a–6a with the corresponding 1–6 afforded some unexpected products, but gave no anticipated cyclotrimerization products. According to the explanation that an electron-withdrawing group activates an alkyne and meanwhile an electron-donating group deactivates an alkyne,^22^ the larger steric hindrance and electron-donating property of two ferrocenyl groups in 6 results in the formation of 6a as the unique product in the reaction of 6 with Ru_3_(CO)_12_. Meanwhile, no cyclotrimerization products of the 1,3-ynones were formed, due mainly to the lower activity of the alkynyl ketones in the presence of ferrocenyl groups.

Moreover, we also found that formation of the final products has been governed jointly by the electronic properties of both groups at both sides of the C≡C unit of a 1,3-ynone. For example, 5 has a strong electron-withdrawing *para*-nitrophenyl group at its carbonyl side, its reaction with 5a can afford ruthenole derivatives 5b, 5c and 5d. However, in both 5c and 5d, it is noted that one of the two nitro groups in each molecule is reduced to an amino group. A. Bassoli and A. Thurkauf reported that nitro groups of nitrobenzene derivatives can be reduced by CO to amino groups in the presence of catalytic amounts of Ru_3_(CO)_12_.^23^ M. Lauwiner also approved that electron-withdrawing and/or weak electron-donating groups on the azo bridge at *para* position of nitrobenzene derivatives is beneficial to nitro group reduction in 4-nitrophenylazobenzenes by hydrazine hydrate in the presence of iron oxide/hydroxide catalyst.^24^ Therefore, we supported that the reduction of nitro groups in the phenyl rings of 5c and 5d was accelerated by CO, with Ru_3_(CO)_12_ as catalyst, and the electron-donating ferrocenyl group. During the reaction of 1-(4-nitro-phenyl)-3-phenylprop-2-yn-1-one with Ru_3_(CO)_12_, the strong electron-donating property of the phenyl ring leads to the retaining of the nitro groups in the corresponding ruthenoles.^17*b*^

In the case of the reaction of 1a–4a with the corresponding alkynyl ketone 1–4, no common head-to-head coupled ruthenole was found, the rare 1,2-CO-inserted π-coordinated triruthenium cluster 1c–4c were formed instead. We noted that there are no electron-withdrawing groups at the carbonyl sides of these alkynyl ketones. We surmised that electron effect plays a significant role in the reaction directing, taking 2c as an example: the 2-thienyl group is an electron-donating group and thus increases the electron density of the C≡C bond and deactivates the reactivity of the C≡C bond with Ru_3_ in some degree, C_28_ of the C≡C bond does not link in this case with Ru_3_, instead bonds with O_3_ of the terminal CO coordinated with Ru_3_, and C_35_ of the CO inserts between the C_11_ and Ru_3_ bond, thus finishing the 1,2-insertion of the CO group in the two 1,3-ynone molecules, forming the C_35_C_11_C_12_ π-coordinated triruthenium cluster 2c.

## Experimental

### General procedures

All reactions and manipulations were performed under dry high-purity nitrogen using standard Schlenk techniques. Ru_3_(CO)_12_ and 1–6 were synthesized according to the literature procedures.^25^ The solvents used in the experiments were purified, dried and distilled from sodium under a nitrogen atmosphere prior to use. Preparative TLC was performed on 20 × 20 cm glass plates coated with silica gel (Merck GF254, 0.5 mm thick). FT-IR spectra were recorded on a Bruker Tensor 27 Fourier-transform spectrometer. ^1^H and ^13^C{1H} NMR spectra were performed on a Bruker Avance 400 MHz spectrometer unless indicated. ESI was recorded on a Thermo DecaMax (LC-MS) mass spectrometer with an ion-trap mass detector. While high-resolution mass spectra were recorded in ESI mode on a Waters UPLC-Q-TOF mass spectrometer.

### Synthesis

1-Phenyl-3-ferrocenyl-2-yn-1-one (1), 1-(2-thienyl)-3-ferrocenyl-2-yn-1-one (2), 1-(4-methoxy-phenyl)-3-ferrocenyl-2-yn-1-one (3), 1-(4-amino-phenyl)-3-ferrocenyl-2-yn-1-one (4), 1-(4-nitro-phenyl)-3-ferrocenyl-2-yn-1-one (5) and 1,3-ferrocenyl-2-yn-1-one (6) were used to react with Ru_3_(CO)_12_. Since the reaction processes are similar, taking reaction procedure of 1 with Ru_3_(CO)_12_ as an example. Both 1 (0.1856 g, 0.9 mmol) and Ru_3_(CO)_12_ (0.1918 g, 0.3 mmol) were added in 15 mL toluene and heated at 90 °C for 30 min, it was found that color of each reaction solution gradually changed from red-brown to black. The black solution was cooled and the unreacted orange-red Ru_3_(CO)_12_ was precipitated and recovered (0.697 g). The residue was chromatographed by 305 mm length and 32 mm internal diameter chromatographic column on silica gel with dichloromethane and petroleum ether. The main products were eluted in the sequence of 1a, 1b, 1c and 1d with the eluent being dichloromethane/petroleum ether (v/v) 1 : 10, 1 : 4, 1 : 3 and 1 : 1, respectively. And then the products were recrystallized by dichloromethane and hexane. The yields of the clusters 1a, 1b, 1c and 1d were calculated based on the added Ru_3_(CO)_12_ in the beginning of the reaction.

#### [Ru_3_(CO)_9_(μ_2_-CO){μ_3_-η^1^:η^2^:η^1^-(Ph)C(O)CC(Fc)}] (1a)

Red-brown powder. Yield: 8%. FT-IR (KBr, cm^−1^): 3095 w, 2962 w, 2926 w, 2852 w, 2099 s, 2056 vs, 2028 vs, 2002 vs, 1962 s, 1860 m. ^1^H NMR (400 MHz, CDCl_3_) *δ* 8.18–8.20 (dd, 2H, C_6_H_5_), 7.53–7.55 (m, 3H, C_6_H_5_), 3.88–4.10 (m, 9H, C_10_H_9_). ^13^C{1H} NMR (101 MHz, CDCl_3_) *δ* 195.26 (CO), 166.77, 166.69 (C≡C), 132.24, 129.62, 127.59 (C_6_H_5_), 94.43, 68.67 (C_10_H_9_). MS (*m*/*z*, ESI−) 901.792 (M−). Anal. calcd for C_29_H_14_O_11_FeRu_3_: 901.702.

#### [Ru(CO)_3_{μ_4_-η^1^:η^2^:η^1^:η^1^(PhC(O))CC(Fc)C(PhC(O))C(Fc)Ru(CO)_3_}μ-CO] (1b)

Red-brown powder. Yield: 15%. FT-IR (KBr, cm^−1^): 3092 w, 2081 vs, 2053 vs, 2010 vs, 1982 vs*.*^1^H NMR (400 MHz, CDCl_3_) *δ* 7.93–7.94 (d, 2H, C_6_H_5_), 7.77–7.78 (d, 2H, C_6_H_5_), 7.30–7.58 (m, 6H, C_6_H_5_), 3.67–4.04 (m, 18H, C_10_H_9_). ^13^C{1H} NMR (101 MHz, CDCl_3_) *δ* 197.26, 196.56, 196.06, 193.98, 193.72, 193.33 (CO), 164.65 (C≡C), 136.31, 135.58, 134.81, 133.73, 132.43, 129.58, 129.33, 128.89, 128.48, 128.03 (C_6_H_5_), 81.90, 75.61, 71.21, 70.72, 70.24, 69.72, 68.63, 68.56, 68.52, 68.42, 68.18, 67.49 (C_10_H_9_). MS (*m*/*z*, ESI−) 998.860 (M−). Anal. calcd for C_44_H_28_O_8_Fe_2_Ru_2_: 998.858.

#### [Ru_3_(CO)_9_(μ-CO){μ_2_-η^1^:η^1^-PhC(O)CC(Fc)}OC{μ_3_-η^1^:η^2^-(Fc)CCC(O)Ph}] (1c)

Black powder. Yield: 13%. FT-IR (KBr, cm^−1^): 3090 w, 2924 w, 2849 w, 2099 vs, 2060 vs, 2036 vs, 2008 vs, 1998 s, 1858 m. ^1^H NMR (400 MHz, CDCl_3_) *δ* 7.77–8.19 (m, 4H, C_6_H_5_), 7.30–7.62 (m, 6H, C_6_H_5_), 3.67–4.90 (m, 18H, C_10_H_9_). ^13^C{1H} NMR (101 MHz, CDCl_3_) *δ* 216.67, 203.50, 198.49, 197.25, 195.72, 195.05, 189.09, 184.19 (CO), 164.07 (C≡C), 137.18, 136.76, 136.55, 133.72, 133.29, 133.17, 132.94, 132.85, 132.42, 129.63, 129.58, 129.33, 128.91, 128.62, 128.55, 128.17, 128.03, 114.67 (C_6_H_5_), 82.35, 76.27, 71.88, 71.21, 70.72, 70.24, 70.15, 70.07, 70.01, 69.70, 69.44, 68.80, 68.74, 68.18, 67.85, 67.61, 67.49, 67.20 (C_10_H_9_). MS (*m*/*z*, ESI−) 1215.747 (M−). Anal. calcd for C_48_H_28_O_12_Fe_2_Ru_3_: 1215.742.

#### [Ru(CO)_3_{μ_4_-η^1^:η^2^:η^1^:η^1^(Fc)CC(PhC(O))C(PhC(O))C(Fc)Ru(CO)_3_} μ-CO] (1d)

Red-brown powder. Yield: 17%. FT-IR (KBr, cm^−1^): 3100 w, 3008 w, 2961 w, 2925 w, 2849 w, 2086 vs, 2054 vs, 2027 vs, 2011 vs, 1985 vs, 1637 m. ^1^H NMR (400 MHz, CDCl_3_) *δ* 8.01–8.03 (d, 4H, C_6_H_5_), 7.46–7.56 (m, 6H, C_6_H_5_), 4.22 (s, 2H, C_10_H_9_), 4.15 (s, 2H, C_10_H_9_), 4.02 (s, 2H, C_10_H_9_), 3.96 (s, 2H, C_10_H_9_), 3.92 (s, 10H, C_10_H_9_). ^13^C{1H} NMR (101 MHz, CDCl_3_) *δ* 195.56, 195.05, 194.83, 193.14 (CO), 168.46 (C≡C), 135.61, 132.69, 130.14, 128.91, 128.04, 124.38 (C_6_H_5_), 84.26, 77.34, 77.02, 76.70, 74.28, 71.58, 70.40, 68.73, 68.55, 67.58 (C_10_H_9_). MS (*m*/*z*, ESI−) 998.851 (M−). Anal. calcd for C_44_H_28_O_8_Fe_2_Ru_2_: 998.858.

#### [Ru_3_(CO)_9_(μ_2_-CO){μ_3_-η^1^:η^2^:η^1^-(2-C_4_H_3_SC(O))CC(Fc)}] (2a)

Red-brown powder. Yield: 9%. FT-IR (KBr, cm^−1^): 3090 w, 2923 w, 2100 s, 2056 vs, 2021 vs, 2004 vs, 1962 s, 1849 m. ^1^H NMR (400 MHz, CDCl_3_) *δ* 7.98–7.99 (t, 1H, C_4_H_3_S), 7.75–7.76 (dd, 1H, C_4_H_3_S), 7.29–7.31 (m, 1H, C_4_H_3_S), 3.97–4.23 (m, 10H, C_10_H_9_). ^13^C{1H} NMR (101 MHz, CDCl_3_) *δ* 190.30 (CO), 169.94, 164.33 (C≡C), 141.29, 134.18, 133.98, 128.12 (C_4_H_3_S), 95.01, 70.35, 69.70, 68.80, 67.44 (C_10_H_9_). MS (*m*/*z*, ESI−) 906.659 (M−). Anal. calcd for C_27_H_12_SO_11_FeRu_3_: 906.660.

#### [Ru(CO)_3_{μ_4_-η^1^:η^2^:η^1^:η^1^-(2-C_4_H_3_SC(O))CC(Fc)C(2-C_4_H_3_SC(O)) C(Fc)Ru(CO)_3_}μ-CO] (2b)

Red-brown powder. Yield: 13%. FT-IR (KBr, cm^−1^): 3060 w, 2088 vs, 2056 vs, 2017 vs, 1896 vs, 1409 m. ^1^H NMR (400 MHz, CDCl_3_) *δ* 7.68–7.70 (dd, 1H, C_4_H_3_S), 7.44–7.49 (m, 2H, C_4_H_3_S), 7.25–7.27 (m, 1H, C_4_H_3_S), 7.07–7.10 (m, 1H, C_4_H_3_S), 6.92–6.95 (m, 1H, C_4_H_3_S), 3.79–4.16 (m, 18H, C_10_H_9_). ^13^C{1H} NMR (101 MHz, CDCl_3_) *δ* 197.03, 196.00, 194.00, 193.12, 190.29, 185.92 (CO), 165.57, 158.05 (C≡C), 144.57, 142.28, 135.11, 134.26, 133.84, 133.13, 132.81, 128.97, 128.56, 127.68 (C_4_H_3_S), 97.06, 81.67, 76.00, 71.19, 70.97, 70.32, 69.83, 68.70, 68.53, 68.45, 68.24, 68.02, 67.69 (C_10_H_9_). MS (*m*/*z*, ESI−) 1010.771 (M−). Anal. Calcd for C_40_H_24_S_2_O_8_Fe_2_Ru_2_: 1010.771.

#### [Ru_3_(CO)_9_(μ-CO){μ_2_-η^1^:η^1^-(2-C_4_H_3_SC(O))CC(Fc)}OC{μ_3_-η^1^:η^2^-(Fc)CC(2-C_4_H_3_SC(O))}] (2c)

Black powder. Yield: 11%. FT-IR (KBr, cm^−1^): 3093 w, 2927 w, 2851 w, 2101 vs, 2061 vs, 2030 vs, 2010 vs, 1848 m. ^1^H NMR (400 MHz, CDCl_3_) *δ* 7.76–7.78 (d, 1H, C_4_H_3_S), 7.70 (s, 1H, C_4_H_3_S), 7.56–7.57 (dd, 1H, C_4_H_3_S), 7.41–7.42 (d, 1H, C_4_H_3_S), 7.25–7.25 (t, 1H, C_4_H_3_S), 6.98–7.00 (t, 1H, C_4_H_3_S), 4.78–4.80 (d, 2H, C_10_H_9_), 4.65–4.66 (d, 1H, C_10_H_9_), 4.15–4.40 (m, 15H, C_10_H_9_). ^13^C{1H} NMR (101 MHz, CDCl_3_) *δ* 195.61, 190.71, 188.75 (CO), 164.99 (C≡C), 143.92, 135.97, 134.15, 133.99, 133.08, 128.39, 128.21 (C_4_H_3_S), 82.65, 76.18, 71.77, 70.16, 69.71, 69.55, 69.12, 68.82, 68.66, 68.05 (C_10_H_9_). MS (*m*/*z*, ESI−) 1226.659 (M−). Anal. calcd for C_44_H_24_S_2_O_12_Fe_2_Ru_3_: 1226.656.

#### [Ru(CO)_3_{μ_4_-η^1^:η^2^:η^1^:η^1^-(Fc)CC(2-C_4_H_3_SC(O))C(2-C_4_H_3_SC(O)) C(Fc)Ru(CO)_3_}μ-CO] (2d)

Red-brown powder. Yield: 17%. FT-IR (KBr, cm^−1^): 3095 w, 2924 w, 2952 w, 2847 w, 2086 vs, 2054 vs, 2023 vs, 1999 vs, 1960 s, 1603 m, 1406 s. ^1^H NMR (400 MHz, CDCl_3_) *δ* 7.59–7.64 (m, 4H, C_4_H_3_S), 7.12–7.14 (m, 2H, C_4_H_3_S), 3.97–4.22 (m, 18H, C_10_H_9_). ^13^C{1H} NMR (101 MHz, CDCl_3_) *δ* 195.20, 192.97, 189.01 (CO), 164.99 (C≡C), 143.78, 133.41, 127.71, 124.81 (C_4_H_3_S), 83.76, 74.35, 72.00, 70.41, 69.83, 68.46, 67.67 (C_10_H_9_). MS (*m*/*z*, ESI−) 1010.763 (M−). Anal. calcd for C_40_H_24_S_2_O_8_Fe_2_Ru_2_: 1010.771.

#### [Ru_3_(CO)_9_(μ_2_-CO){μ_3_-η^1^:η^2^:η^1^-(Fc)CC(4-CH_3_O-PhC(O))}] (3a)

Red-brown powder. Yield: 11%. FT-IR (KBr, cm^−1^): 3091 w, 2961 w, 2926 w, 2842 w, 2099 s, 2051 vs, 2019 vs, 1853 m. ^1^H NMR (400 MHz, CDCl_3_) *δ* 8.23–8.26 (d, 2H, C_6_H_4_), 7.11–7.13 (d, 2H, C_6_H_4_), 4.15–4.25 (m, 4H, C_10_H_9_), 4.01 (s, 5H, C_10_H_9_), 3.95 (s, 3H, CH_3_O–). ^13^C{1H} NMR (101 MHz, CDCl_3_) *δ* 196.79 (CO), 170.31, 169.02 (C≡C), 165.00, 134.29, 127.36, 115.22 (C_6_H_4_), 96.99, 71.93, 71.09, 70.82, 70.52 (C_10_H_9_), 57.04 (CH_3_O–). MS (*m*/*z*, ESI−) 930.715 (M−). Anal. calcd for C_30_H_16_O_12_FeRu_3_: 930.715.

#### [Ru(CO)_3_{μ_4_-η^1^:η^2^:η^1^:η^1^-(4-CH_3_O-PhC(O))CC(Fc)C(4-CH_3_O-Ph-PhC(O))C(Fc)Ru(CO)_3_}μ-CO] (3b)

Red-brown powder. Yield: 18%. FT-IR (KBr, cm^−1^): 3091 w, 2960 w, 2928 w, 2842 w, 2081 vs, 2053 vs, 2014 vs, 1982 vs, 1594 s. ^1^H NMR (400 MHz, CDCl_3_) *δ* 7.90–7.92 (d, 2H, C_6_H_4_), 7.77–7.75 (d, 2H, C_6_H_4_), 6.95–6.97 (d, 2H, C_6_H_4_), 6.81–6.83 (d, 2H, C_6_H_4_), 3.98–4.25 (m, 18H, C_10_H_9_), 3.75–3.94 (m, 6H, CH_3_O–). ^13^C{1H} NMR (101 MHz, CDCl_3_) *δ* 198.96, 197.74, 197.04, 195.52, 194.91, 193.52 (CO), 165.43, 165.27, 164.25 (C≡C), 137.75, 133.15, 133.01, 131.24, 129.88, 129.25, 115.58, 114.71 (C_6_H_4_), 98.72, 83.34, 76.98, 72.46, 72.14, 71.96, 71.66, 71.12, 70.84, 70.04, 69.91, 69.78, 69.51, 68.85 (C_10_H_9_), 57.00, 56.80 (CH_3_O–). MS (*m*/*z*, ESI−) 1058.872 (M−). Anal. calcd for C_46_H_32_O_10_Fe_2_Ru_2_: 1058.879.

#### [Ru_3_(CO)_9_(μ-CO){μ_2_-η^1^:η^1^-(4-CH_3_O-PhC(O))CC(Fc)}OC{μ_3_-η^1^:η^2^ -(Fc)CC(4-CH_3_O-PhC(O))}] (3c)

Black powder. Yield: 15%. FT-IR (KBr, cm^−1^): 3091 w, 2964 w, 2930 w, 2852 w, 2097 vs, 2053 vs, 2007 vs, 1987 vs, 1853 m, 1595 s. ^1^H NMR (400 MHz, CDCl_3_) *δ* 7.74–7.92 (m, 4H, C_6_H_4_), 6.81–7.03 (m, 4H, C_6_H_4_), 3.95–4.36 (m, 18H, C_10_H_9_), 3.74–3.90 (m, 6H, CH_3_O–). ^13^C{1H} NMR (101 MHz, CDCl_3_) *δ* 198.96, 198.53, 197.74, 197.29, 197.04, 195.77, 193.52, 192.23, 190.67 (CO), 166.05, 165.27, 164.88, 164.80, 164.53, 164.25 (C≡C), 138.24, 137.75, 133.42, 133.34, 133.15, 133.00, 131.40, 131.23, 131.03, 129.88, 129.24, 115.76, 115.30, 114.70 (C_6_H_4_), 98.71, 83.78, 83.33, 77.69, 76.97, 73.39, 72.45, 72.13, 71.65, 71.48, 71.39, 71.12, 70.84, 70.71, 70.15, 70.09, 70.03, 69.91, 69.81, 69.77, 69.51, 69.15, 68.85 (C_10_H_9_), 57.02, 56.84 (CH_3_O–). MS (*m*/*z*, ESI−) 1273.763 (M−). Anal. calcd for C_50_H_32_O_14_Fe_2_Ru_3_: 1273.763.

#### [Ru(CO)_3_{μ_4_-η^1^:η^2^:η^1^:η^1^-(Fc)CC(4-CH_3_O-PhC(O))C(4-CH_3_O-PhC(O))C(Fc)Ru(CO)_3_}μ-CO] (3d)

Red-brown powder. Yield: 17%. FT-IR (KBr, cm^−1^): 3095 w, 2934 w, 2840 w, 2086 vs, 2056 vs, 2016 vs, 1598 s. 1H NMR (400 MHz, CDCl_3_) *δ* 7.84–8.01 (m, 4H, C_6_H_4_), 6.95–7.03 (m, 4H, C_6_H_4_), 3.95–4.29 (m, 18H, C_10_H_9_), 3.89 (s, 6H, CH_3_O–). ^13^C{1H} NMR (101 MHz, CDCl_3_) *δ* 197.29, 196.70, 195.49, 194.76, 192.22 (CO), 170.44, 164.45 (C≡C), 133.71, 133.42, 130.10, 125.78, 115.76, 114.67 (C_6_H_4_), 85.66, 75.68, 72.98, 71.83, 71.48, 71.12, 69.89, 68.92 (C_10_H_9_), 56.89 (CH_3_O–). MS (*m*/*z*, ESI−) 1058.873 (M−). Anal. Calcd for C_46_H_32_O_10_Fe_2_Ru_2_: 1058.879.

#### [Ru_3_(CO)_9_(μ_2_-CO){μ_3_-η^1^:η^2^:η^1^-(Fc)CC(4-NH_2_-PhC(O))}] (4a)

Red-brown powder. Yield: 7%. FT-IR (KBr, cm^−1^): 3455 w, 3355 w, 2098 s, 2059 vs, 2011 s, 1857 s. ^1^H NMR (400 MHz, CDCl_3_) *δ* 8.04–8.11 (m, 2H, C_6_H_4_), 6.81–6.83 (d, 2H, C_6_H_4_), 6.72–6.74 (d, 2H, –NH_2_), 4.20–4.73 (m, 18H, C_10_H_9_). ^13^C{1H} NMR (101 MHz, CDCl_3_) *δ* 194.37, 194.29 (CO), 152.51 (C≡C), 134.79, 134.54, 133.73, 133.26, 124.59, 122.10, 116.73, 115.17 (C_6_H_4_), 83.51, 71.84, 71.44, 71.26, 71.08, 70.82, 70.49, 69.74 (C_10_H_9_). MS (*m*/*z*, ESI−) 915.715 (M−). Anal. calcd for C_29_H_15_NO_11_FeRu_3_: 915.715.

#### [Ru(CO)_3_{μ_4_-η^1^:η^2^:η^1^:η^1^-(4-NH_2_-PhC(O))CC(Fc)C(4-NH_2_-PhC(O))C(Fc)Ru(CO)_3_}μ-CO] (4b)

Red-brown powder. Yield: 12%. FT-IR (KBr, cm^−1^): 3460 m, 3369 m, 2917 w, 2846 w, 2092 s, 2056 vs, 2025 s, 2009 s, 1852 m, 1593 s. ^1^H NMR (400 MHz, CDCl_3_) *δ* 7.70–7.72 (d, 4H, C_6_H_4_), 6.81 (s, 4H, –NH_2_), 6.59–6.72 (dd, 4H, C_6_H_4_), 4.24–4.94 (m, 18H, C_10_H_9_). ^13^C{1H} NMR (101 MHz, CDCl_3_) *δ* 192.21, 191.83 (CO), 168.55, 165.21, 161.54 (C≡C), 133.62, 128.92, 121.47, 115.50, 115.41 (C_6_H_4_), 72.03, 72.02, 71.81, 71.77, 71.46, 71.43, 71.35, 71.34, 71.28, 71.09, 70.09, 70.07, 70.05, 69.74, 69.32 (C_10_H_9_). MS (*m*/*z*, ESI−) 1028.878 (M−). Anal. calcd for C_44_H_30_N_2_O_8_Fe_2_Ru_2_: 1028.880.

#### [Ru_3_(CO)_9_(μ-CO){μ_2_-η^1^:η^1^-(4-NH_2_-PhC(O))CC(Fc)}OC{μ_3_-η^1^:η^2^-(Fc)CC(4-NH_2_-PhC(O))}] (4c)

Black powder. Yield: 15%. FT-IR (KBr, cm^−1^): 3483 m, 3373 m, 2084 vs, 2052 vs, 2021 vs, 1995 vs, 1946 s, 1591 s. ^1^H NMR (400 MHz, CDCl_3_) *δ* 7.70–7.86 (t, 4H, C_6_H_4_), 6.92 (s, 4H, –NH_2_), 6.59–6.72 (m, 4H, C_6_H_4_), 3.96–4.30 (m, 18H, C_10_H_9_). ^13^C{1H} NMR (101 MHz, CDCl_3_) *δ* 197.76, 196.88, 191.59, 191.05 (CO), 174.04, 168.38, 164.55, 159.21 (C≡C), 133.06, 133.05, 132.98, 132.95, 130.27, 129.94, 129.93, 125.16, 122.01, 115.52, 112.77 (C_6_H_4_), 73.38, 72.07, 72.03, 71.88, 71.76, 71.43, 71.09, 70.91, 70.85, 70.61 (C_10_H_9_). MS (*m*/*z*, ESI−) 1241.766 (M−). Anal. calcd for C_48_H_30_N_2_O_12_Fe_2_Ru_3_: 1241.763.

#### [Ru(CO)_3_{μ_4_-η^1^:η^2^:η^1^:η^1^-(Fc)CC(4-NH_2_-PhC(O))C(4-NH_2_-PhC(O))C(Fc)Ru(CO)_3_}μ-CO] (4d)

Red-brown powder. Yield: 19%. FT-IR (KBr, cm^−1^): 3477 m, 3370 m, 3224 w, 2083 vs, 2052 vs, 2020 vs, 1992 vs, 1590 s. ^1^H NMR (400 MHz, CDCl_3_) *δ* 7.70–7.87 (t, 4H, C_6_H_4_), 6.81–6.92 (d, 4H, –NH_2_), 6.59–6.72 (m, 4H, C_6_H_4_), 4.22–4.94 (m, 18H, C_10_H_9_). ^13^C{1H} NMR (101 MHz, CDCl_3_) *δ* 193.62, 191.82, 191.06 (CO), 153.77, 147.81, 146.14 (C≡C), 135.44, 134.53, 133.80, 133.78, 133.75, 133.49, 129.01, 115.50, 115.42, 115.37 (C_6_H_4_), 74.36, 73.63, 73.09, 72.03, 71.76, 71.43, 71.28, 71.09 (C_10_H_9_). MS (*m*/*z*, ESI−) 1031.880 (M−). Anal. calcd for C_44_H_30_N_2_O_8_Fe_2_Ru_2_: 1031.880.

#### [Ru_3_(CO)_9_(μ_2_-CO){μ_3_-η^1^:η^2^:η^1^-(4-NO_2_-PhC(O))CC(Ph)}] (5a)

Red-brown powder. Yield: 10%. FT-IR (KBr, cm^−1^): 3099 w, 2101 vs, 2056 vs, 2032 vs, 1855 m, 1524 s. ^1^H NMR (400 MHz, CDCl_3_) *δ* 8.39–8.48 (dd, 4H, C_6_H_4_), 3.97–4.28 (m, 18H, C_10_H_9_). ^13^C{1H} NMR (101 MHz, CDCl_3_) *δ* 194.11 (CO), 167.96, 165.40 (C≡C), 150.30, 138.52, 131.31, 123.74 (C_6_H_4_), 95.49, 70.26, 70.17, 70.14, 70.01, 69.88, 69.76, 69.65 (C_10_H_9_). MS (*m*/*z*, ESI−) 945.687 (M−). Anal. calcd for C_29_H_13_O_13_NFeRu_3_: 945.689.

#### [Ru(CO)_3_{μ_4_-η^1^:η^2^:η^1^:η^1^-(4-NO_2_-PhC(O))CC(Fc)C(4-NO_2_-PhC(O))C(Fc)Ru(CO)_3_}μ-CO] (5b)

Red-brown powder. Yield: 17%. FT-IR (KBr, cm^−1^): 3095 w, 2091 s, 2061 vs, 2022 vs, 1520 s. ^1^H NMR (400 MHz, CDCl_3_) *δ* 8.27–8.32 (t, 4H, C_6_H_4_), 8.04–8.11 (m, 4H, C_6_H_4_), 3.94–4.30 (m, 18H, C_10_H_9_). ^13^C{1H} NMR (101 MHz, CDCl_3_) *δ* 194.69, 192.60, 192.25 (CO), 150.03 (C≡C), 140.03, 130.65, 130.01, 123.69, 123.41 (C_6_H_4_), 84.01, 74.02, 71.21, 70.99, 70.69, 69.85, 69.50, 68.83, 68.17 (C_10_H_9_). MS (*m*/*z*, ESI−) 1091.826 (M−). Anal. calcd for C_44_H_26_O_12_N_2_Fe_2_Ru_2_: 1091.828.

#### [Ru(CO)_3_{μ_4_-η^1^:η^2^:-η^1^:η^1^-(4-NH_2_-PhC(O))CC(Ph)C(Ph)C(4-NO_2_-PhC(O))Ru(CO)_3_}μ-CO] (5c)

Red-brown powder. Yield: 12%. FT-IR (KBr, cm^−1^): 2091 s, 2055 vs, 2007 vs, 1595 m, 1528 m. ^1^H NMR (400 MHz, CDCl_3_) *δ* 7.77–8.32 (m, 8H, C_6_H_4_), 6.69–6.80 (dd, 2H, –NH_2_), 3.56–4.29 (m, 18H, C_10_H_9_). ^13^C{1H} NMR (101 MHz, CDCl_3_) *δ* 197.06 (CO), 151.10 (C≡C), 139.54, 133.12, 130.65, 130.07, 129.90, 123.50, 123.16 (C_6_H_4_), 71.51, 70.98, 70.69, 70.50, 70.30, 69.85, 69.82, 69.66, 69.15, 68.55, 68.53, 67.41 (C_10_H_9_). MS (*m*/*z*, ESI−) 1059.853 (M−). Anal. calcd for C_44_H_28_O_10_N_2_Fe_2_Ru_2_: 1059.853.

#### [Ru(CO)_3_{μ_4_-η^1^:η^2^:η^1^:η^1^-(Fc)CC(4-NH_2_-PhC(O))C(4-NO_2_-PhC(O))C(Fc)Ru(CO)_3_}μ-CO] (5d)

Red-brown powder. Yield: 21%. FT-IR (KBr, cm^−1^): 3481 w, 3343 w, 2087 vs, 2060 vs, 2020 vs, 1993 vs, 1961 vs, 1591 m, 1524 m. ^1^H NMR (400 MHz, CDCl_3_) *δ* 8.23–8.26 (d, 2H, C_6_H_4_), 7.56–8.04 (m, 6H, C_6_H_4_), 6.68–6.70 (d, 2H, –NH_2_), 3.95–4.25 (m, 18H, C_10_H_9_). ^13^C{1H} NMR (101 MHz, CDCl_3_) *δ* 195.43 (CO), 150.78, 149.75 (C≡C), 140.37, 130.62, 128.15, 126.01, 124.64, 124.42, 123.24 (C_6_H_4_), 73.95, 70.70, 70.56, 70.52, 70.29, 70.13, 69.76, 69.70, 68.75, 68.30, 68.10, 67.36 (C_10_H_9_). MS (*m*/*z*, ESI−) 1061.854 (M−). Anal. calcd for C_44_H_28_O_10_N_2_Fe_2_Ru_2_: 1061.854.

#### [Ru_3_(CO)_9_(μ_2_-CO){μ_3_-η^1^:η^2^:η^1^-(FcC(O))CC(Fc)}] (6a)

Red-brown powder. Yield: 29%. FT-IR (KBr, cm^−1^): 3087 m, 2080 w, 2056 w, 2017 m, 1970 w, 1931 m, 1574 vs, 818 vs*.*^1^H NMR (400 MHz, CDCl_3_) *δ* 4.94 (s, 2H, C_10_H_9_), 4.84 (s, 2H, C_10_H_9_), 4.57 (s, 2H, C_10_H_9_), 4.42 (s, 2H, C_10_H_9_), 4.35 (s, 5H, C_10_H_9_), 4.25 (s, 5H, C_10_H_9_). ^13^C{1H} NMR (101 MHz, CDCl_3_) *δ* 193.23, 191.57 (CO), 159.14, 158.20 (C≡C), 84.57, 78.57, 77.05, 76.30, 75.98, 75.67, 70.38, 69.17, 68.78, 68.67, 68.62, 68.50, 68.20, 68.13, 67.87, 67.40, 67.31, 67.29, 67.21, 67.07, 66.87, 66.43, 66.15, 65.59, 65.27, 64.95, 64.50, 63.62 (C_10_H_9_). MS (*m*/*z*, ESI−) 1018.749 (M−). Anal. calcd for C_33_H_28_O_11_Fe_2_Ru_3_: 1018.748.

### Crystallography

X-ray structural measurements were carried out with a Bruker D8 QUEST with a Photo 100 CMOS detector using graphite monochromated MoKα radiation (*λ* = 0.71073). The data were collected by the *ω*–2*θ* scan mode, and absorption correction was applied by using Multi-Scan. The structure was solved by direct methods (SHELXS-2014/97) and refined by full-matrix least squares against F2 using SHELXL-2014 and SHELXL-97 software.^26^ Non-hydrogen atoms were refined with anisotropic thermal parameters. All hydrogen atoms were geometrically fixed and refined using a riding model.

The single crystals of compounds 1b, 1d, 2a, 2b, 2c, 2d, 4a, 4d and 5d suitable for single crystal X-ray diffraction were successfully grown up from their dichloromethane/hexane solutions after slow evaporation at 0–5 °C. Relevant crystallographic data were given in Table S1 in the ESI.†

## Conclusions

We obtained a series of new ruthenium clusters by investigating reactions of the ferrocenyl containing 1,3-ynones 1–6 with Ru_3_(CO)_12_. Some new clusters with unexpected structures were isolated while some anticipated products were not formed, although most of the clusters exhibit a similar skeleton to those of the products *via* reaction of 1,3-diphenylprop-2-yn-1-one derivatives with Ru_3_(CO)_12_. An electron-withdrawing group at the carbonyl side of an alkynyl ketone is beneficial to the formation of normal ruthenoles b, c and d; while an electron-donating group favors the production of normal ruthenoles b and d, but disfavors the formation of ruthenole c; the larger steric hindrance and electron-donating effect of two ferrocenyl groups in 6 prefers only the formation of 6a. In addition, we believe that the reduction of half of the nitro groups in both 5c and 5d was driven by both the electron-donating ferrocenyl group and CO in the presence of the catalyst Ru_3_(CO)_12_. No formation of the expected cyclotrimerization products of the 1,3-ynones can also be ascribed to the unusual properties of the ferrocenyl groups in 1–6. The reaction between Ru_3_(CO)_12_ and a 1,3-ynone with a ferrocenyl group at its C≡C side has given some unexpected results, which promotes us to investigate the reaction of Ru_3_(CO)_12_ with alkynyl ketones containing ferrocenyl groups at their carbonyl sides; this study is already underway.

## Computational details

The optimization used DFT method with the Becke's three parameter hybrid functional and Lee Yang Parr's gradient corrected correlation functional (B3LYP).^27^ Calculations were performed with the GAUSSIAN-09 program.^28^ The LanL2DZ basis set and effective core potential were used for the Fe and Ru atoms, and the 6-31G basis sets were used for all other atoms, respectively.^29^ The nature of all stationary points were confirmed by performing a normal-mode analysis. The input model molecules for 5c and the predicted 5c′ were based on the head-to-head coupled ruthenoles we reported earlier^17^ and the structure of 2c was also optimized.

## Conflicts of interest

No conflicts of interest to declare.

## Supplementary Material

RA-008-C8RA04548H-s001

RA-008-C8RA04548H-s002
