# Supplementary material for: Reaction of FcC[triple bond, length as m-dash]CC(O)R (Fc = ferrocenyl) with Ru3(CO)12 leading to unexpected nitro-group reduced ruthenoles and 1,2-CO-inserted triruthenium clusters
Source: RSC Adv. 2018 Jul 16;8(45):25268–76. doi: 10.1039/c8ra04548h (PMC9082558; doi:10.1039/c8ra04548h)
Supplement: RA-008-C8RA04548H-s001 [file RA-008-C8RA04548H-s001.pdf]

Electronic Supplementary Material (ESI) for RSC Advances.
This journal is © The Royal Society of Chemistry 2018

Reaction of FcC≡CC(O)R (Fc=ferrocenyl) with $Ru_3(CO)_{12}$ leading to unexpected nitro-group reduced ruthenoles and 1,2-CO-inserted triruthenium clusters

Lei Xu, Liping Jiang, Shasha Li, Guofang Zhang, Weiqiang Zhang and Ziwei Gao

Key Laboratory of Applied Surface and Colloid Chemistry, School of Chemistry and Chemical Engineering, Shaanxi Normal University, Xi'an 710119, China

Table S1. Crystal and refinement data of **1b**, **1d**, **2a**, **2b**, **2c**, **2d**, **4a**, **4d** and **5d**..............S1

Table S2. The geometries parameter of **2c**...S4

Table S3. The geometries parameter of **5c**...S8

Fig. S1. The crystal structure of compound **1d**...S12

Fig. S2. The crystal structure of compound **2a**...S13

Fig. S3. The crystal structure of compound **2b**·$1/2C_6H_{14}$...S14

Fig. S4. The crystal structure of compound **2c**...S15

Fig. S5. The crystal structure of compound **4d**...S16

Fig. S6. The DFT-optimized structure of compound **5c**...S17

^{1}H NMR spectra of all new compounds...S18

^{13}C{1H} NMR spectra of all new compounds...S29

FT-IR spectra of all new compounds...S40

Table S1. Crystal and refinement data of **1b**, **1d**, **2a**, **2b**, **2c**, **2d**, **4a**, **4d** and **5d**.

Compounds	**1b**	**1d**	**2a**
formula	$C_{44}H_{28}Fe_2O_8Ru_2$	$C_{44}H_{28}Fe_2O_8Ru_2$	$C_{27}H_{12}FeO_{11}Ru_3S$
Fw(g mol^{-1})	998.50	998.50	903.49
Wavelength(Å)	0.71073	0.71073	0.71073
T (K)	296(2)	296(2)	153(2)
crystal system	Triclinic	Monoclinic	Monoclinic
space group	P-1	P2(1)/c	P2(1)/c
a (Å)	12.420(2)	9.8872(5)	17.659(3)
b (Å)	12.656(2)	24.2002(12)	10.0342(19)
c (Å)	14.050(2)	15.6004(8)	33.191(7)
α(°)	94.236(5)	90.00	90.00
β(°)	106.349(5)	96.503(2)	101.971(5)
γ (°)	107.077(5)	90.00	90.00
V (Å3)	1996.0(5)	3708.7(3)	5753.4(19)
Z	2	4	8
Dc (Mg m^{-3})	1.661	1.788	2.086
Absorption coefficient (mm^{-1})	1.507	1.622	2.172
F(000)	992	1984	3488
Crystal sizes (mm)	0.17×0.13×0.11	0.13×0.12×0.10	0.14×0.13×0.10
collected/unique	69759/7831	89603/7292	76940/5660
R_{int}	0.0396	0.0530	0.0376
Data/restraints/parameters	7831/0/ 505	7292/0/505	5660/0/388
GOF on *F*2	1.108	1.074	1.193
R1, wR2 *[I ≥2σ(I)]*	0.0294, 0.0695	0.0245, 0.0501	0.0255, 0.0497
R1, wR2 *(all data)*	0.0323, 0.0695	0.0315, 0.0529	0.0301, 0.0511
largestdiff. Peak/hole (e. Å^{-3})	0.412, -0.599	0.383,-0.501	0.504,-0.503

Table S1. Crystal and refinement data of **1b**, **1d**, **2a**, **2b**, **2c**, **2d**, **4a**, **4d** and **5d**.

Complex	**2b**	**2c**	**2d**
formula	$C_{43}H_{31}Fe_2O_8Ru_2S_2$	$C_{5.03}H_{2.74}Fe_{0.23}O_{1.37}$ $Ru_{0.34}S_{0.23}$	$C_{40}H_{24}Fe_2O_8Ru_2S_2$
Fw(g mol^{-1})	1053.64	139.85	1010.55
Wavelength(Å)	0.71073	0.71073	0.71073
T (K)	296(2)	153(2)	154(2)
crystal system	Triclinic	Monoclinic	Monoclinic
space group	P-1	P2(1)/c	P2(1)/c
a (Å)	11.9891(10)	14.508(4)	12.0452(5)
b (Å)	12.0802(10)	8.676(3)	18.9355(8)
c (Å)	15.8425(14)	34.637(10)	16.7320(8)
α(°)	92.359(3)	90.00	90.00
β(°)	109.803(2)	99.749(10)	107.767(2)
γ (°)	108.441(3)	90.00	90.00
V (Å3)	2019.5(3)	4297(2)	3634.2(3)
Z	2	35	4
Dc (Mg m^{-3})	1.733	1.892	1.847
Absorption coefficient (mm^{-1})	1.594	1.850	1.768
F(000)	1050	2400	2000
Crystal sizes (mm)	0.15×0.13×0.10	0.15×0.14×0.12	0.17×0.13×0.11
collected/unique	56992/7909	67291/7879	120716/7138
R_{int}	0.0555	0.0551	0.0497
Data/restraints/parameters	7909/7/515	7879/172/552	7138/24/487
GOF on *F*2	1.097	1.094	1.097
R1, wR2 *[I ≥2σ(I)]*	0.0435, 0.0911	0.1088, 0.2718	0.0259, 0.0614
R1, wR2 *(all data)*	0.0601, 0.1041	0.1147, 0.2750	0.0298, 0.0632
largestdiff. Peak/hole(e. Å^{-3})	1.148, -0.839	6.411,-3.011	1.390,-0.816

Table S1. Crystal and refinement data of **1b**, **1d**, **2a**, **2b**, **2c**, **2d**, **4a**, **4d** and **5d**.

Complex	**4a**	**4d**	**5d**
formula	$C_{29}H_{15}FeNO_{11}Ru_3$	$C_{44}H_{30}Fe_2N_2O_8Ru_2$	$C_{45}H_{30}Cl_2Fe_2N_2O_{10}Ru_2$
Fw(g mol^{-1})	912.48	1028.54	1143.45
Wavelength(Å)	0.71073	0.71073	0.71073
T (K)	153(2)	153(2)	153(2)
crystal system	Orthorhombic	Monoclinic	Orthorhombic
space group	Pbca	C2/c	Pbca
a (Å)	13.995(4)	29.747(9)	22.8840(19)
b (Å)	11.729(4)	19.893(6)	15.4397(13)
c (Å)	34.893(12)	15.727(6)	23.772(2)
α(°)	90.00	90.00	90.00
β(°)	90.00	114.924(12)	90.00
γ (°)	90.00	90.00	90.00
V (Å3)	5727(3)	8440(5)	8399.2(12)
Z	8	8	8
Dc (Mg m^{-3})	2.116	1.619	1.808
Absorption coefficient (mm^{-1})	2.113	1.430	1.573
F(000)	3536	4096	4544
Crystal sizes (mm)	0.18×0.13×0.10	0.15×0.13×0.10	0.15×0.13×0.09
collected/unique	63513/5636	48133/8281	256536/8244
R_{int}	0.0390	0.0677	0.1323
Data/restraints/parameters	5636/ 0/406	8281/44/523	8244/0/568
GOF on *F*2	0.888	1.072	1.064
R1, wR2 *[I ≥2σ(I)]*	0.0270, 0.0592	0.0533, 0.1256	0.0248, 0.0553
R1, wR2 *(all data)*	0.0390, 0.0651	0.0720, 0.1396	0.0319, 0.0594
largestdiff. Peak/hole (e. $Å^{-3}$)	0.798, -0.515	1.489,-1.500	0.548,-0.761

Table S2. The geometries parameter of **2c**.

B3LYP/LanL2DZ/6-31G

Center	Atomic	Atomic	Coordinates (Angstroms)		
Number	Number	Type	X	Y	Z
1	44	0	-0.599579	-1.549890	1.534200
2	44	0	-2.150053	-2.399389	-0.658424
3	44	0	0.624377	-1.589450	-1.141122
4	26	0	-2.062340	3.786692	0.571448
5	26	0	3.977356	3.102761	-0.362217
6	16	0	-6.315861	0.620176	0.481913
7	6	0	-1.340872	1.842445	1.163336
8	6	0	-2.454871	2.259533	2.007111
9	1	0	-3.384602	1.739734	2.163828
10	6	0	-2.100482	3.476730	2.670776
11	1	0	-2.731920	4.022571	3.358680
12	6	0	-0.780906	3.854091	2.244522
13	1	0	-0.239169	4.741070	2.550040
14	6	0	-0.316006	2.870086	1.313396
15	1	0	0.635264	2.887317	0.813058
16	6	0	-2.580111	3.919097	-1.484371
17	1	0	-2.481602	3.116809	-2.201830
18	6	0	-3.737157	4.195794	-0.670536
19	1	0	-4.660868	3.639074	-0.665777
20	6	0	-3.438240	5.343949	0.144127
21	1	0	-4.094613	5.788604	0.880730
22	6	0	-2.098197	5.774064	-0.164310
23	1	0	-1.573487	0.301766	6.602532
24	6	0	-1.568397	4.886497	-1.166158
25	1	0	-0.579334	4.945541	-1.614189

26	6	0	-1.197607	0.535742	0.441854
27	6	0	-2.240329	-0.452363	0.336826
28	6	0	-3.638186	-0.231617	0.822325
29	6	0	-4.597827	0.401835	-0.091429
30	6	0	-4.450835	0.851914	-1.390321
31	1	0	-3.508490	0.794216	-1.927370
32	6	0	-5.653125	1.373560	-1.968174
33	1	0	-5.713110	1.757910	-2.980071
34	6	0	-6.729374	1.331802	-1.113285
35	1	0	-7.746777	1.657453	-1.295633
36	6	0	3.576021	1.190503	0.469825
37	6	0	3.541313	2.202172	1.508237
38	1	0	2.687801	2.434245	2.130790
39	6	0	4.833346	2.823185	1.565498
40	1	0	5.121017	3.624810	2.231688
41	6	0	5.670513	2.211552	0.564124
42	1	0	6.695914	2.481513	0.343537
43	6	0	4.899351	1.217892	-0.117436
44	1	0	5.240355	0.601247	-0.943084
45	6	0	2.442075	3.669514	-1.713807
46	1	0	1.532348	3.107608	-1.864097
47	6	0	2.644087	4.709377	-0.734294
48	1	0	1.902980	5.072291	-0.032612
49	6	0	4.005645	5.166574	-0.843730
50	1	0	4.472019	5.931524	-0.234710
51	6	0	4.646303	4.409775	-1.889109
52	1	0	5.677801	4.504446	-2.200412
53	6	0	3.681880	3.484714	-2.426028
54	1	0	3.857351	2.766471	-3.217092

55	6	0	2.480459	0.299426	0.097092
56	6	0	2.510397	-0.954197	-0.440674
57	6	0	3.727704	-1.740118	-0.759038
58	6	0	4.646987	-2.106721	0.315326
59	16	0	6.180165	-3.006862	-0.115135
60	6	0	4.562074	-1.910485	1.684553
61	1	0	3.727683	-1.395668	2.154073
62	6	0	5.673483	-2.436623	2.422381
63	1	0	5.765469	-2.368625	3.502721
64	6	0	6.617038	-3.042718	1.626165
65	1	0	7.542920	-3.521695	1.922114
66	6	0	0.113524	0.042912	-0.016376
67	6	0	-1.771001	-1.989521	3.043862
68	6	0	0.642895	-0.752775	2.768506
69	6	0	0.304742	-3.232364	1.409449
70	6	0	-3.167291	-3.261821	0.743879
71	6	0	-3.677363	-2.274640	-1.866532
72	6	0	-1.659247	-4.173182	-1.330183
73	6	0	-1.114467	-1.239795	-2.227214
74	6	0	1.283547	-3.400030	-1.652363
75	6	0	1.424790	-0.832738	-2.714273
76	8	0	-4.021022	-0.655881	1.948429
77	8	0	3.939335	-2.150365	-1.938701
78	8	0	-2.348247	-2.272955	4.020424
79	8	0	1.438595	-0.275448	3.490530
80	8	0	0.801574	-4.300582	1.468043
81	8	0	-3.839736	-3.853698	1.500774
82	8	0	-4.609522	-2.256862	-2.575628
83	8	0	-1.398040	-5.232437	-1.756517

84	8	0	-1.486975	-0.604500	-3.170396
85	8	0	1.704290	-4.458842	-1.921810
86	8	0	1.866135	-0.307631	-3.664281
87	8	0	1.209773	0.863715	0.310315

Table S3. The geometries parameter of **5c**.

B3LYP/LanL2DZ/6-31G

Center	Atomic	Atomic	Coordinates (Angstroms)		
Number	Number	Type	X	Y	Z
1	44	0	-0.762950	-1.178198	1.394063
2	44	0	-0.728721	-1.177692	-1.446095
3	26	0	3.047435	-3.703959	-0.190909
4	26	0	-4.822902	0.799434	-0.159900
5	8	0	2.199015	0.474328	2.745697
6	8	0	6.849633	5.121902	0.187283
7	8	0	5.988108	4.847364	-1.900345
8	8	0	-0.625875	2.307066	2.691666
9	8	0	0.858070	-2.784646	3.554655
10	8	0	-2.945276	-0.514574	3.555877
11	8	0	-2.227243	-3.757157	0.396686
12	8	0	0.444026	0.815318	-3.545945
13	8	0	0.148549	-3.868718	-2.922580
14	8	0	-3.481105	-1.795268	-2.941145
15	7	0	6.015748	4.589040	-0.637696
16	7	0	0.166444	7.492425	-1.196511
17	1	0	0.166096	8.360291	-0.650071
18	1	0	0.283428	7.568635	-2.212593
19	6	0	2.352638	-1.620480	-0.294003
20	6	0	2.974634	-2.037103	-1.568146
21	1	0	2.488237	-2.019377	-2.553236
22	6	0	4.359134	-2.417786	-1.313455
23	1	0	5.077124	-2.773679	-2.067275
24	6	0	4.611446	-2.275677	0.118788
25	1	0	5.549193	-2.520173	0.639815

26	6	0	3.384989	-1.802757	0.751073
27	1	0	3.250981	-1.633526	1.827573
28	6	0	1.483305	-5.154194	0.196259
29	1	0	0.406883	-4.939448	0.266031
30	6	0	2.208878	-5.522303	-1.019624
31	1	0	1.778288	-5.609025	-2.026875
32	6	0	3.610540	-5.730497	-0.661428
33	1	0	3.610540	-5.730497	-0.661428
34	6	0	3.751229	-5.492002	0.775649
35	1	0	4.687387	-5.544778	1.351868
36	6	0	2.435180	-5.136709	1.306860
37	1	0	2.206521	-4.880591	2.351914
38	6	0	0.948129	0.302581	0.644749
39	6	0	1.031025	-0.899205	-0.177787
40	6	0	2.100459	0.826330	1.529613
41	6	0	3.107612	1.794866	0.926284
42	6	0	4.057048	2.417187	1.806403
43	1	0	4.020277	2.170577	2.882179
44	6	0	5.015150	3.332877	1.300711
45	1	0	5.749133	3.828501	1.957856
46	6	0	5.016562	3.614506	-0.096965
47	6	0	4.096837	3.005266	-0.996117
48	1	0	4.139594	3.250759	-2.070094
49	6	0	3.138045	2.093556	-0.475705
50	1	0	2.420405	1.612725	-1.163032
51	6	0	-2.683468	1.270183	-0.338622
52	6	0	-3.342243	2.098122	0.694862
53	1	0	-3.088190	2.116180	1.763495
54	6	0	-4.393931	2.884559	0.062117

55	1	0	-5.068998	3.585170	0.575867
56	6	0	-5.068998	3.585170	0.575867
57	1	0	-5.121732	2.946118	-2.108465
58	6	0	-3.393536	1.542179	-1.604501
59	1	0	-3.153939	1.101649	-2.581796
60	6	0	-5.237181	-1.225930	0.486492
61	1	0	-4.485144	-1.997885	0.704558
62	6	0	-5.786552	-0.267664	1.445257
63	1	0	-5.500501	-0.175012	2.503226
64	6	0	-6.760027	0.565534	0.738047
65	1	0	-7.334425	1.397743	1.172641
66	6	0	-6.811086	0.120043	-0.654945
67	1	0	-7.431931	0.556495	-1.452264
68	6	0	-5.870696	-0.987510	-0.810535
69	1	0	-5.666736	-1.535230	-1.741139
70	6	0	-0.322326	1.058207	0.608943
71	6	0	-1.390924	0.502586	-0.210067
72	6	0	-0.433200	2.369846	1.428326
73	6	0	-0.272123	3.673094	0.710985
74	6	0	-0.116171	3.774707	-0.716412
75	1	0	-0.121495	2.861976	-1.339295
76	6	0	0.027052	5.030218	-1.351517
77	1	0	0.138067	5.085809	-2.451667
78	6	0	0.022950	6.246795	-0.575177
79	6	0	-0.133399	6.151173	0.859093
80	1	0	-0.139021	7.076248	1.467431
81	6	0	-0.279246	4.892544	1.480216
82	1	0	-0.402408	4.817058	2.576120
83	6	0	0.243098	-2.166533	2.738937

84	6	0	-2.119005	-0.766911	2.732351
85	6	0	-1.636946	-2.709718	0.518048
86	6	0	-0.019770	0.028696	-2.770221
87	6	0	-2.500045	-1.519053	-2.324466
88	6	0	-0.116786	-2.883901	-2.307534

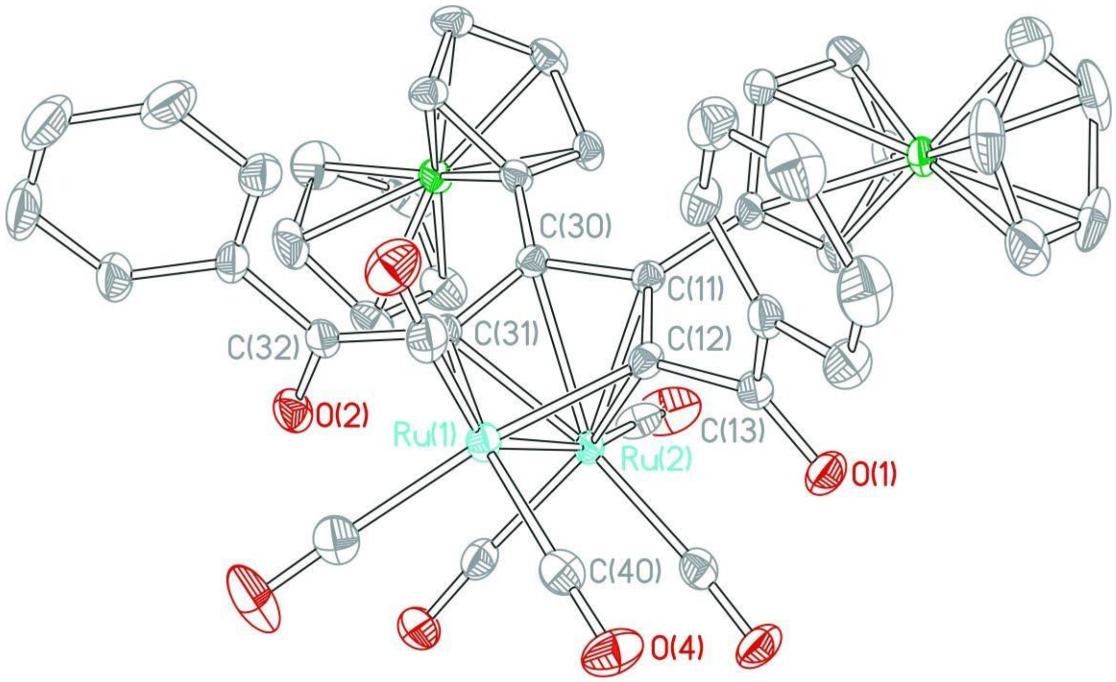

ORTEP view of cluster **1d** showing 50% ellipsoids. Selected bond lengths (Å) and bond angles (°): Ru1-Ru2 = 2.7141(3), Ru1-C31 = 2.0957(21), Ru1-C12 = 2.0668(21), Ru2-C11 = 2.2647(22), Ru2-C12 = 2.2655(21), Ru2-C30 = 2.2848(22), Ru2-C31 = 2.2717(22), C30-C31 = 1.4151(30), C11-C30 = 1.4868(31), C11-C12 = 1.4088(30), C12-C13 = 1.4916(31), C31-C32 = 1.4979(31), C32-O2 = 1.2207(29), C13-O1 = 1.2195(28).

Fig. S1. The crystal structure of compound **1d**.

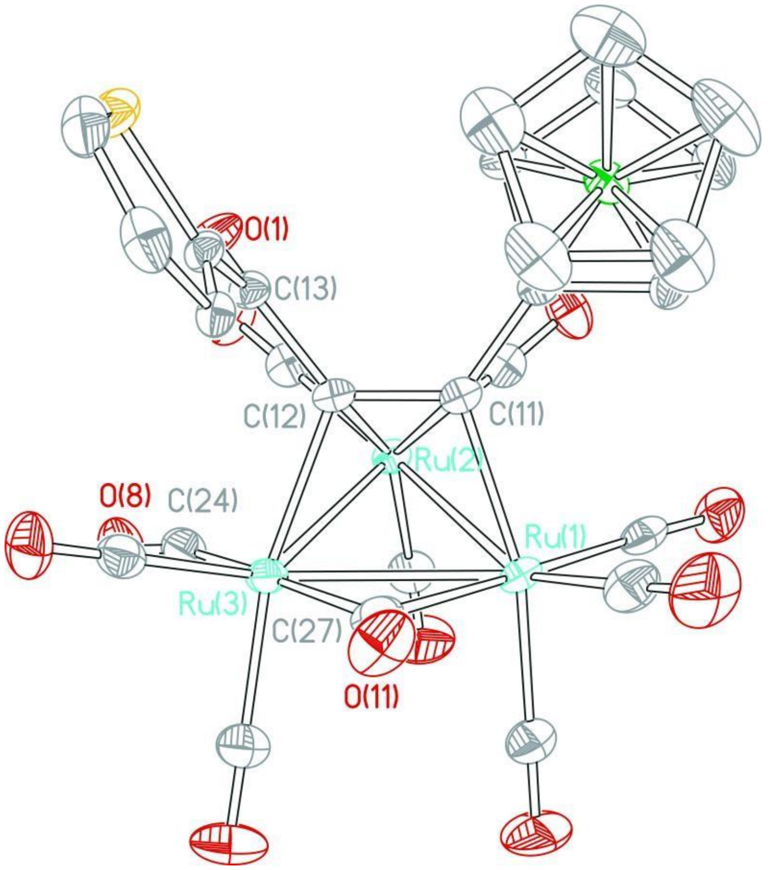

ORTEP view of cluster **2a** showing 50% ellipsoids. Selected bond lengths (Å) and bond angles (°): Ru1-Ru2 = 2.7500(5), Ru2-Ru3 = 2.7282(5), Ru1-Ru3 = 2.8111(5), Ru1-C11 = 2.0969(25), Ru1-C27 = 2.2763(33), Ru2-C11 = 2.3335(28), Ru2-C12 = 2.2058(26), Ru3-C12 = 2.1357(28), Ru3-C27 = 2.0583(32), Ru3-C24 = 1.9336(33), C11-C12 = 1.4034(42), C27-O11 = 1.1531(42), C24-O8 = 1.1469(39), Ru1-C27-Ru3 = 80.691(105), Ru2-C24-Ru3 = 70.633(99).

Fig. S2. The crystal structure of compound **2a**.

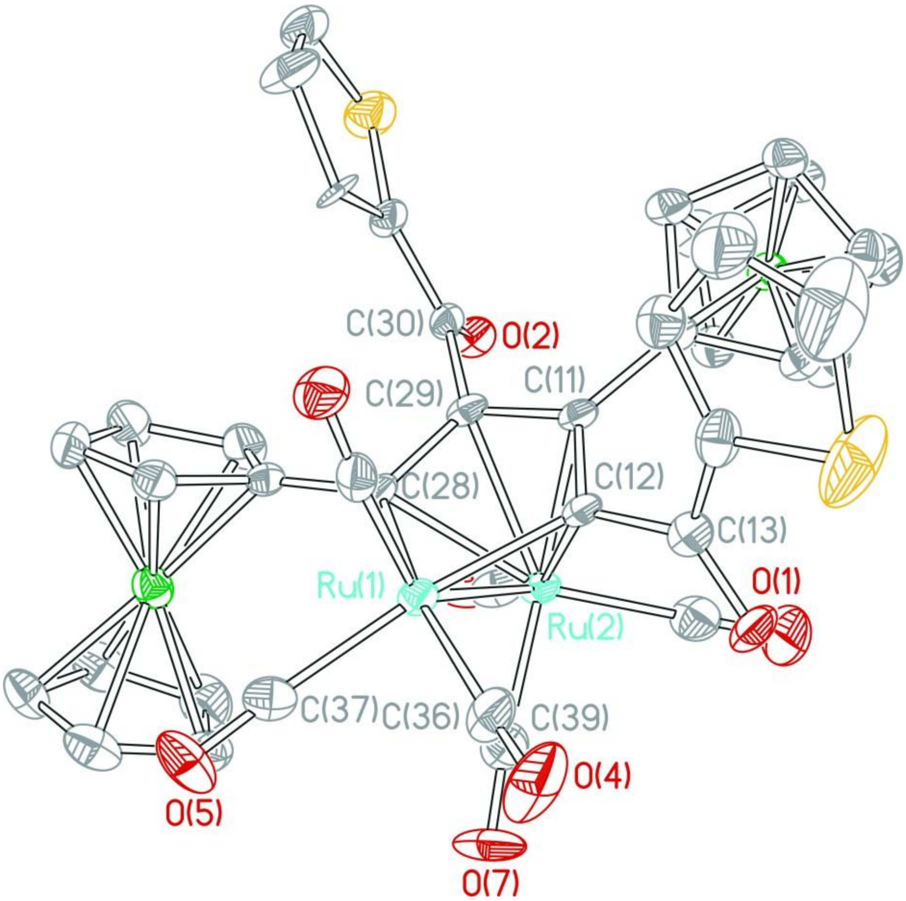

ORTEP view of cluster **2b**·1/2C_6H_{14} showing 50% ellipsoids (Solvent molecules have been omitted for clarity). Selected bond lengths (Å) and bond angles (°): Ru1-Ru2 = 2.7517(5), Ru1-C28 = 2.0948(53), Ru1-C12 = 2.0805(55), Ru2-C11 = 2.2810(46), Ru2-C12 = 2.2071(40), Ru2-C28 = 2.2790(4), Ru2-C29 = 2.2594(44), C28-C29 = 1.4209(80), C11-C29 = 1.4507(70), C11-C12 = 1.4225(78), C12-C13 = 1.4845(75), C29-C30 = 1.5273(77), C30-O2 = 1.2094(50), C13-O1 = 1.2298(56), Ru1-C39-Ru2 = 68.077(184).

Fig. S3. The crystal structure of compound **2b**·1/2C_6H_{14}.

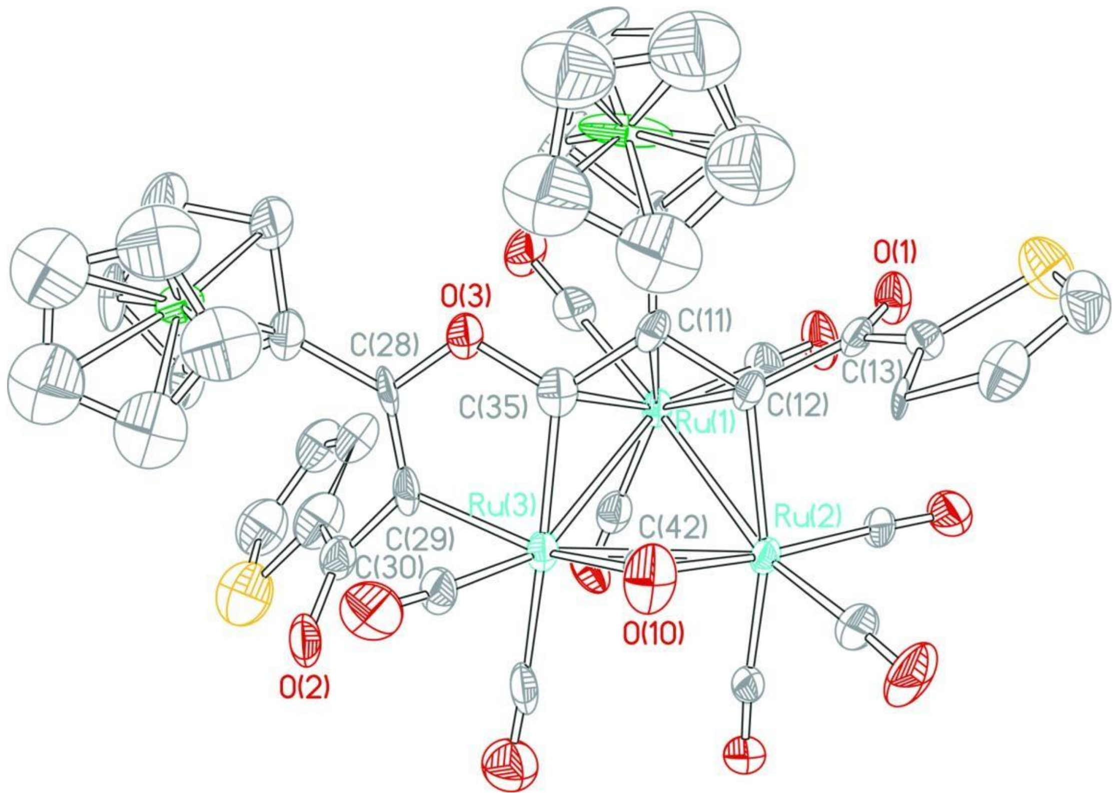

ORTEP view of cluster **2c** showing 50% ellipsoids. Selected bond lengths (Å) and bond angles (°): Ru1-Ru2 = 2.7681(17); Ru2-Ru3 = 2.8236(16); Ru1-Ru3 = 2.8212(16); Ru1-C11 = 2.3280(15); Ru1-C35 = 2.2528(17); Ru1-C12 = 2.2437(15); Ru2-C42 = 2.2220(17); Ru3-C29 = 2.0750(14); Ru3-C35 = 2.0356(16); C11-C12 = 1.4295(19); C11-C35 = 1.4450(21); C35-O3 = 1.3687(18); C28-O3 = 1.3726(187); C28-C29 = 1.3615(22); C29-C30 = 1.4899(22); C30-O2 = 1.2261(19); C12-C13= 1.4987(19); C13-O1 = 1.2347(17); Ru2-C42-Ru3 = 83.226(62); Ru3-C35-O3 = 115.182(10); Ru3-C35-C11 = 128.792(11).

Fig. S4. The crystal structure of compound **2c**.

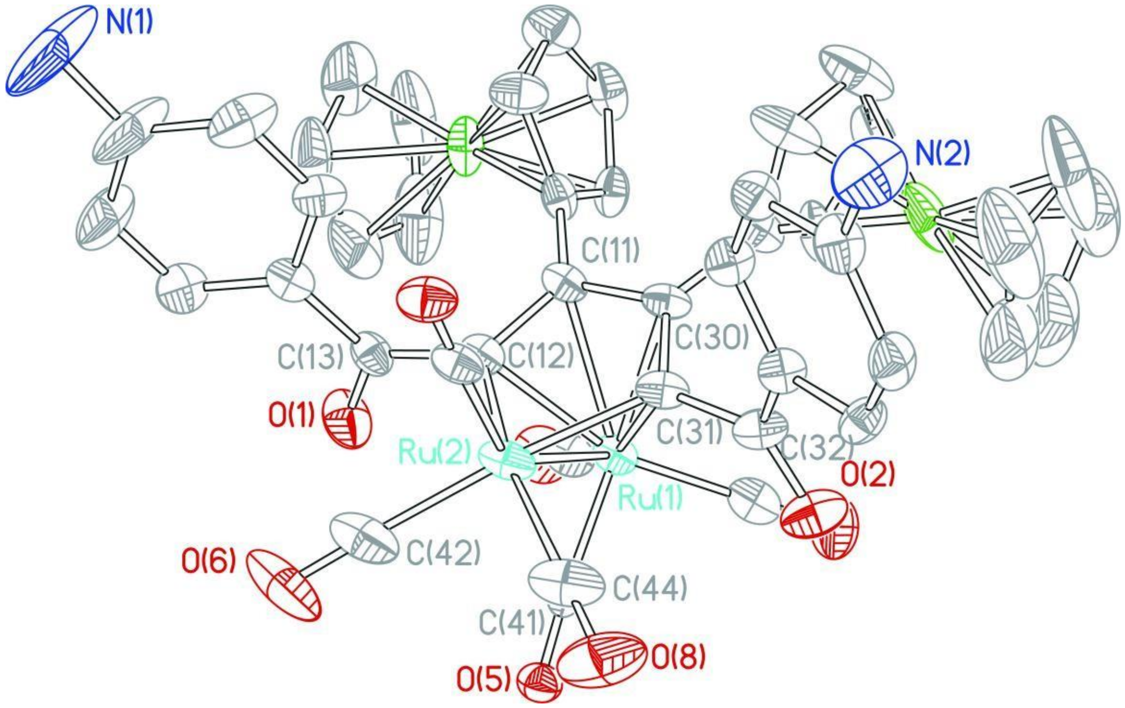

ORTEP view of cluster **4d** showing 50% ellipsoids. Selected bond lengths (Å) and bond angles (°): Ru1-Ru2 = 2.7165(9), Ru1-C11 = 2.2893(73), Ru1-C12 = 2.2202(68), Ru1-C30 = 2.2714(61), Ru1-C31 = 2.2192(53), Ru1-C41 = 1.8915(63), Ru2-C12 = 2.0853(52), Ru2-C31 = 2.0767(55), Ru2-C41 = 2.6670(66), C11-C12 = 1.4268(61), C11-C30 = 1.4568(77), C30-C31 = 1.4303(90), C12-C13 = 1.4880(73), C31-C32 = 1.4927(79), C32-O2 = 1.2360(93), C13-O1 = 1.2374(70), Ru1-C41-Ru2 = 70.839(183), Ru1-C41-O5 = 169.979(509).

Fig. S5. The crystal structure of compound **4d**.

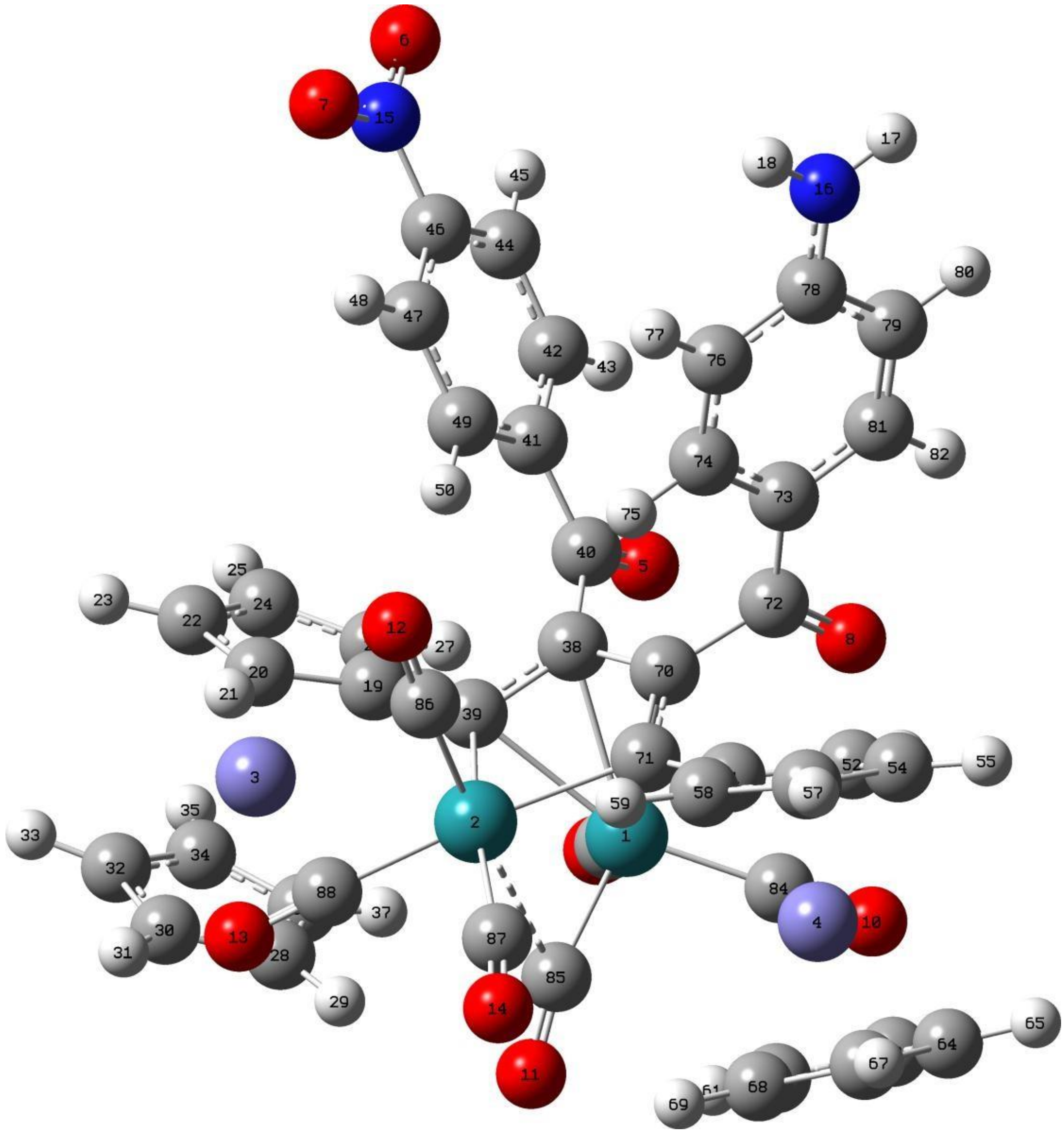

DFT-optimized structure of **5c** at the level of B3LYP/LanL2DZ/6-31G. Selected bond lengths (Å) and bond angles (°): Ru1-Ru2 = 2.7885, Ru1-C39 = 2.3595, Ru1-C38 = 2.3436, Ru1-C70 = 2.3392, Ru1-C71 = 2.3729, Ru1-C85 = 1.9265, Ru2-C39 = 2.1380, Ru2-C71 = 2.1448, Ru2-C85 = 2.6546, C38-C39 = 1.4303, C38-C70 = 1.4502, C70-C71 = 1.4308, Ru1-C85-Ru2 = 73.04.

Fig. S6. The DFT-optimized structure of compound **5c**.

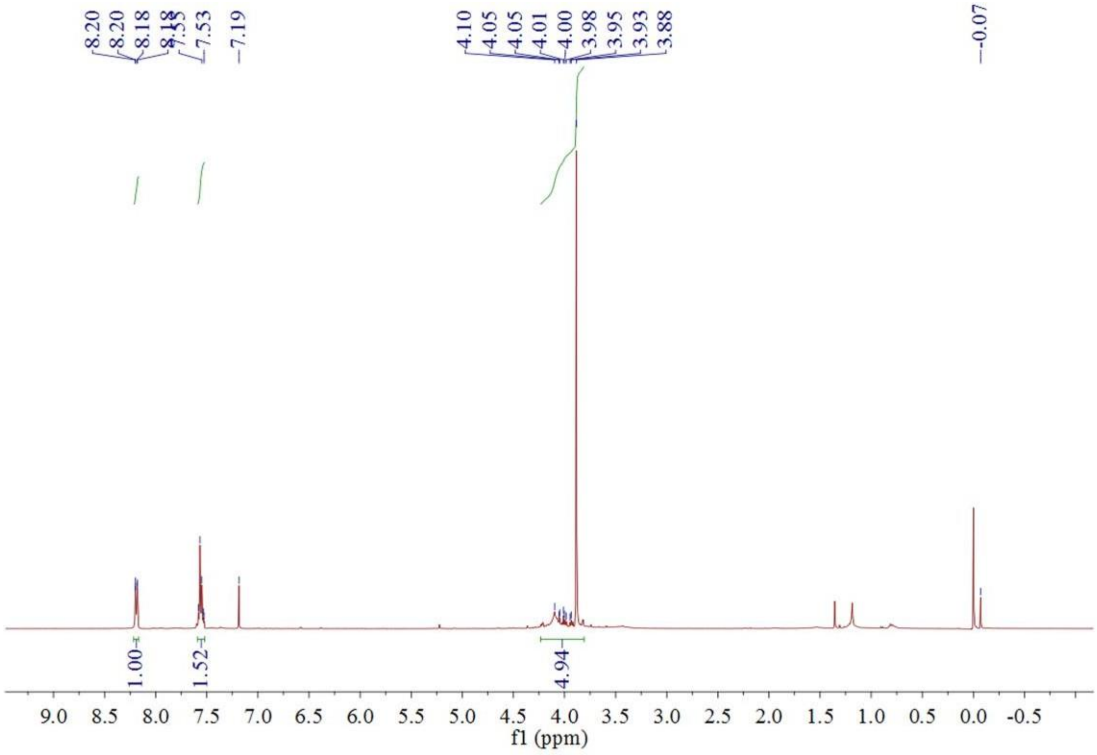

^{1}H NMR spectrum of compound **1a**.

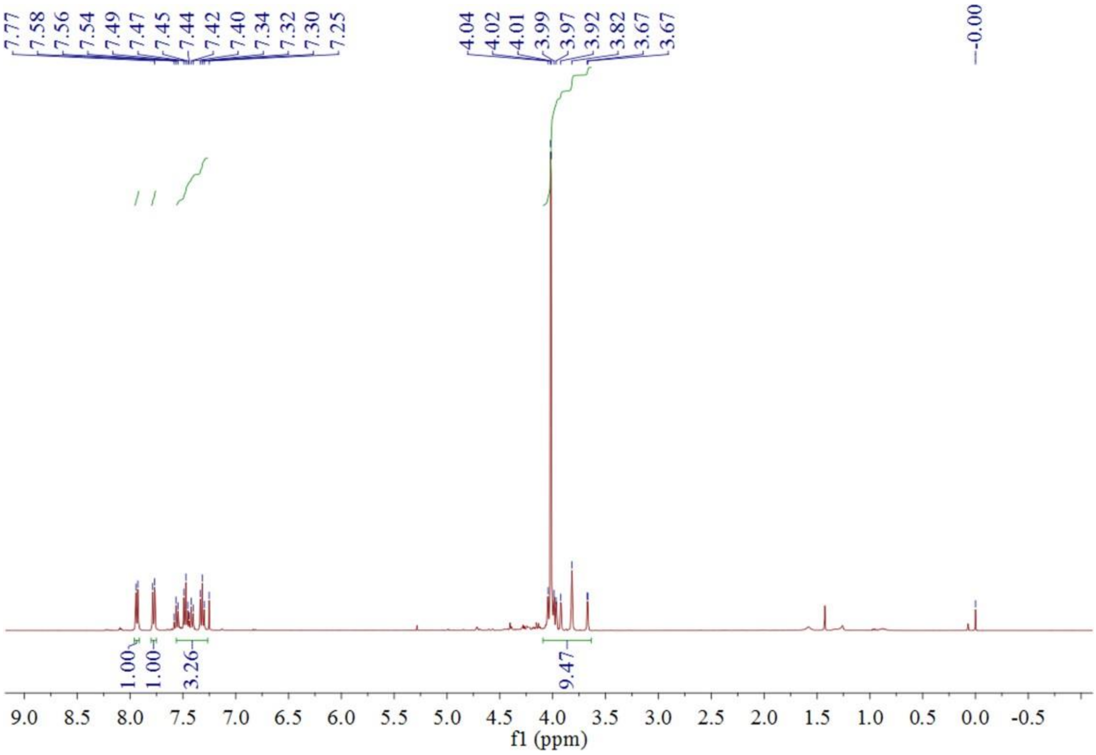

^{1}H NMR spectrum of compound **1b**.

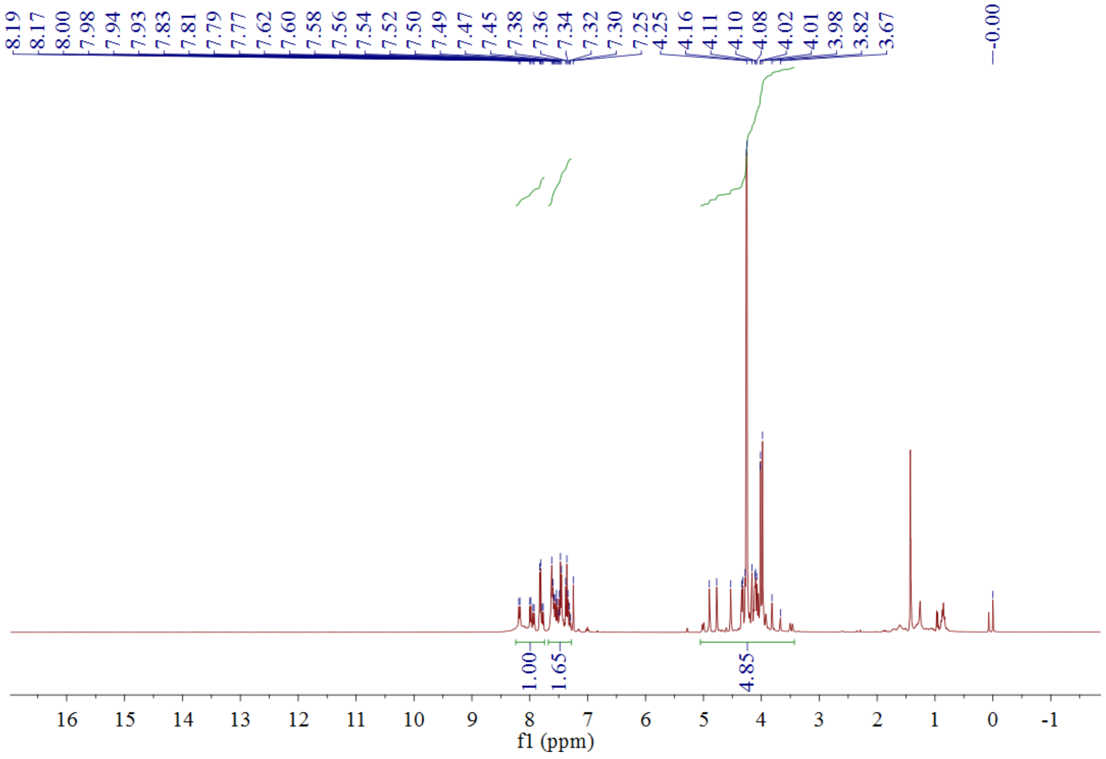

^{1}H NMR spectrum of compound **1c**.

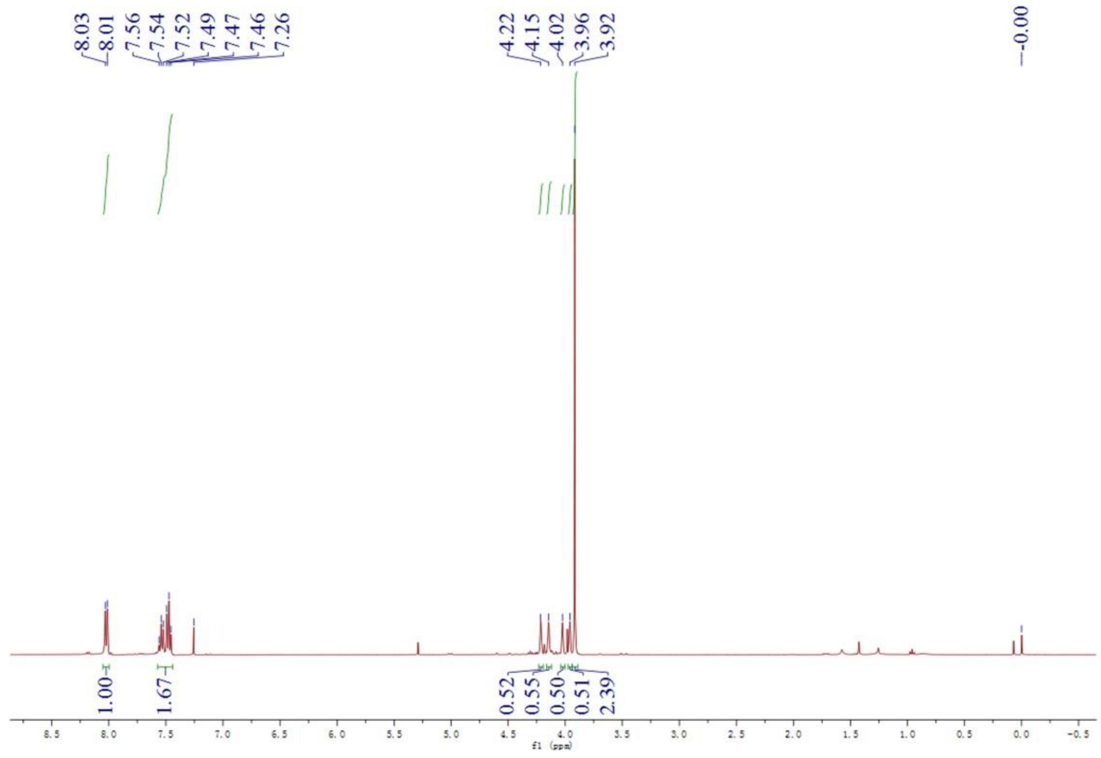

^{1}H NMR spectrum of compound **1d**.

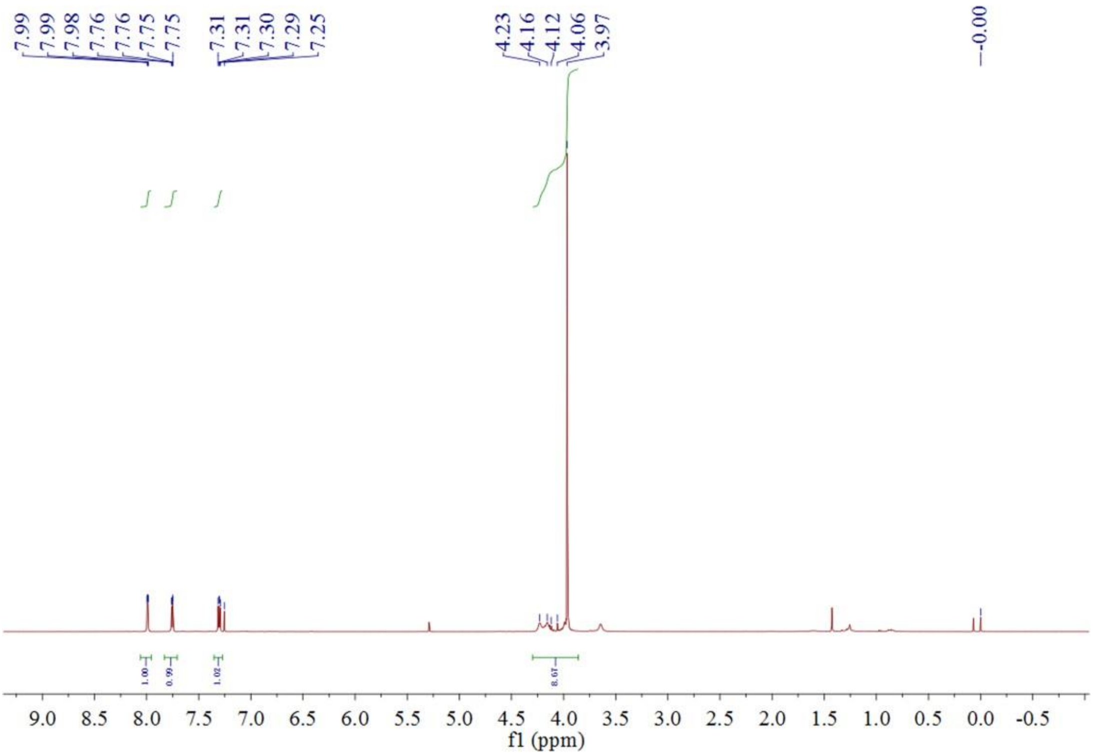

^{1}H NMR spectrum of compound **2a**.

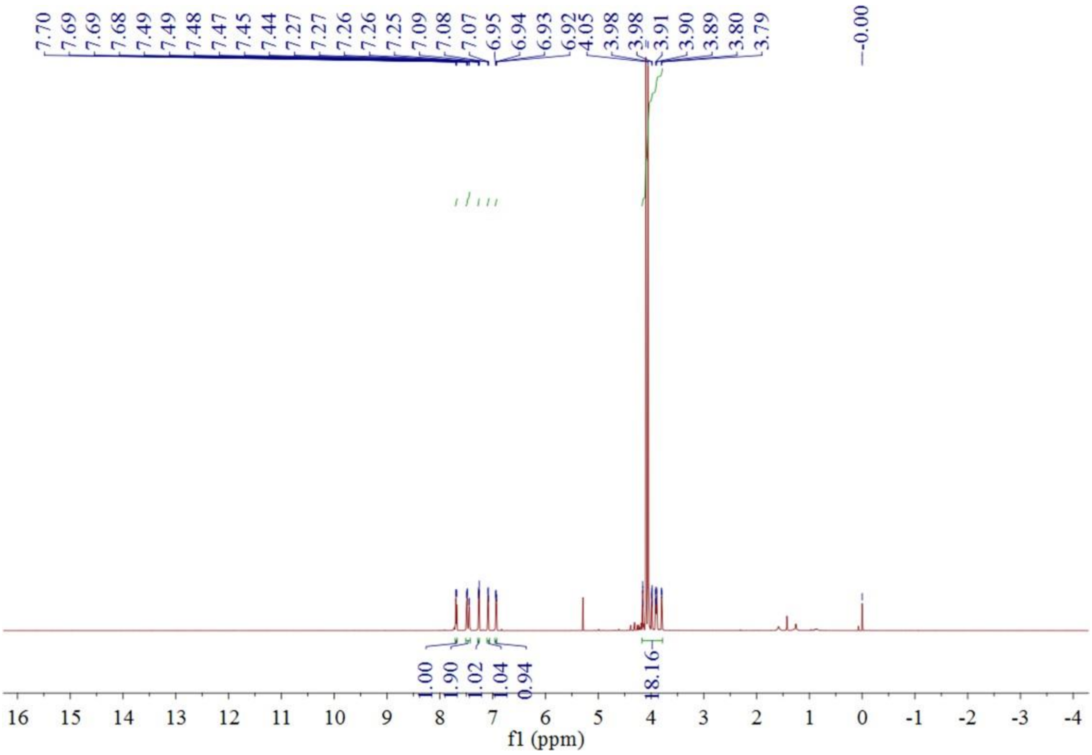

^{1}H NMR spectrum of compound **2b**.

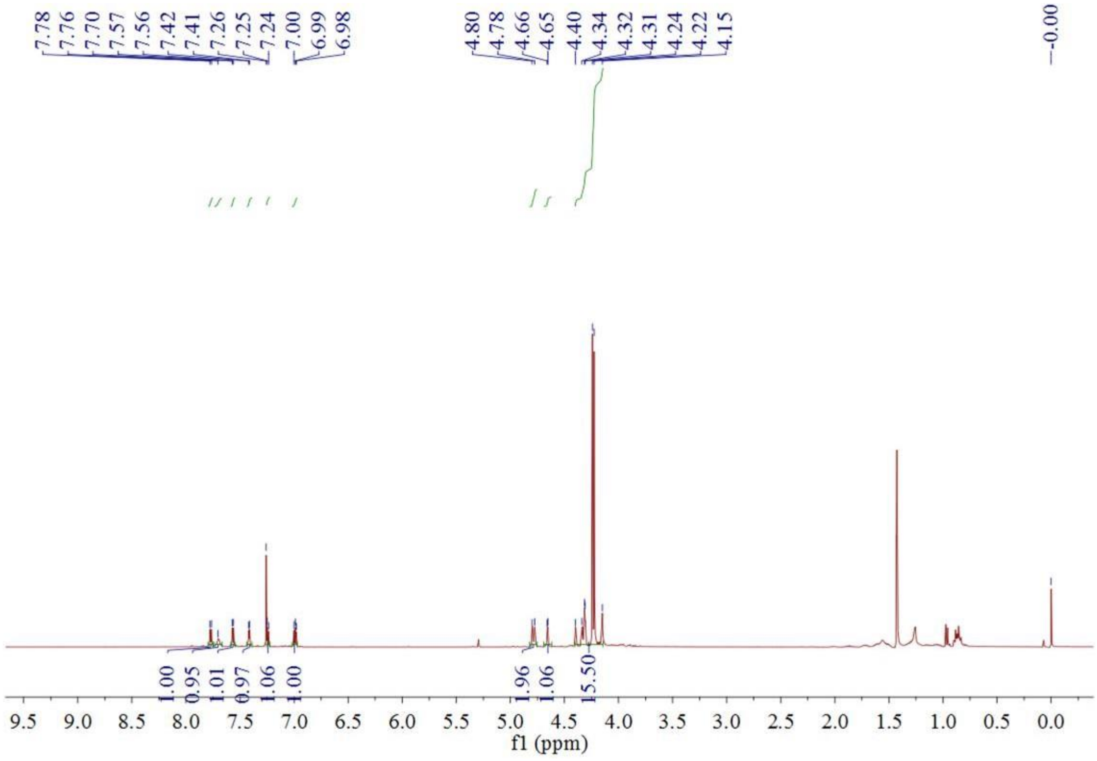

^{1}H NMR spectrum of compound **2c**.

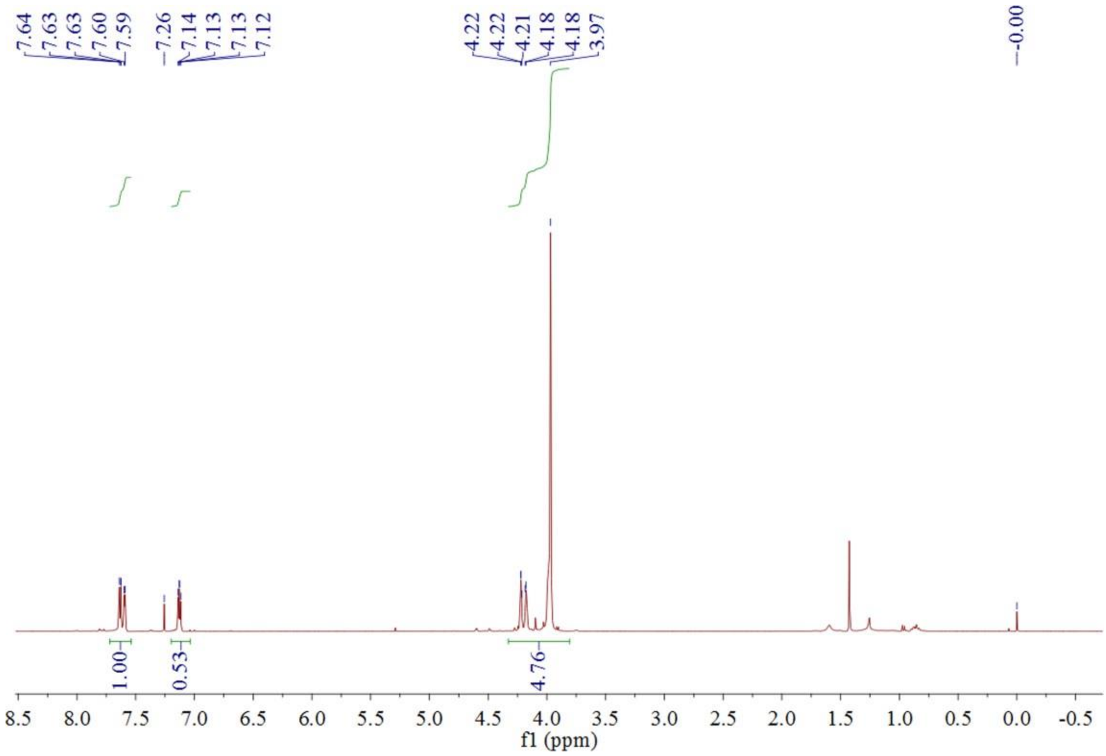

^{1}H NMR spectrum of compound **2d**.

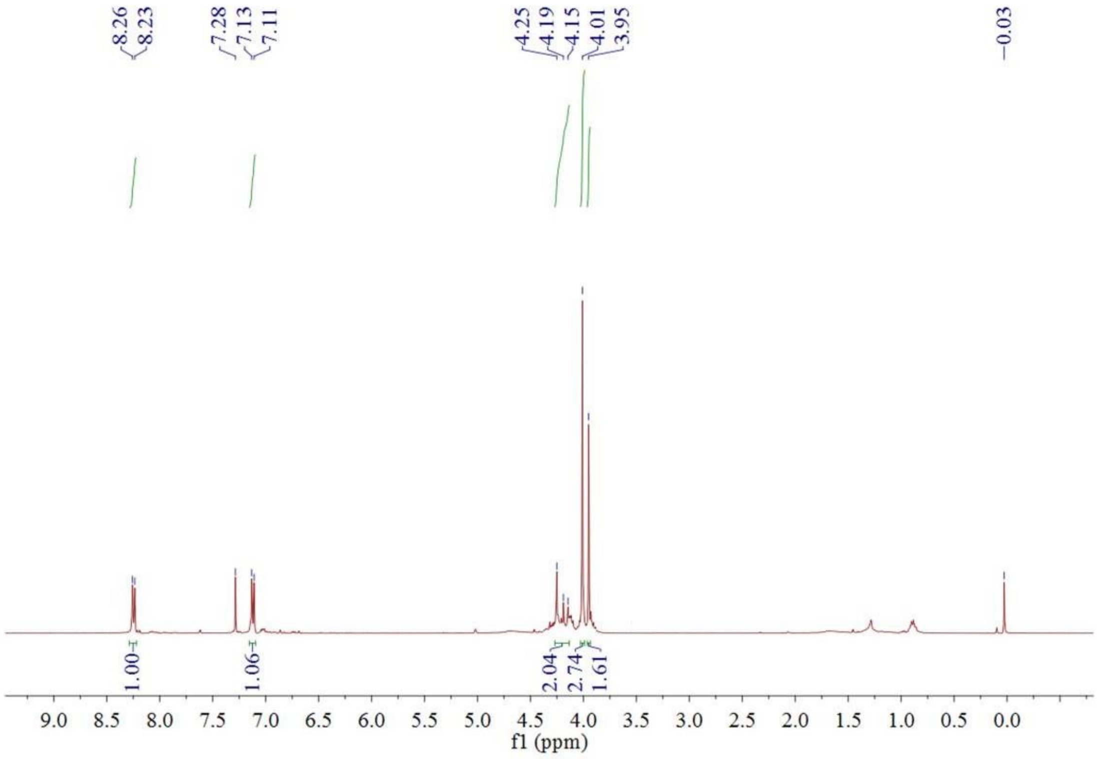

^{1}H NMR spectrum of compound **3a**.

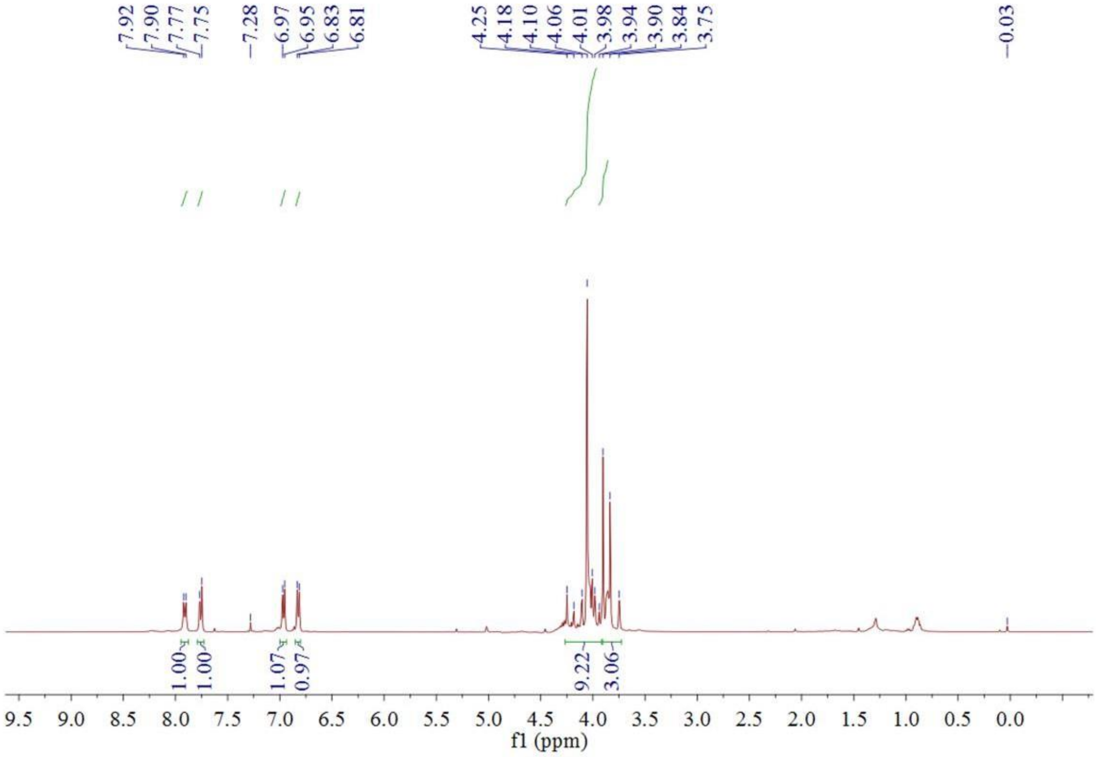

^{1}H NMR spectrum of compound **3b**.

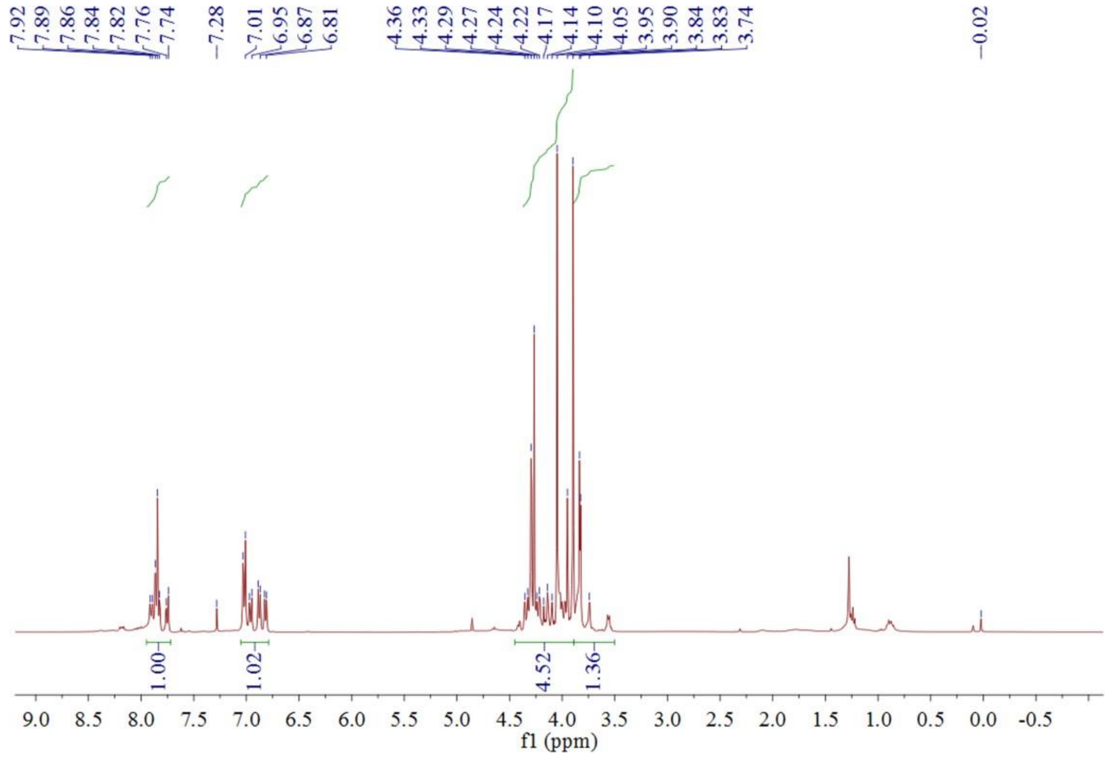

^{1}H NMR spectrum of compound **3c**.

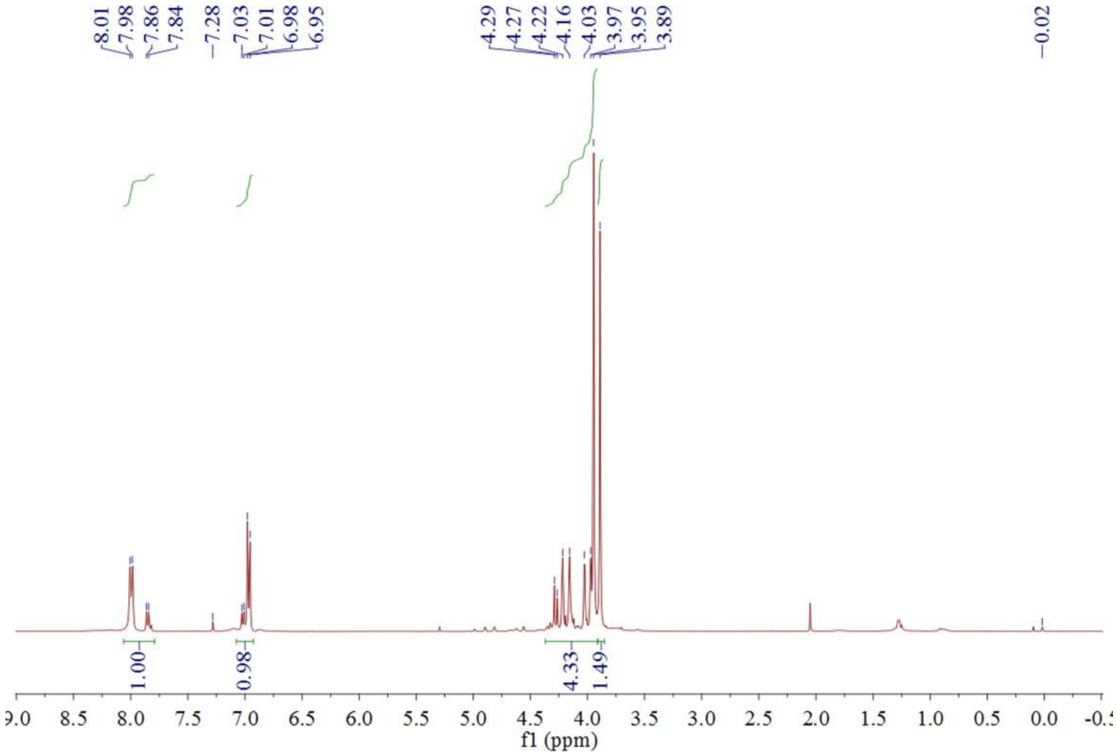

^{1}H NMR spectrum of compound **3d**.

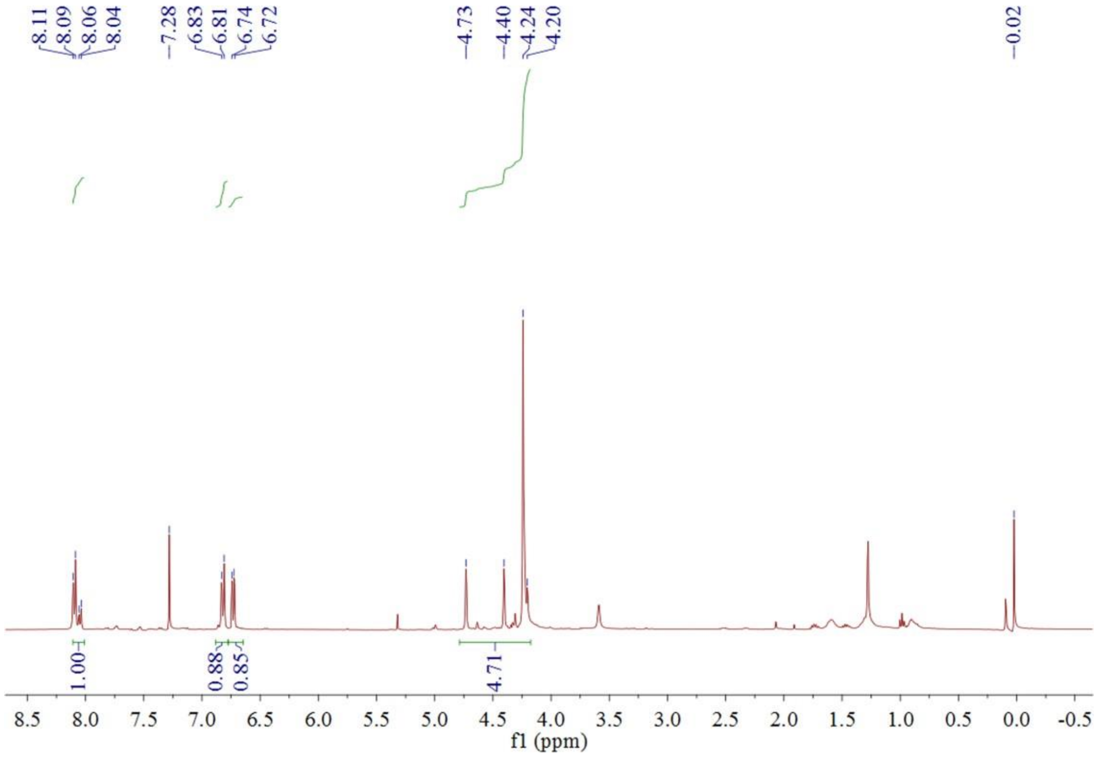

^{1}H NMR spectrum of compound **4a**.

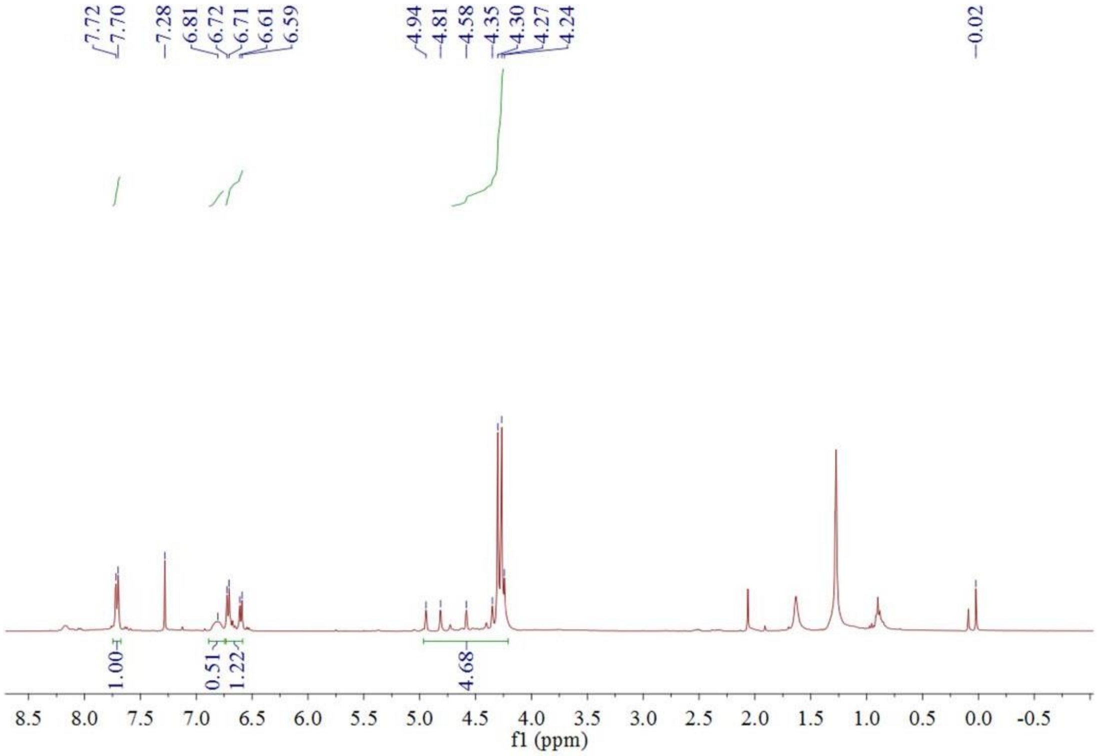

^{1}H NMR spectrum of compound **4b**.

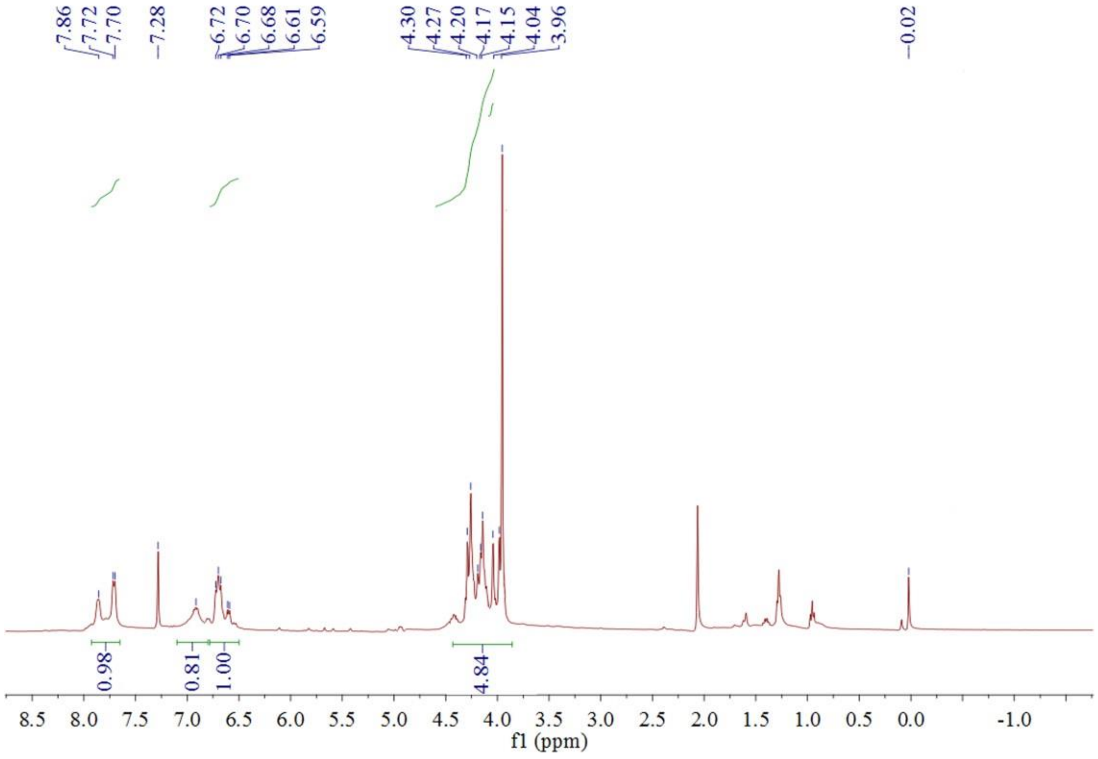

^{1}H NMR spectrum of compound **4c**.

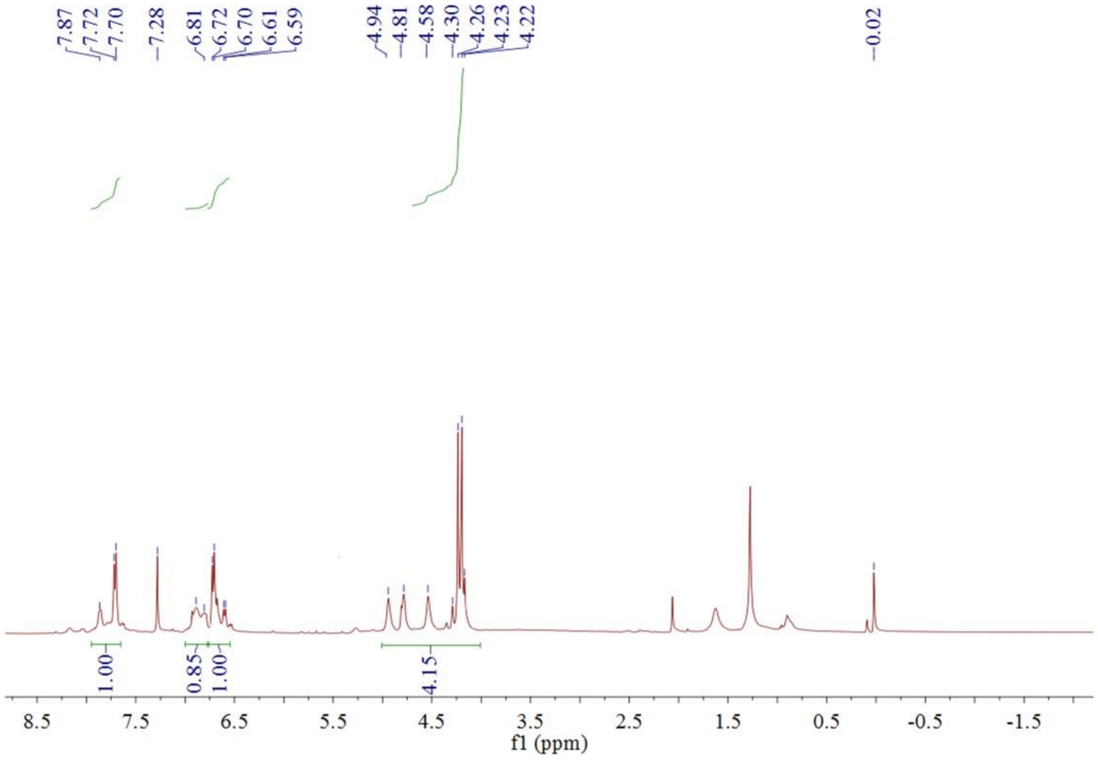

^{1}H NMR spectrum of compound **4d**.

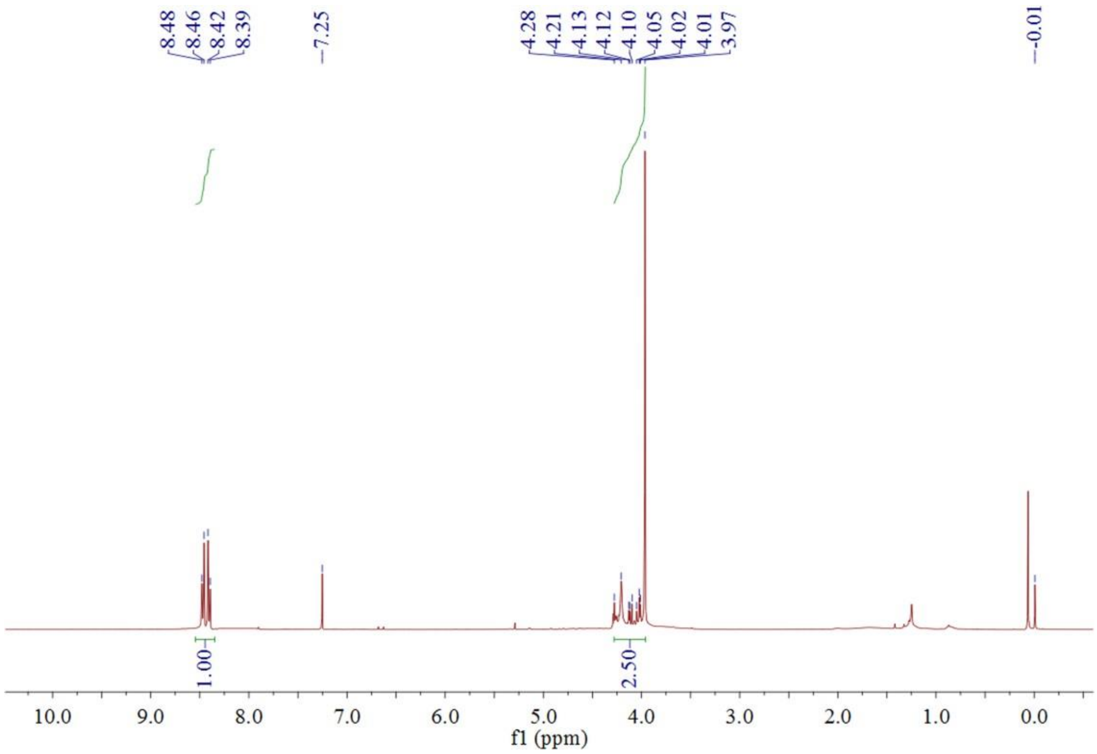

^{1}H NMR spectrum of compound **5a**.

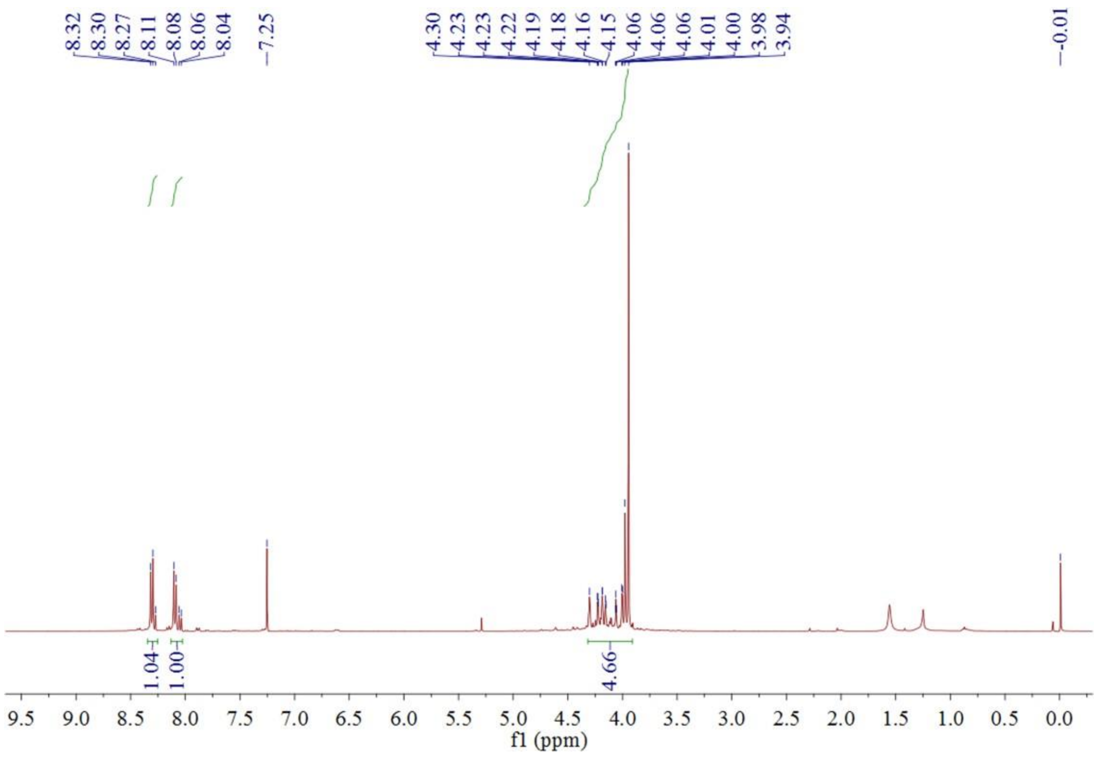

^{1}H NMR spectrum of compound **5b**.

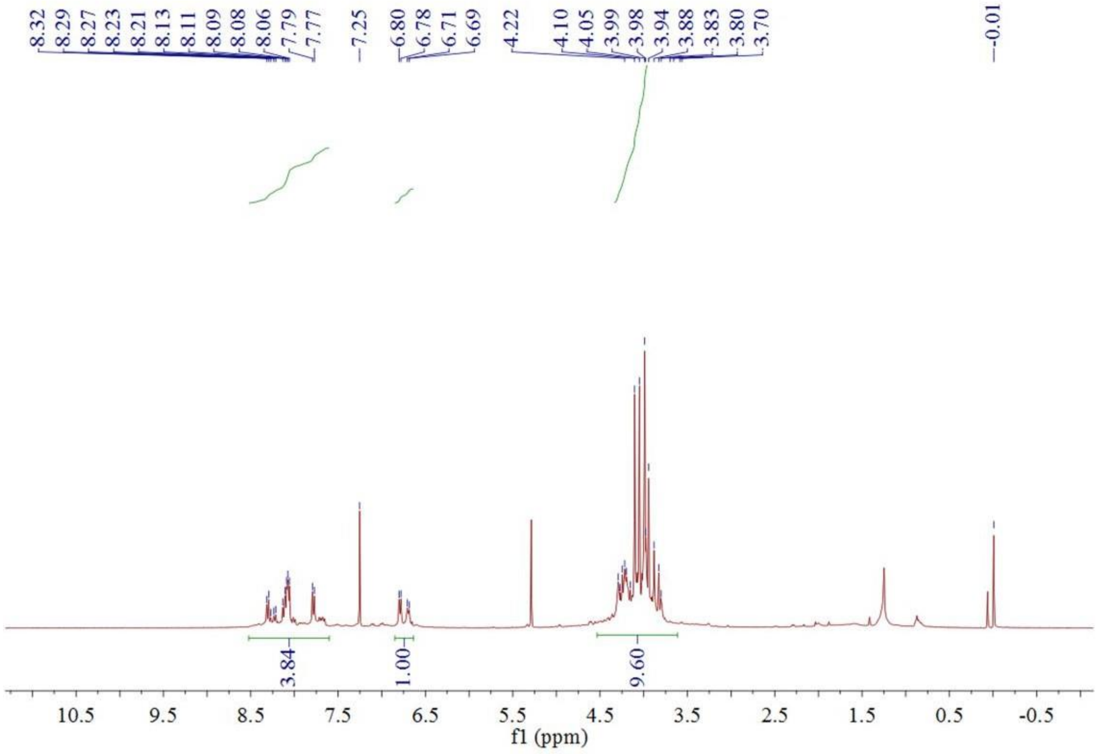

^{1}H NMR spectrum of compound **5c**.

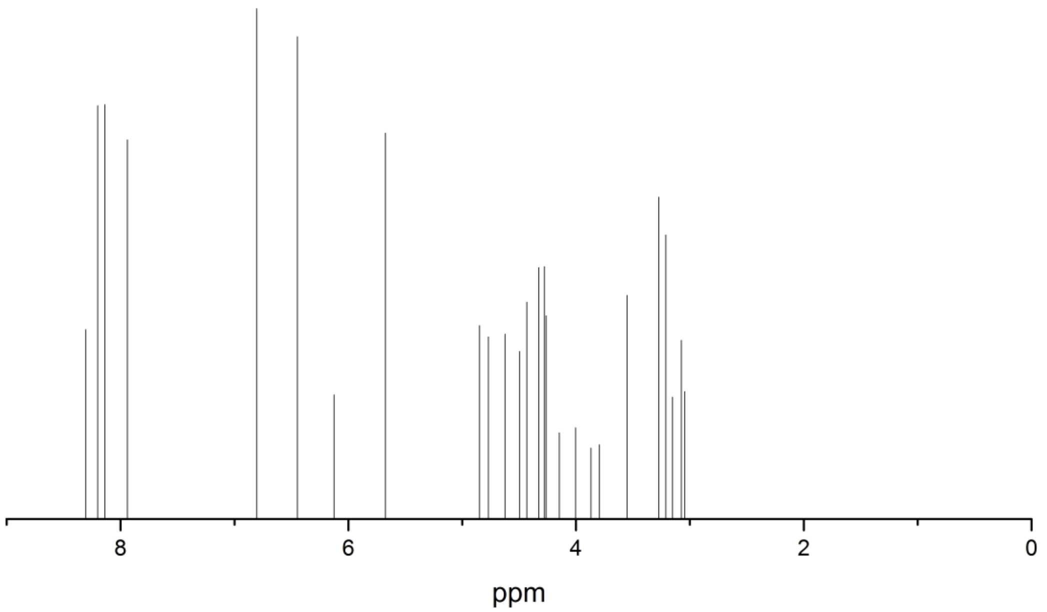

Calculated ^{1}H NMR spectrum of compound **5c**.

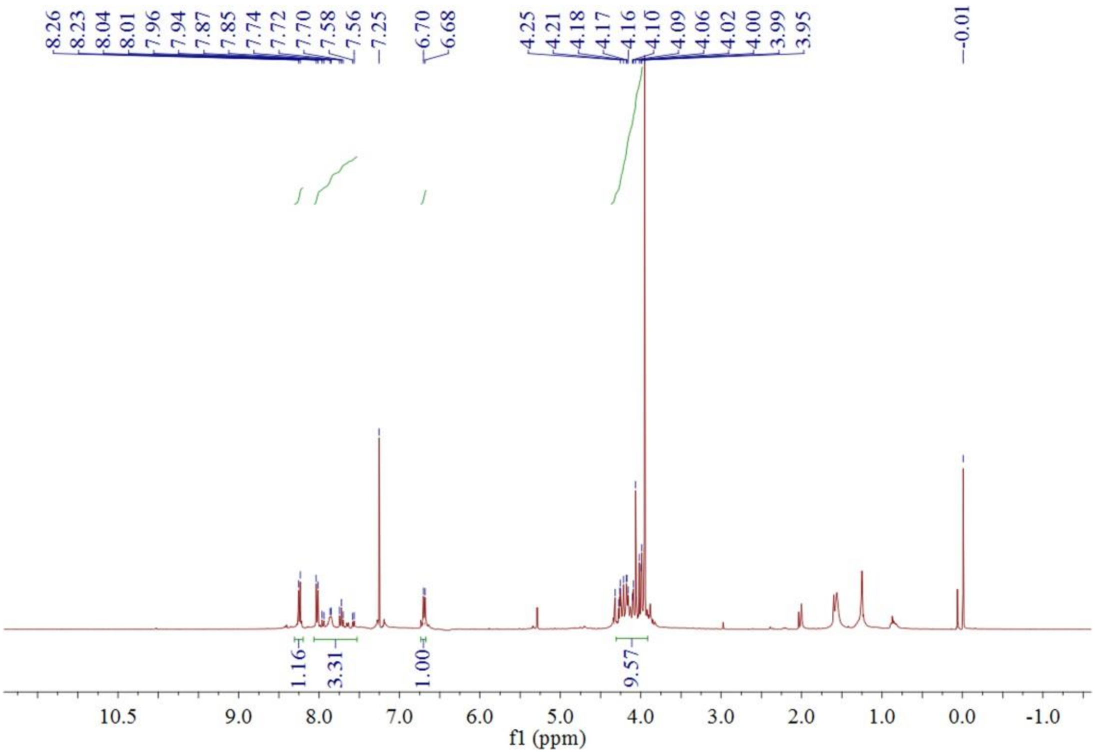

^{1}H NMR spectrum of compound **5d**.

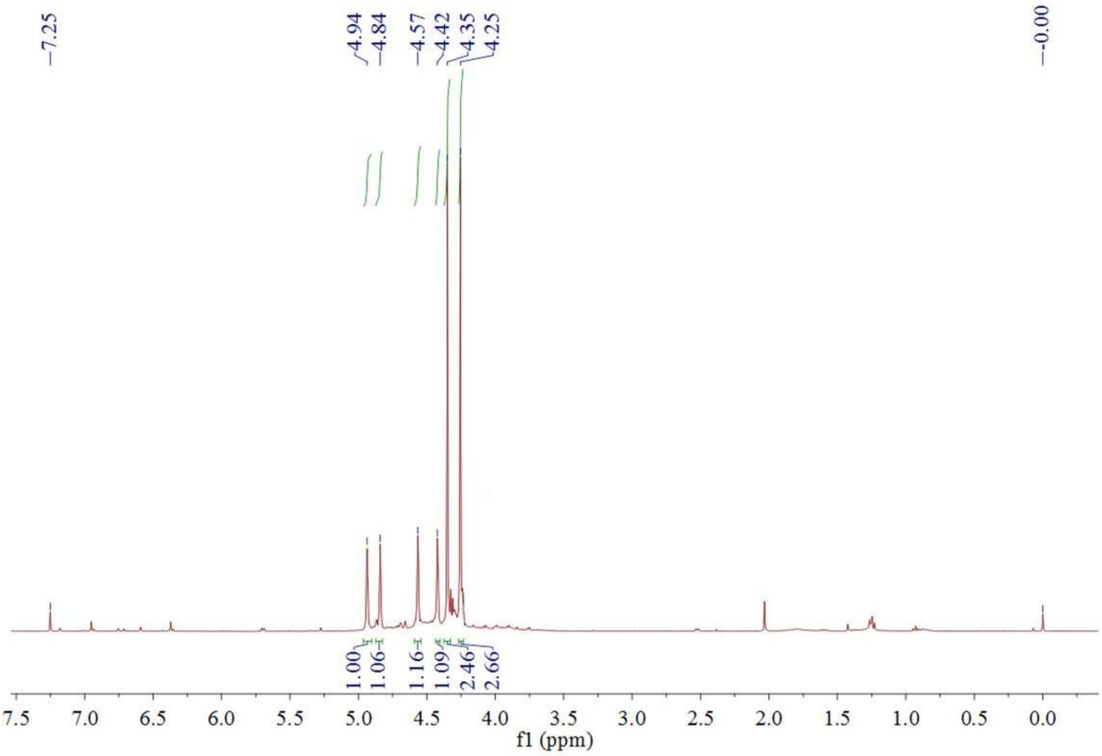

^{1}H NMR spectrum of compound **6a**.

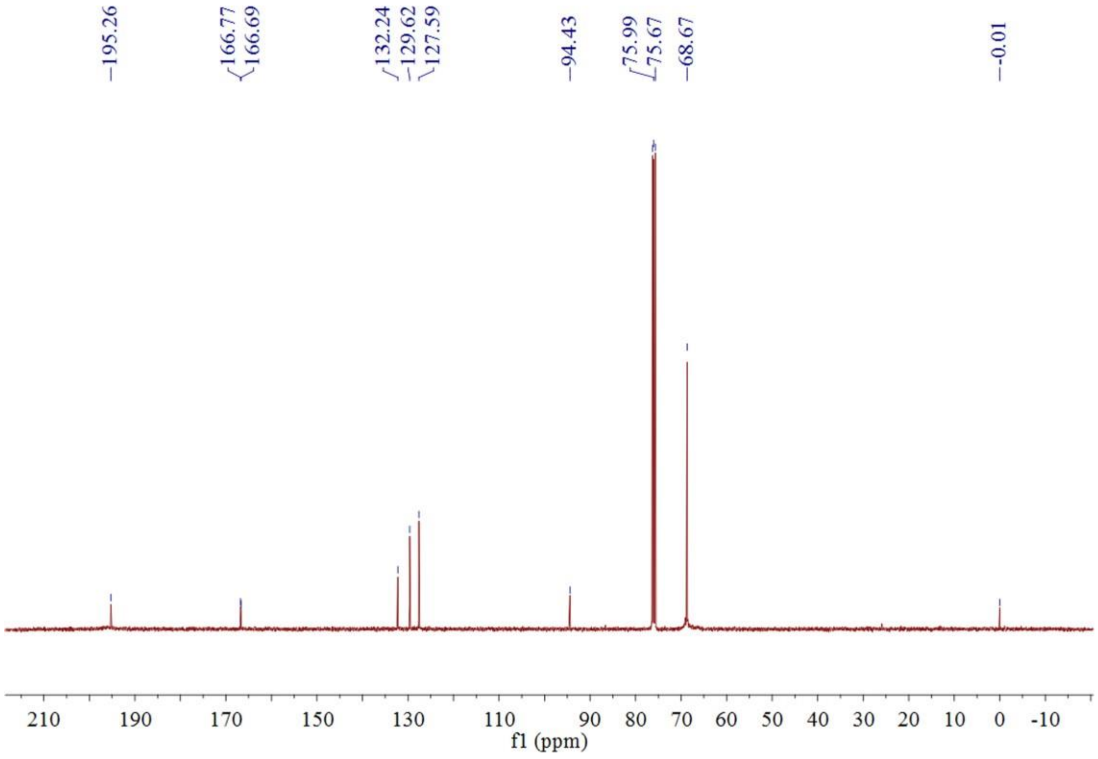

$^{13}C\{1H\}$ NMR spectrum of compound **1a**.

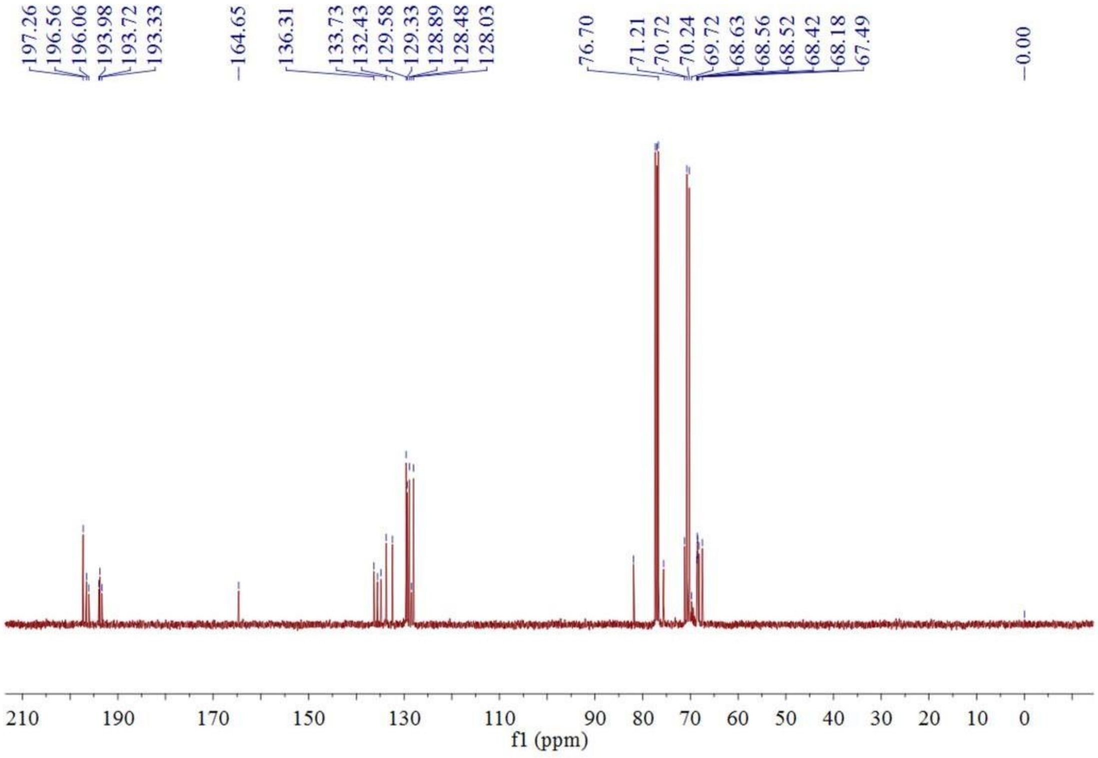

$^{13}C\{1H\}$ NMR spectrum of compound **1b**.

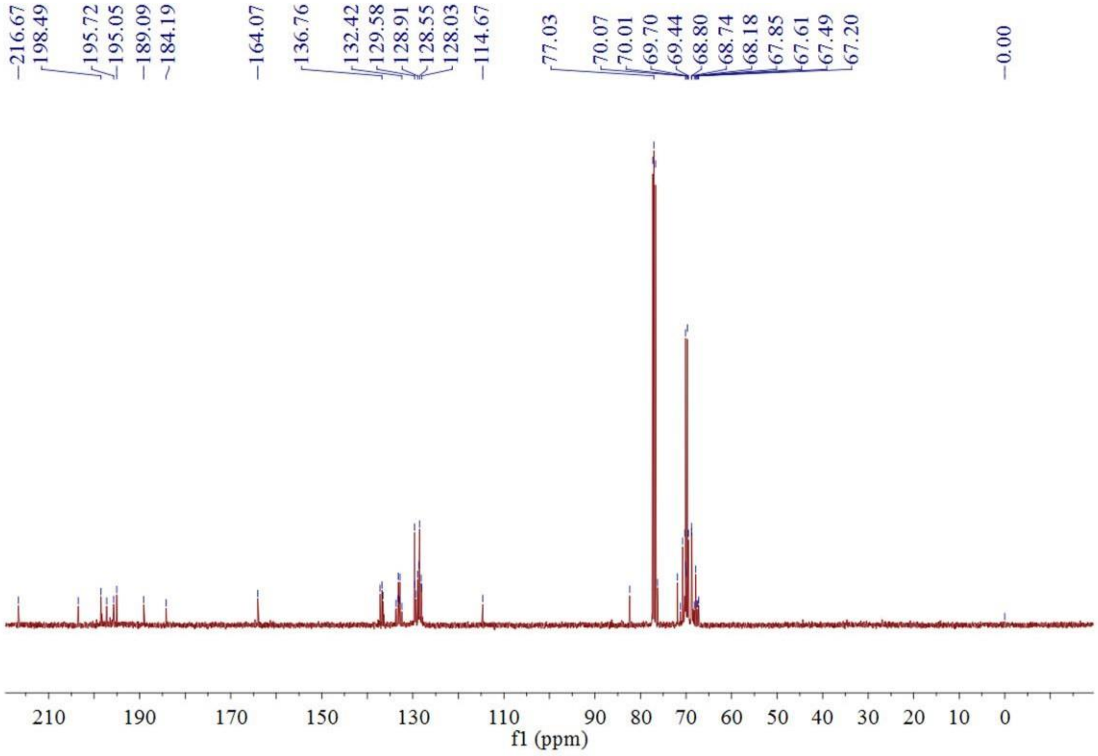

^{13}C{1H} NMR spectrum of compound **1c**.

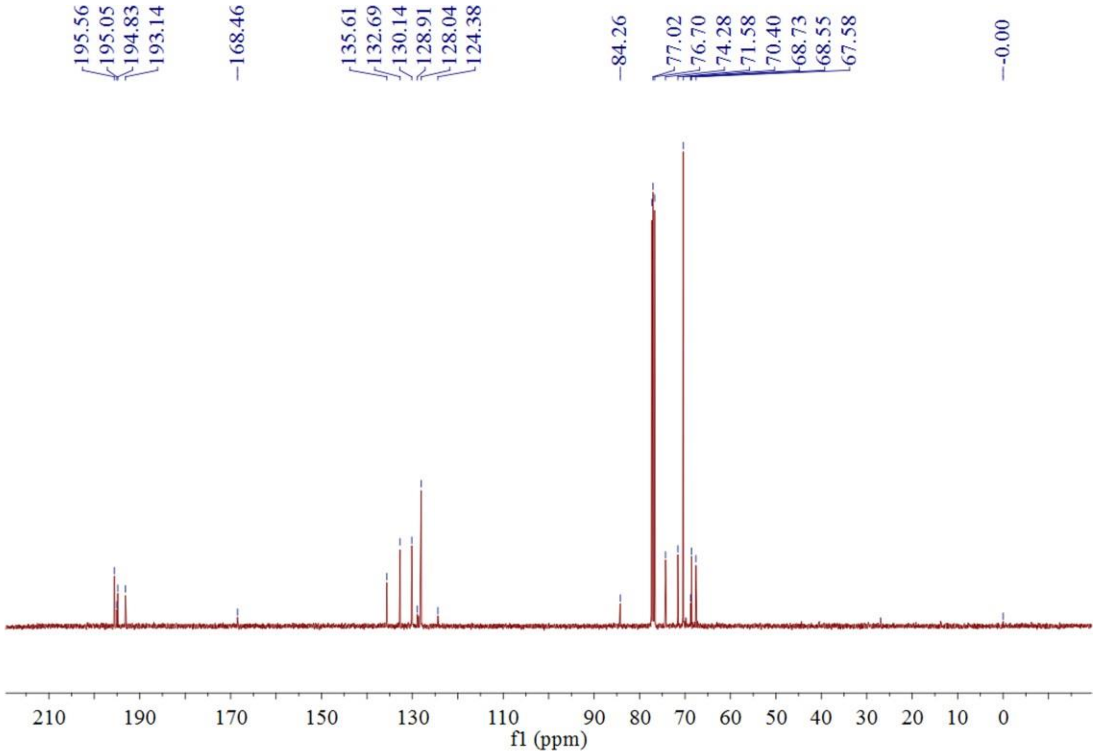

^{13}C{1H} NMR spectrum of compound **1d**.

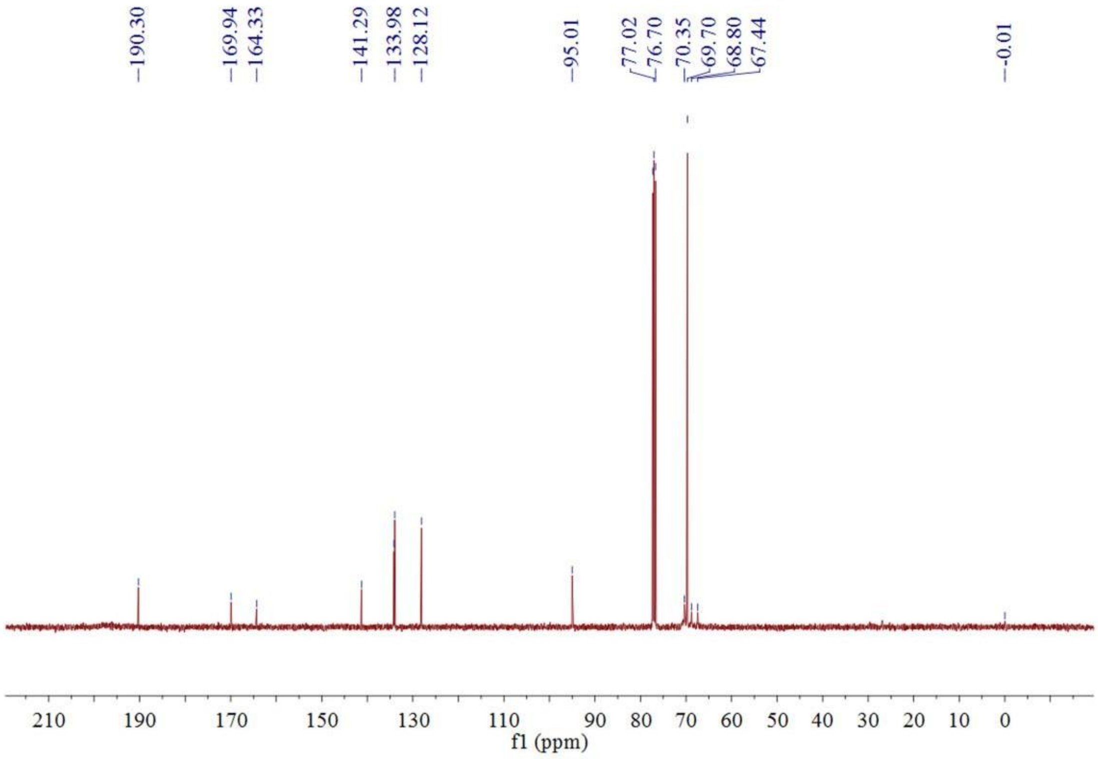

$^{13}C\{1H\}$ NMR spectrum of compound **2a**.

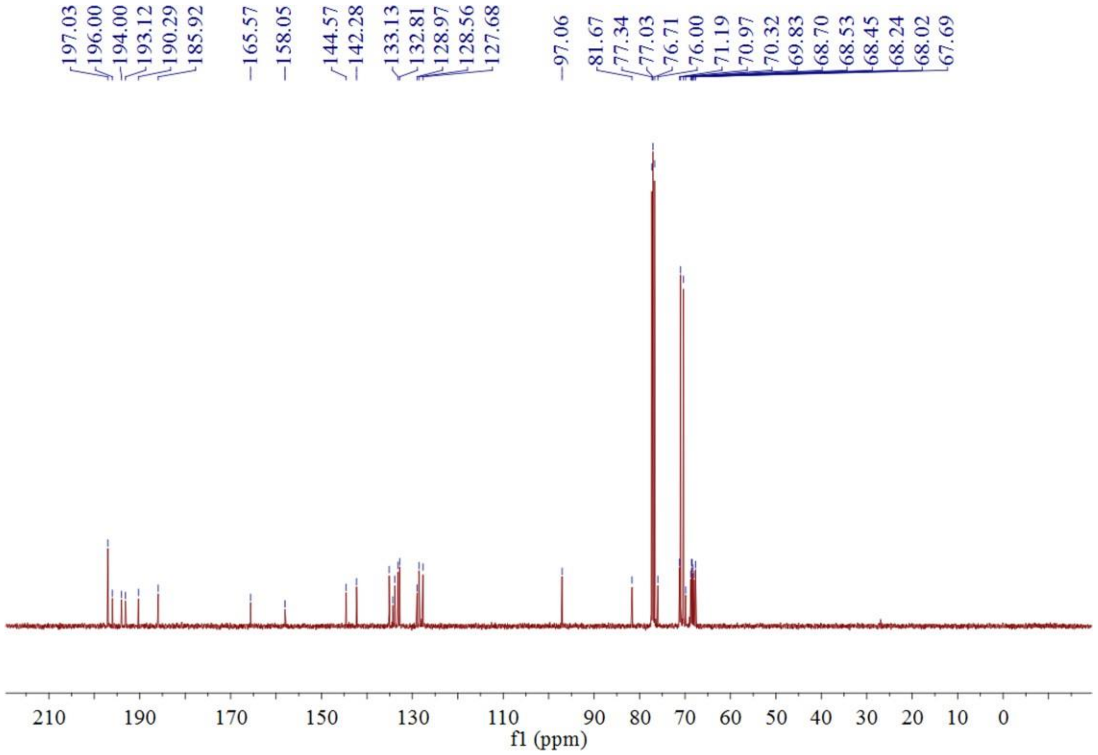

$^{13}C\{1H\}$ NMR spectrum of compound **2b**.

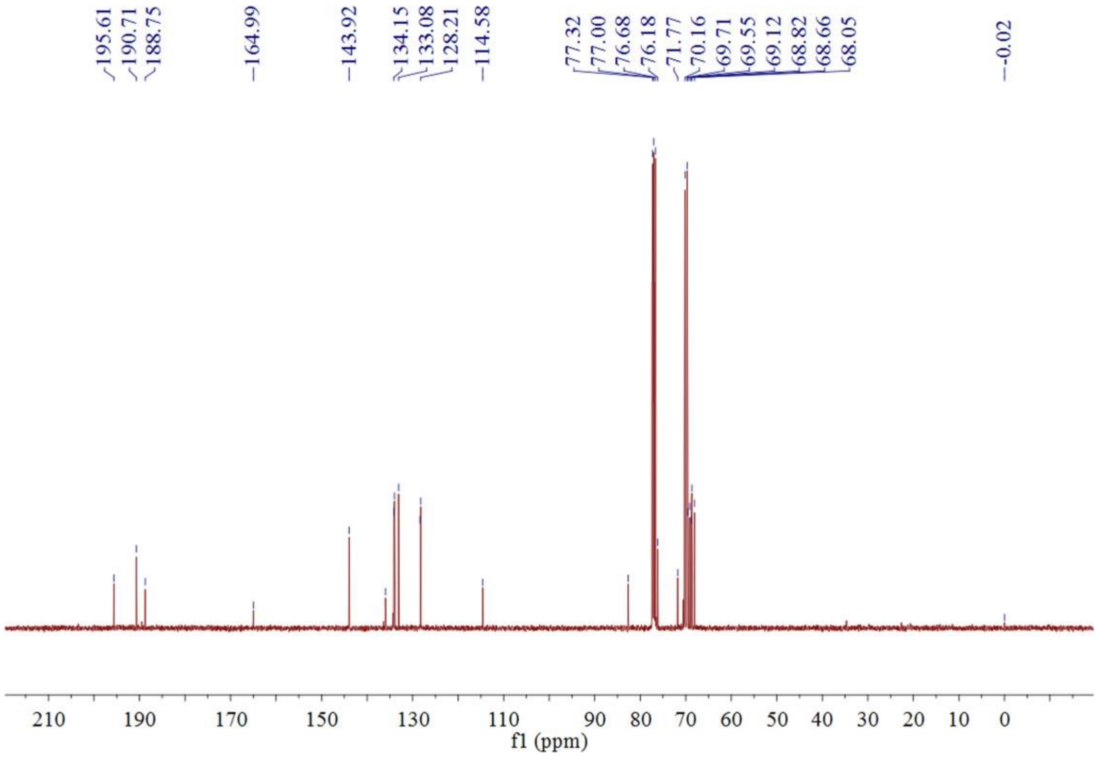

^{13}C{1H} NMR spectrum of compound **2c**.

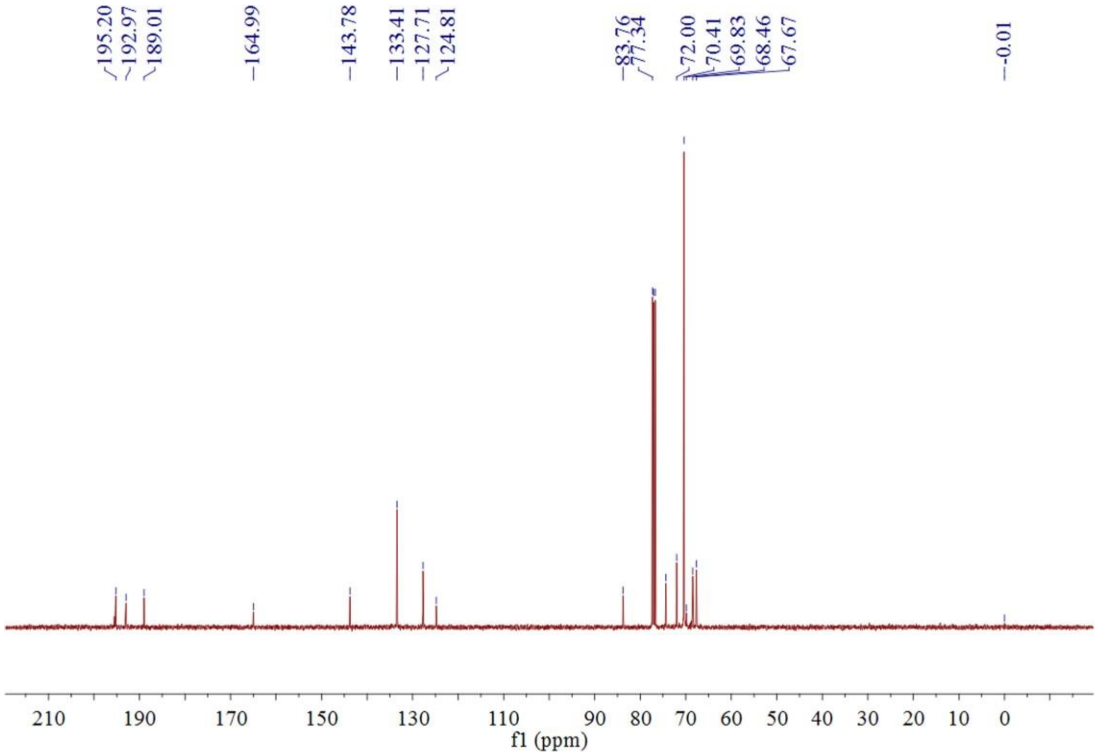

^{13}C{1H} NMR spectrum of compound **2d**.

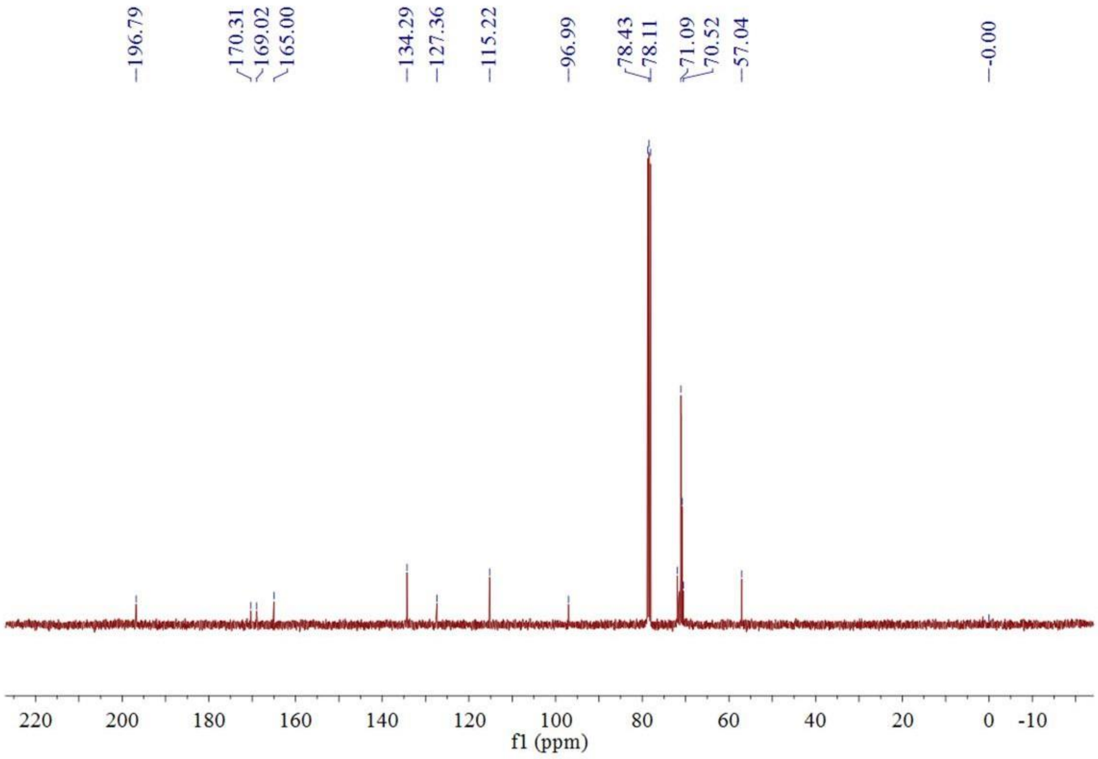

$^{13}C\{1H\}$ NMR spectrum of compound **3a**.

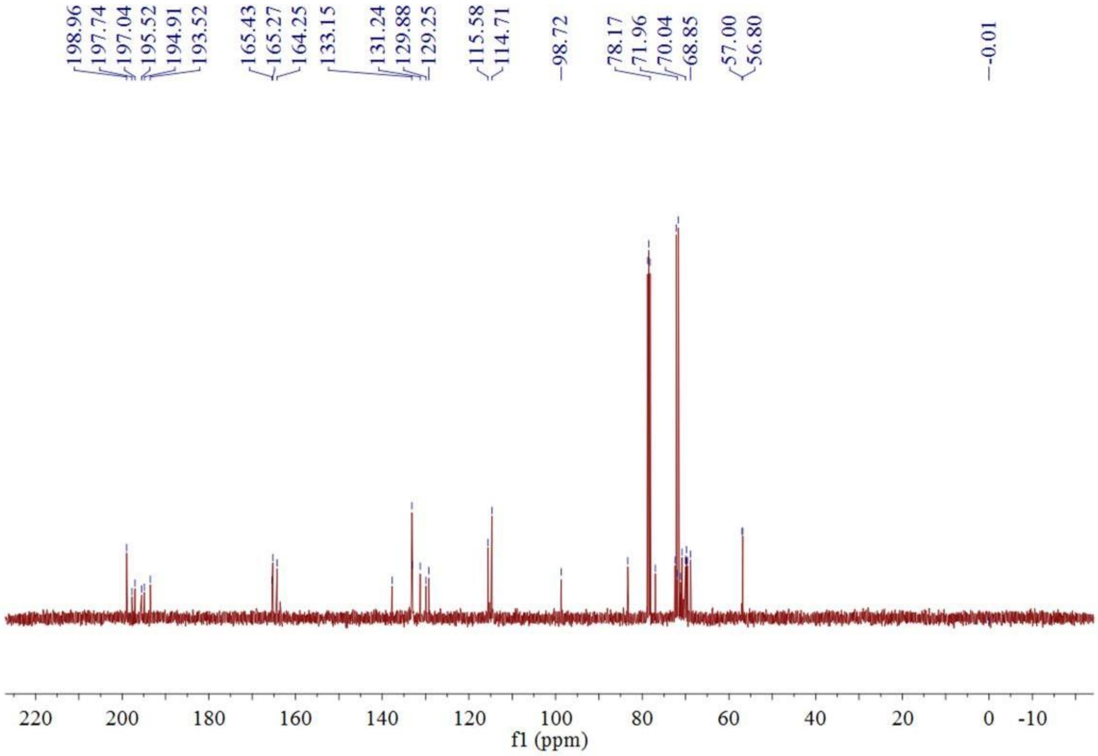

$^{13}C\{1H\}$ NMR spectrum of compound **3b**.

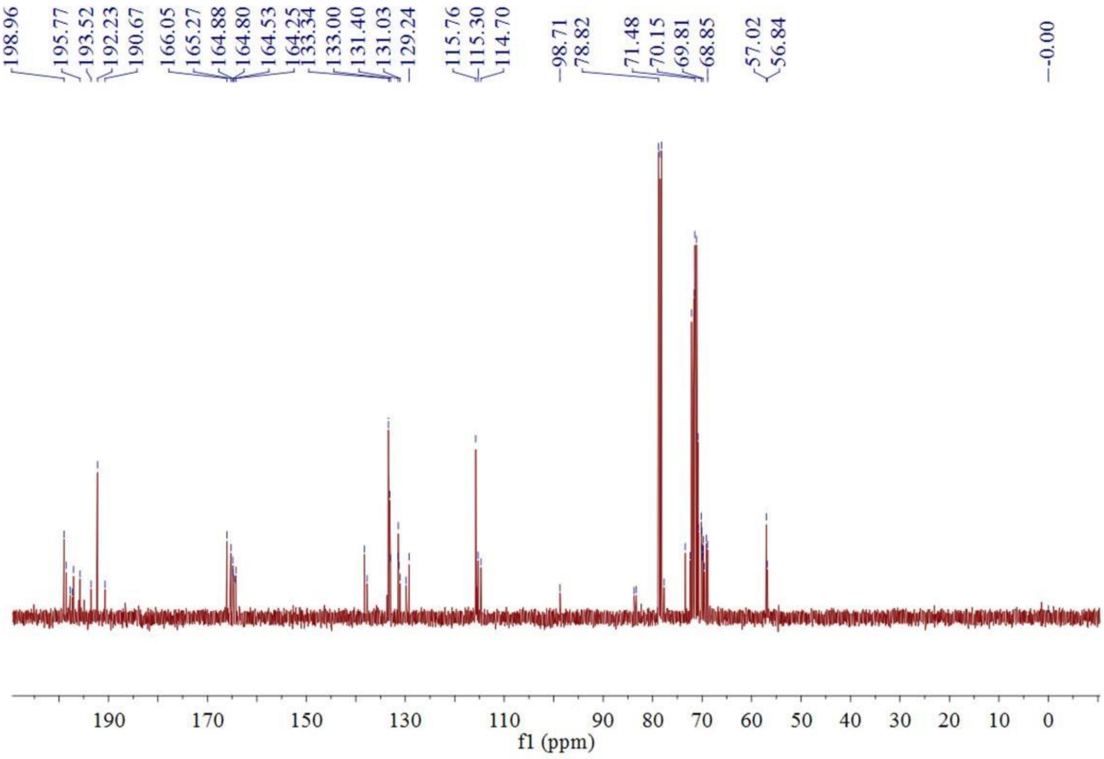

^{13}C{1H} NMR spectrum of compound **3c**.

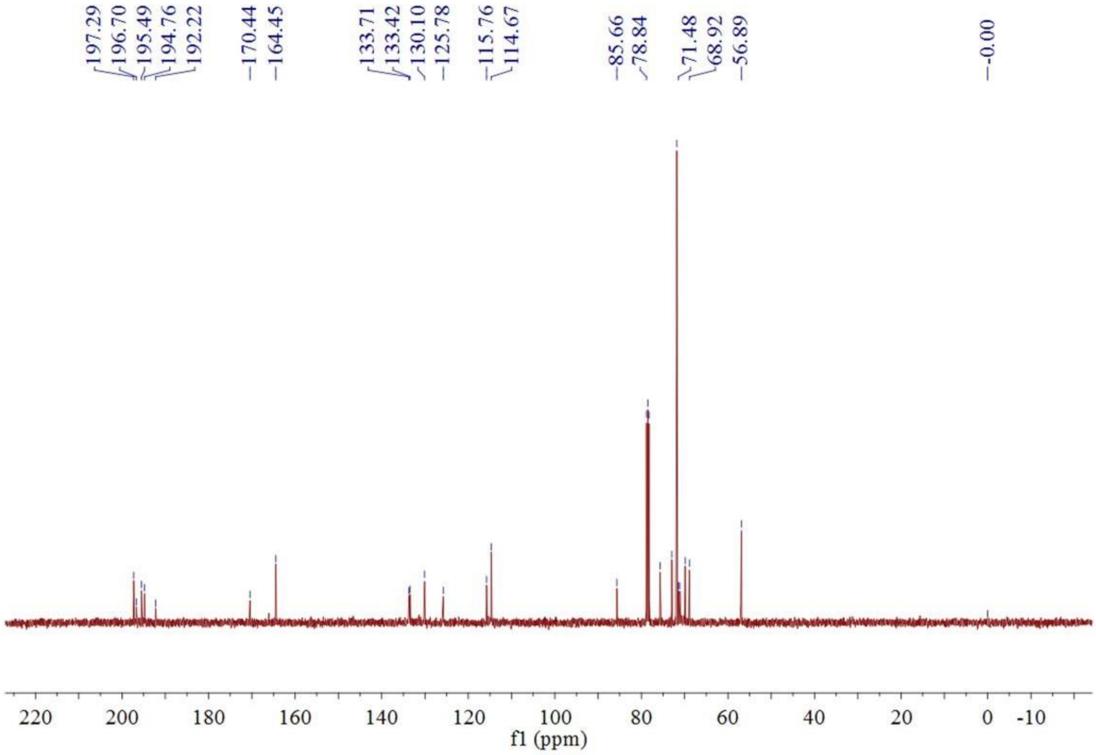

^{13}C{1H} NMR spectrum of compound **3d**.

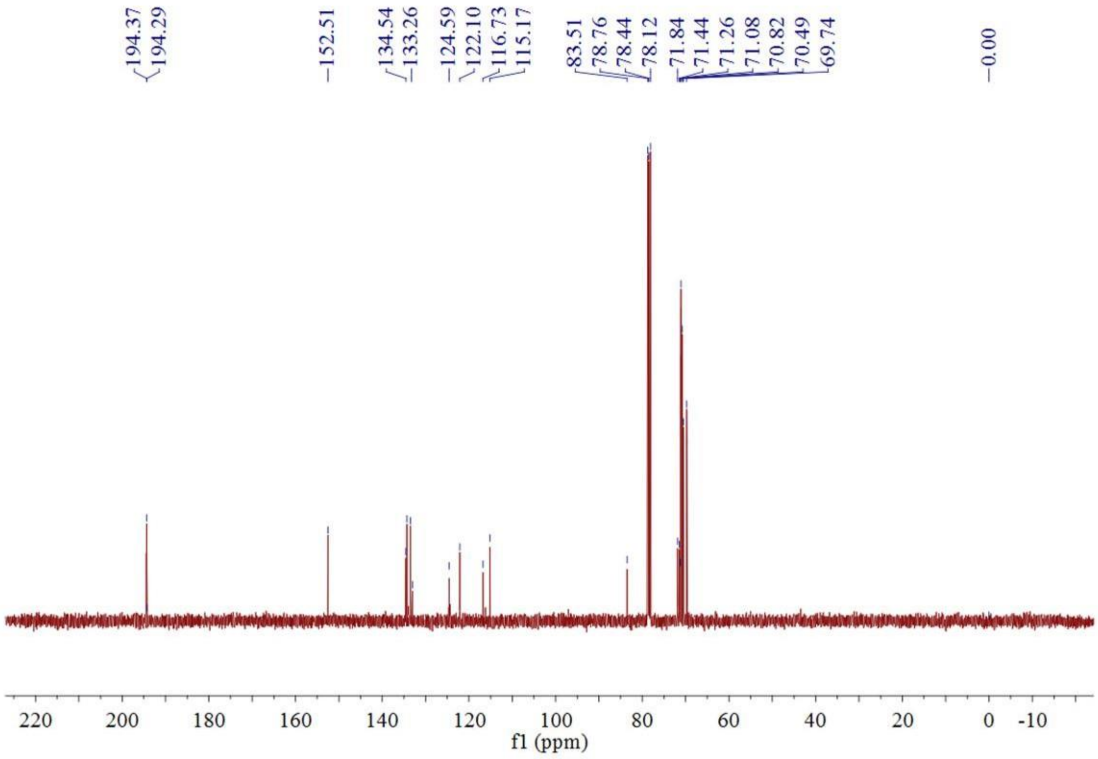

$^{13}C\{1H\}$ NMR spectrum of compound **4a**.

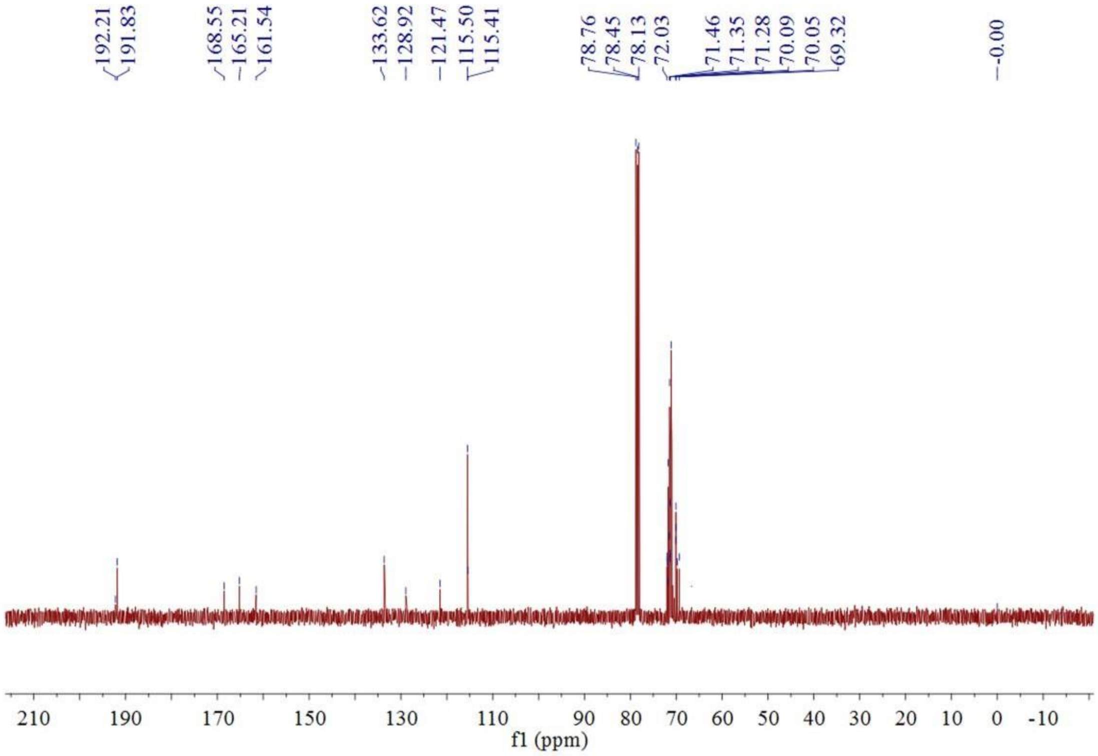

$^{13}C\{1H\}$ NMR spectrum of compound **4b**.

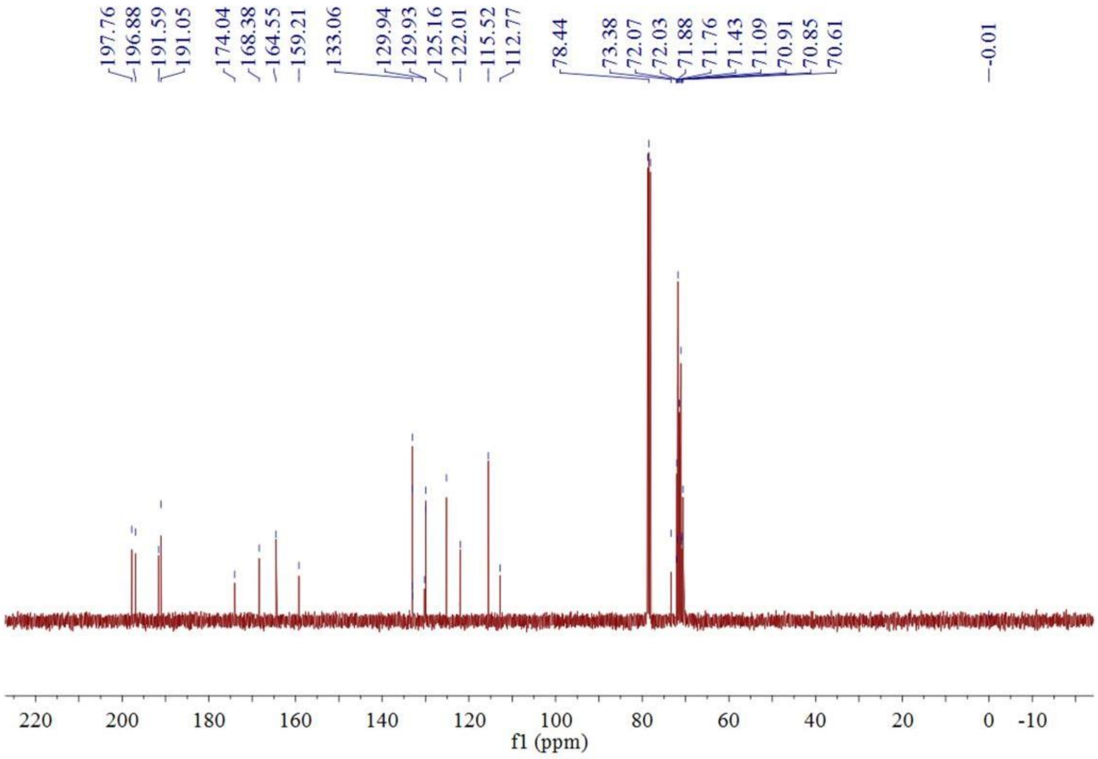

^{13}C{1H} NMR spectrum of compound **4c**.

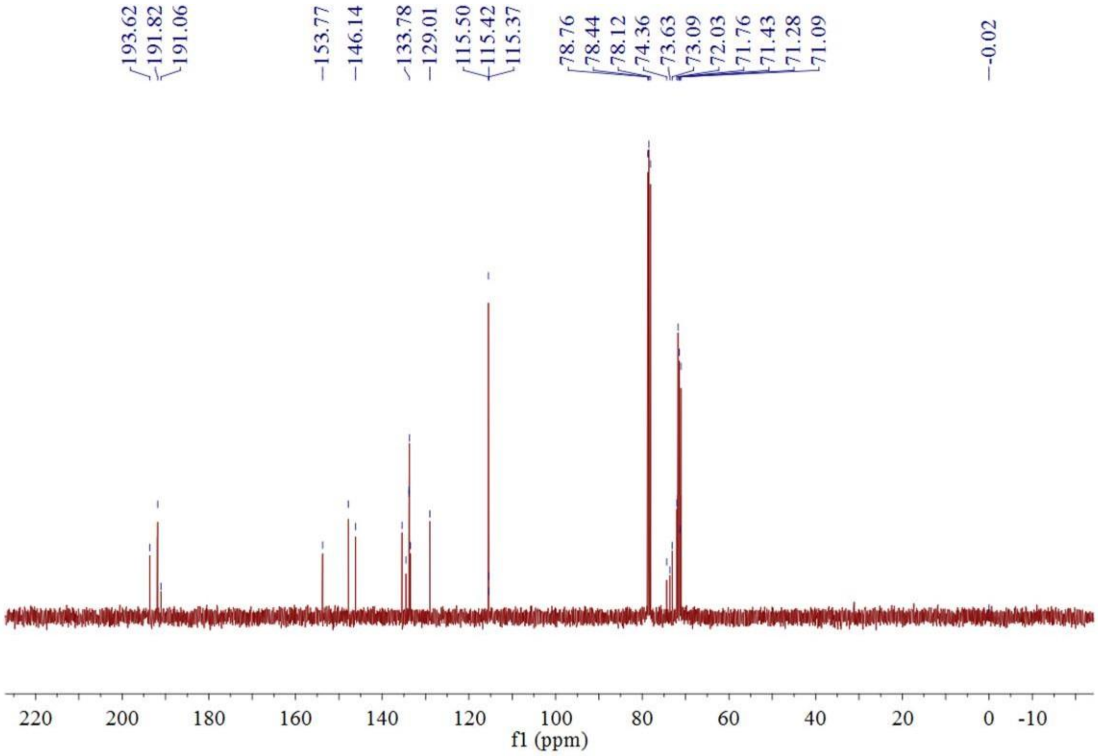

^{13}C{1H} NMR spectrum of compound **4d**.

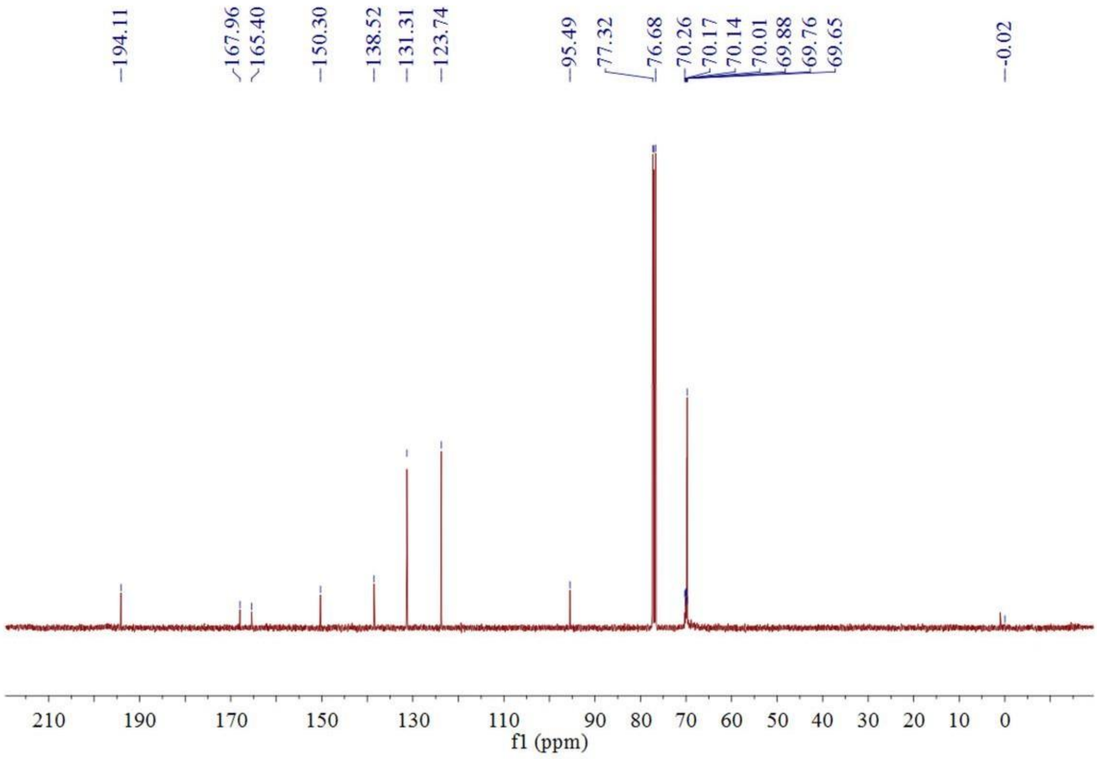

$^{13}C\{1H\}$ NMR spectrum of compound **5a**.

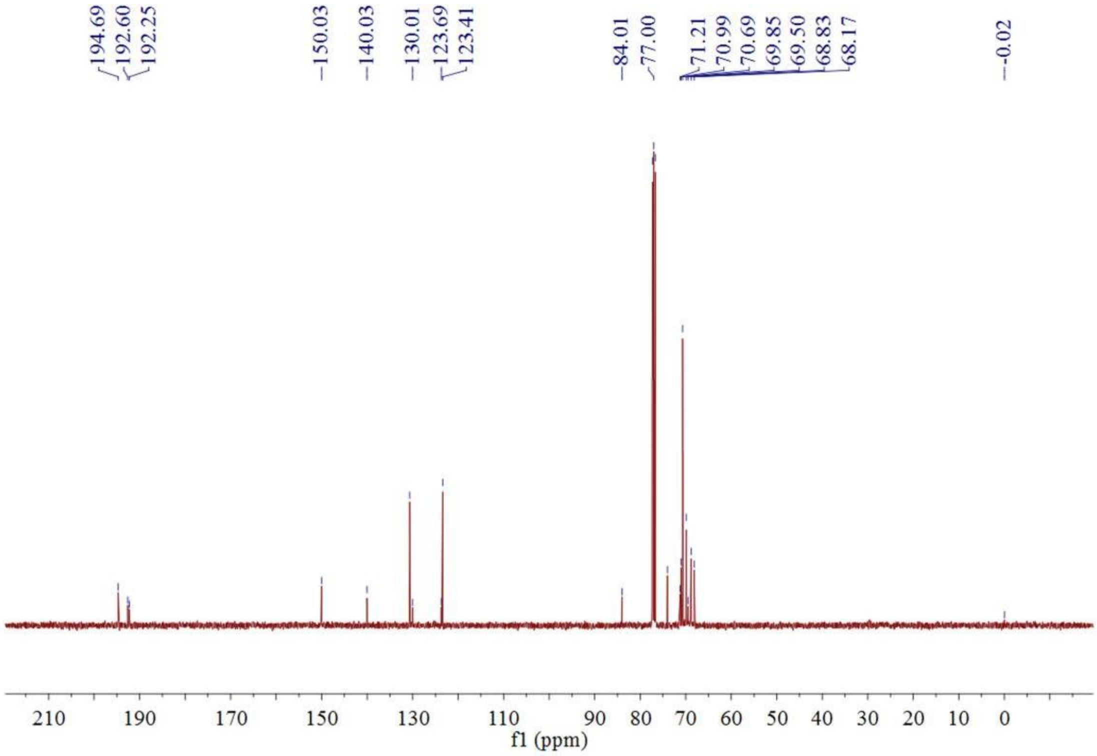

$^{13}C\{1H\}$ NMR spectrum of compound **5b**.

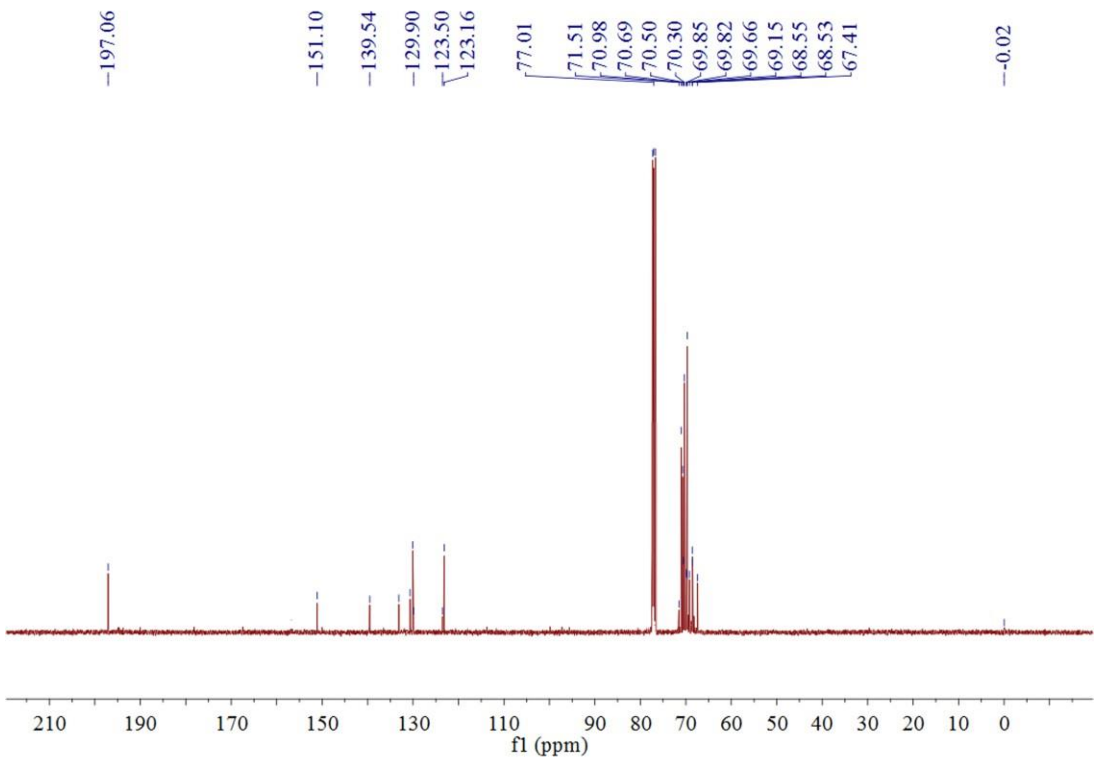

$^{13}C\{1H\}$ NMR spectrum of compound **5c**.

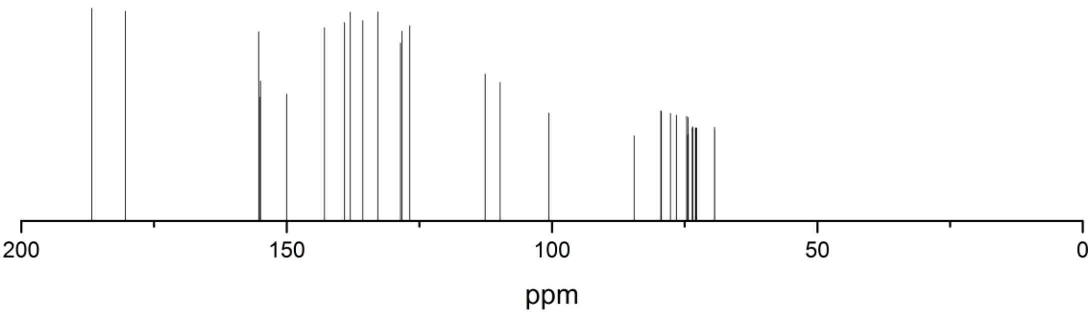

Calculated $^{13}C\{1H\}$ NMR spectrum of compound **5c**.

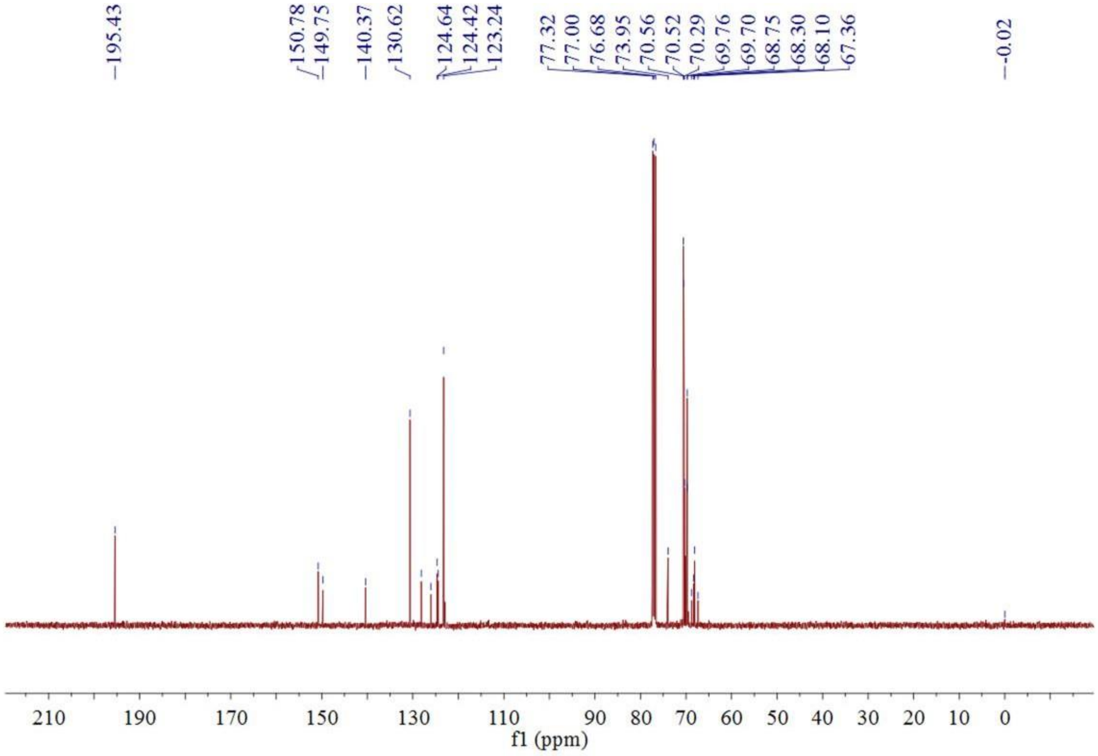

^{13}C{1H} NMR spectrum of compound **5d**.

+

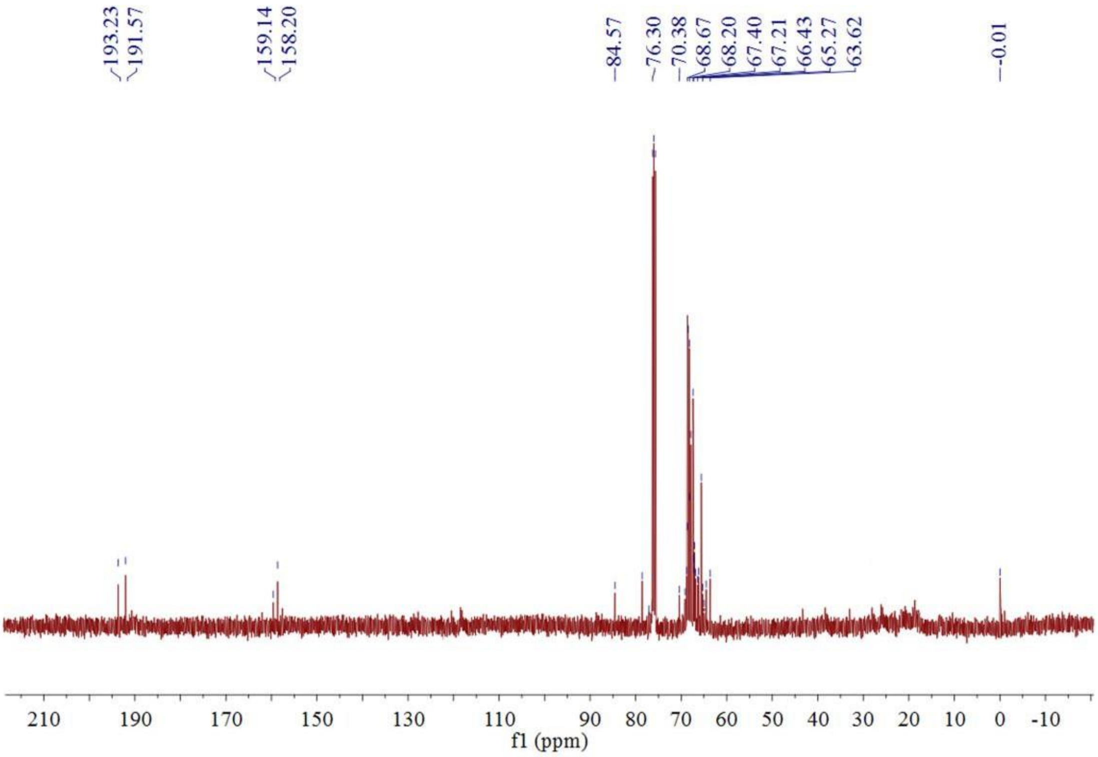

^{13}C{1H} NMR spectrum of compound **6a**.

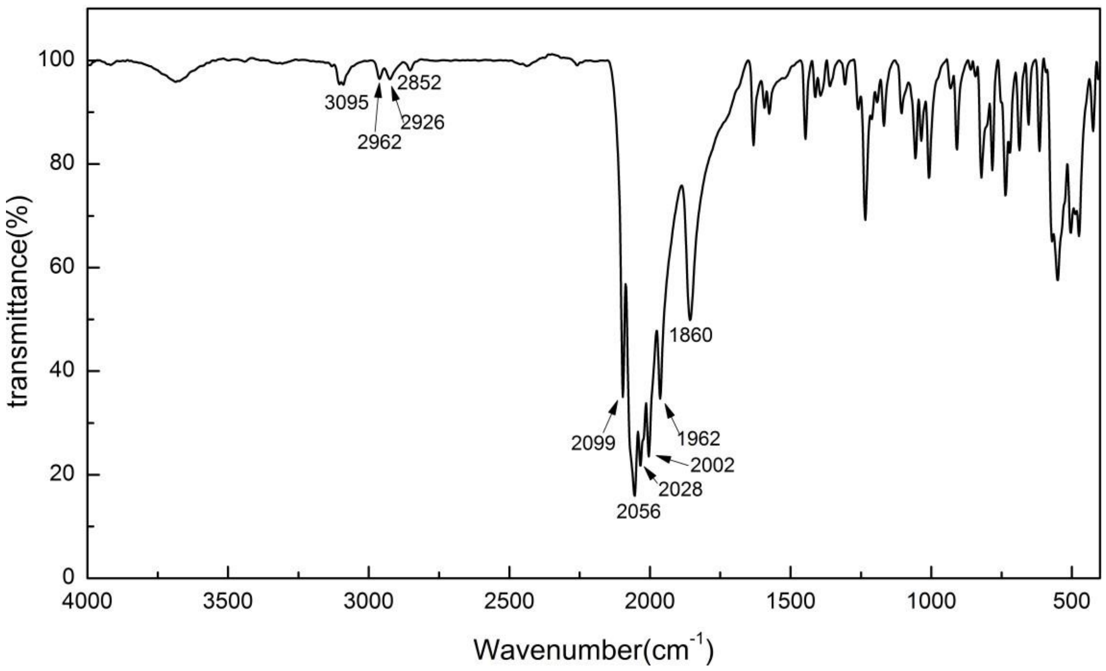

IR spectrum of compound **1a**.

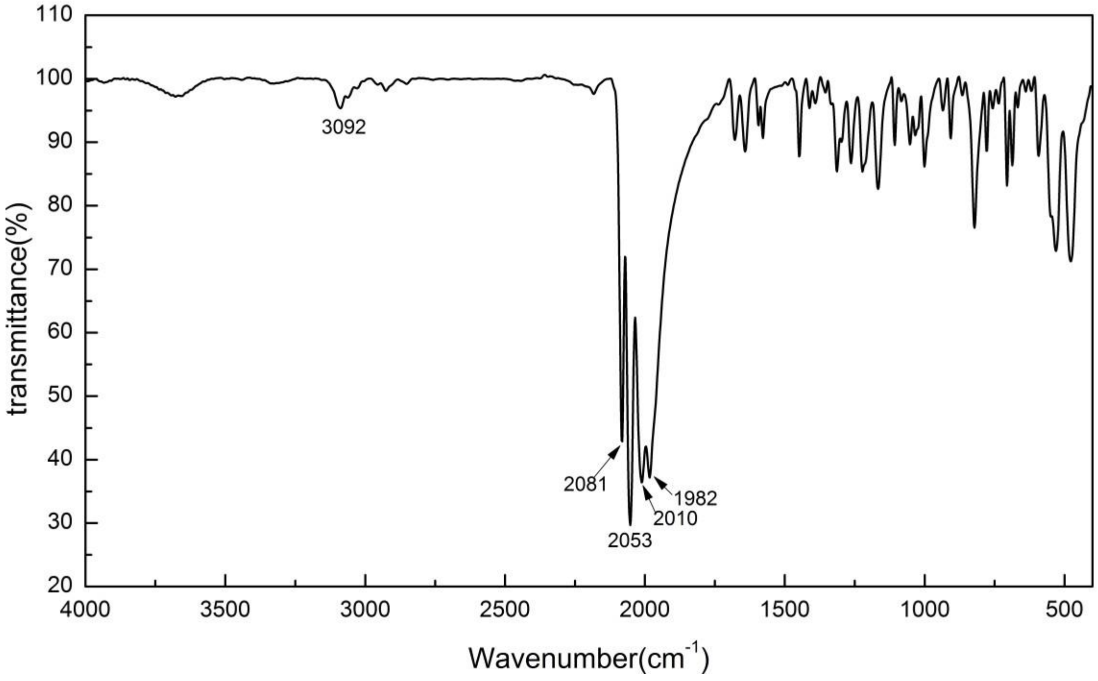

IR spectrum of compound **1b**.

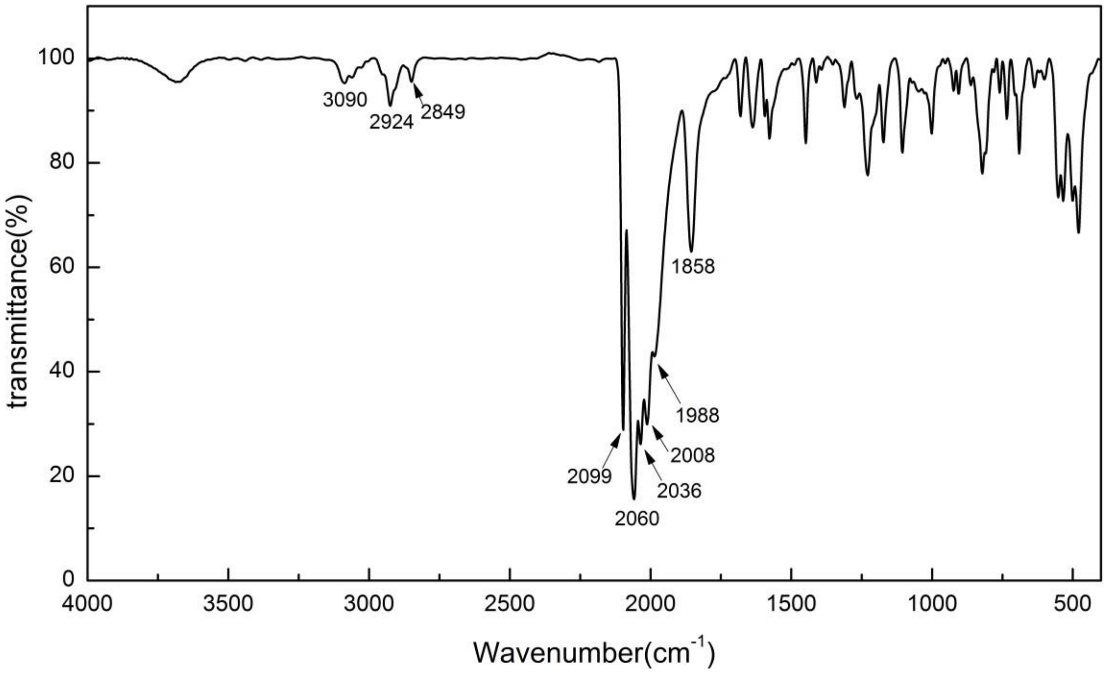

IR spectrum of compound **1c**.

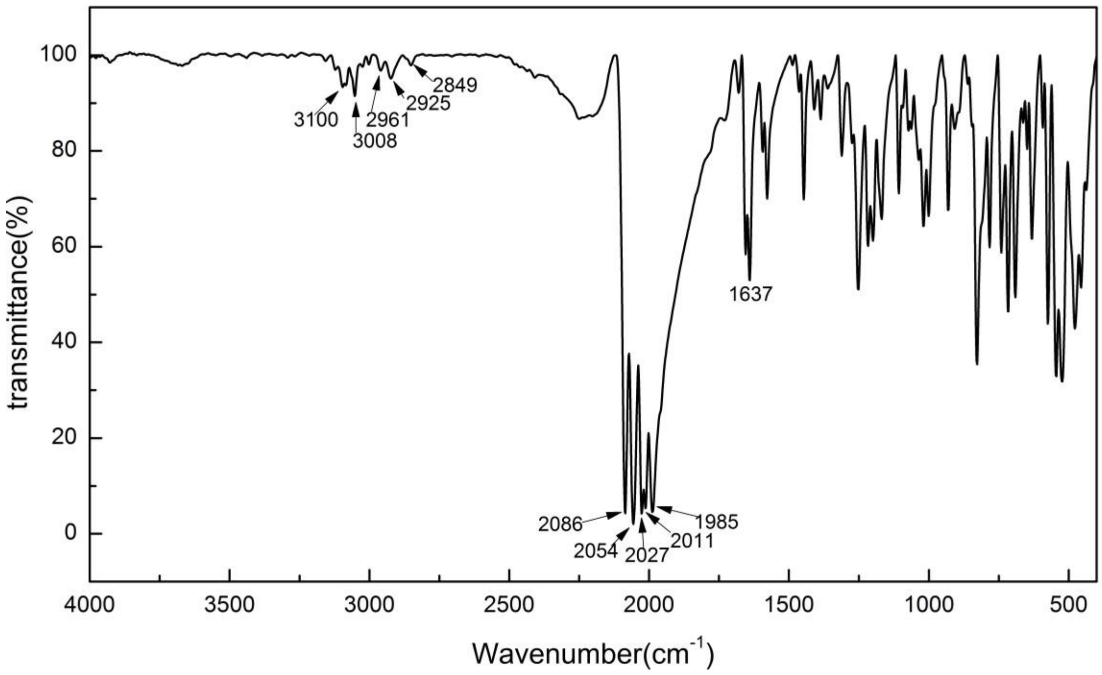

IR spectrum of compound **1d**.

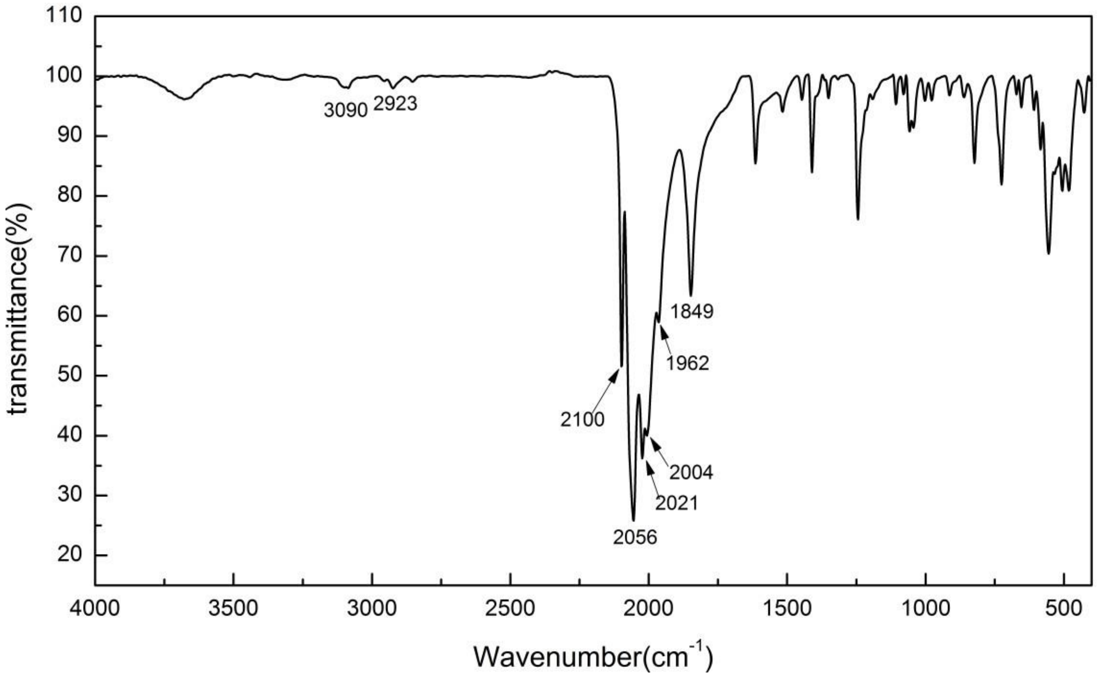

IR spectrum of compound **2a**.

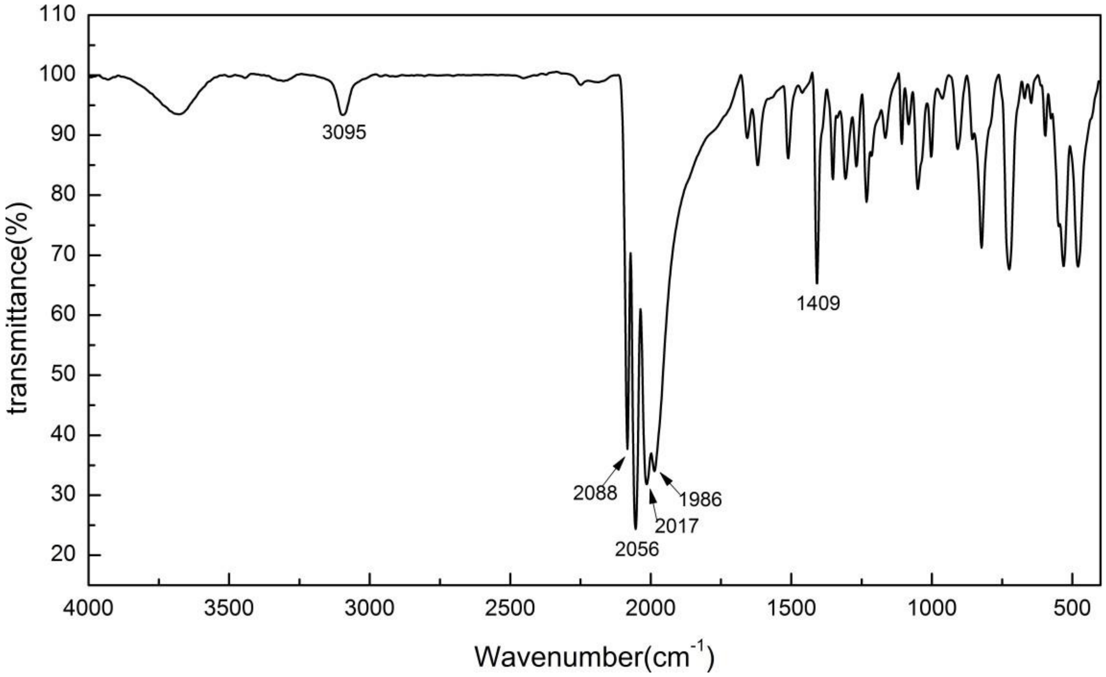

IR spectrum of compound **2b**.

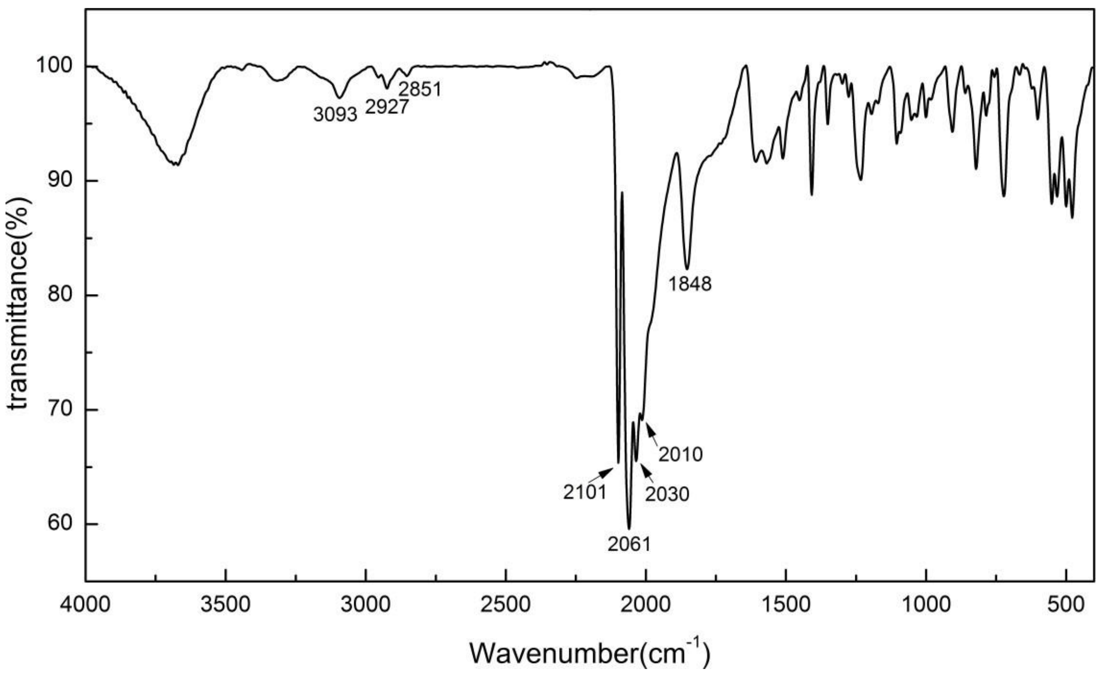

IR spectrum of compound **2c**.

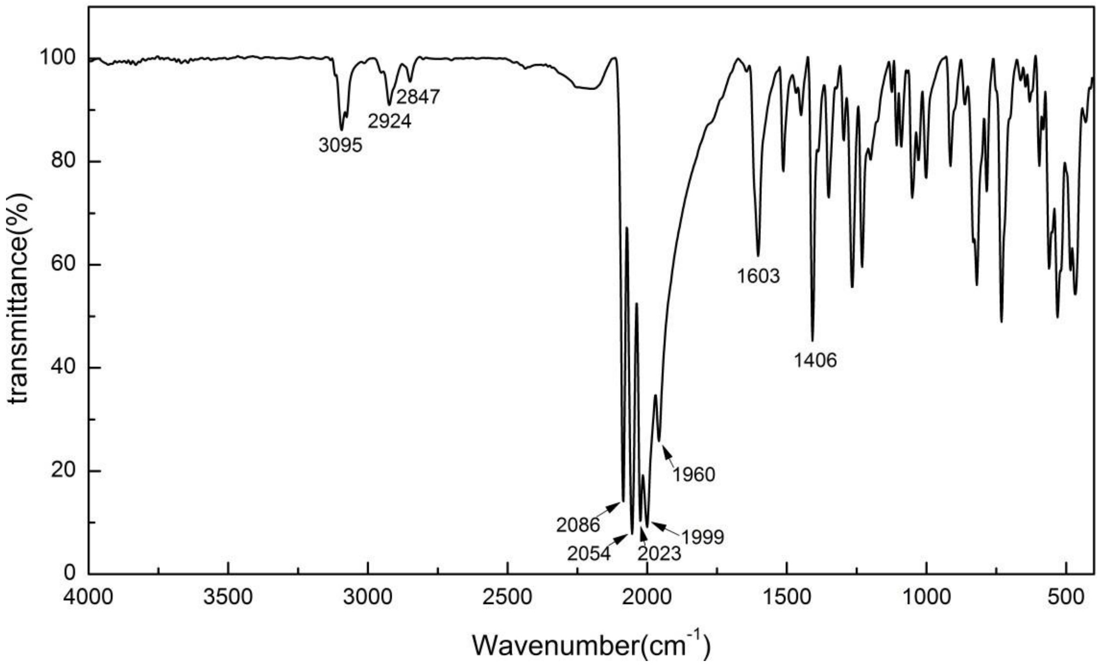

IR spectrum of compound **2d**.

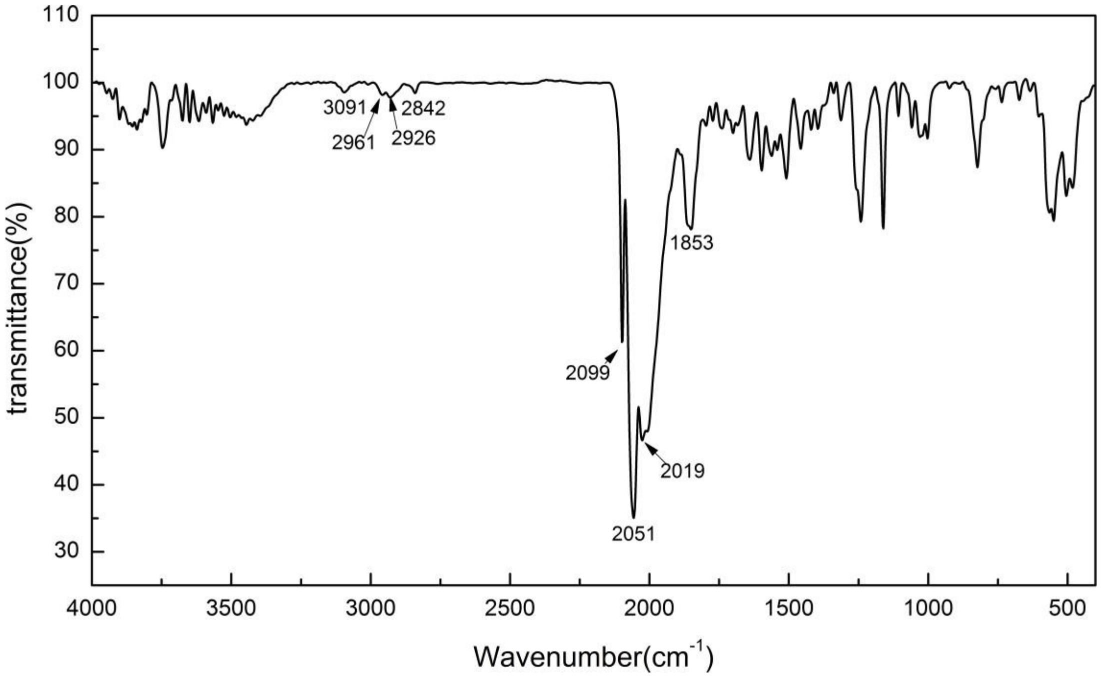

IR spectrum of compound **3a**.

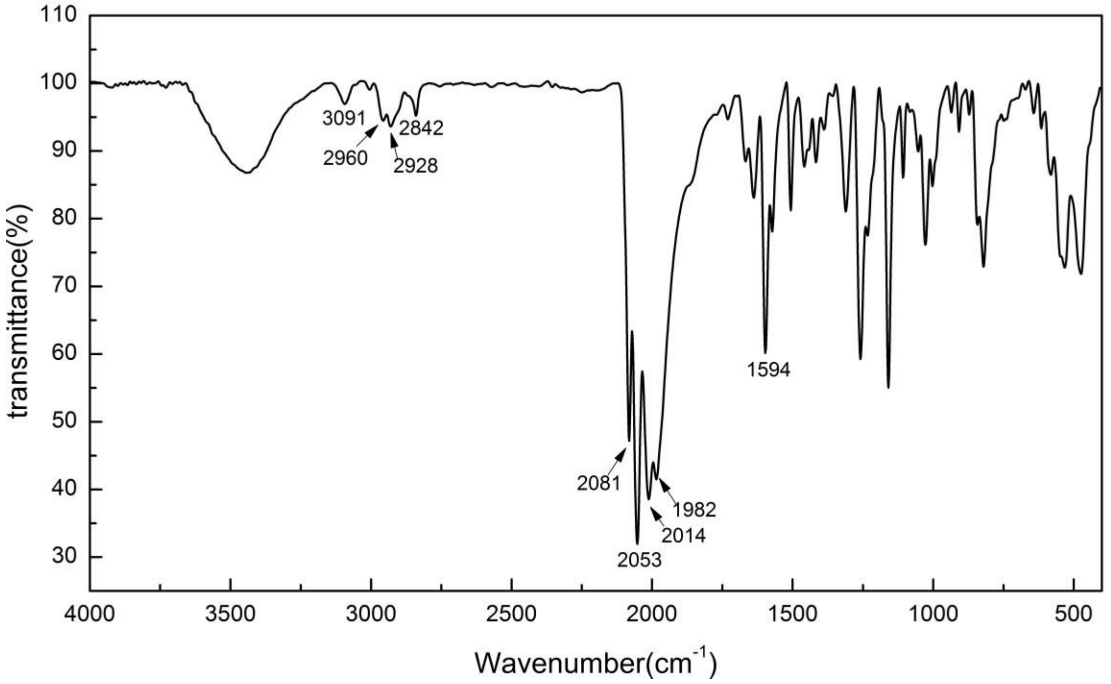

IR spectrum of compound **3b**.

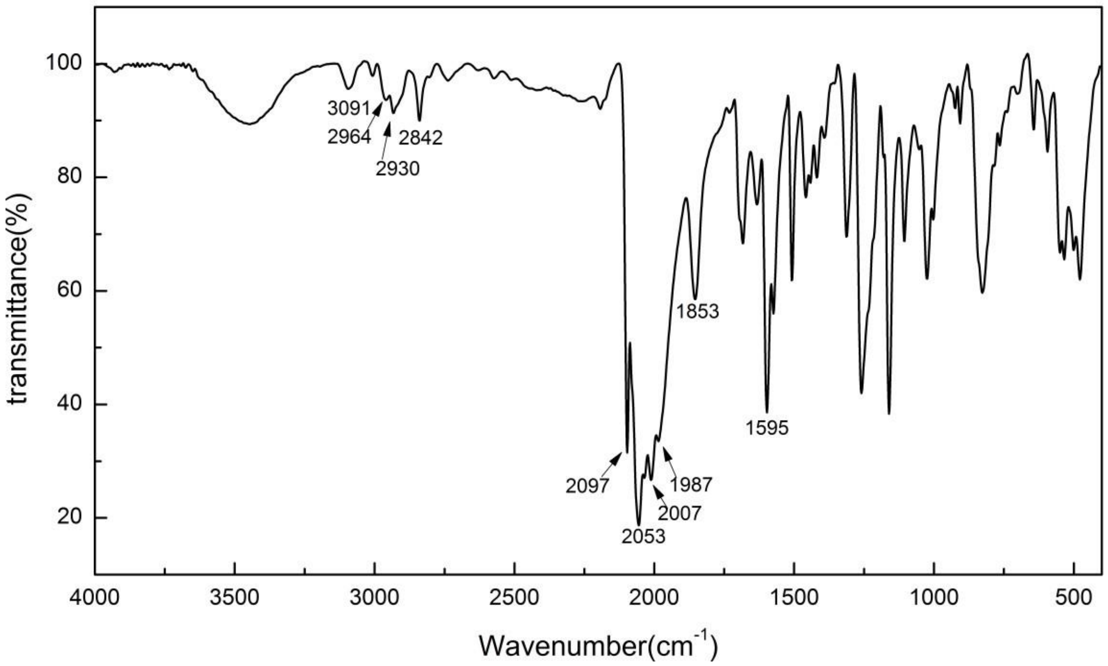

IR spectrum of compound **3c**.

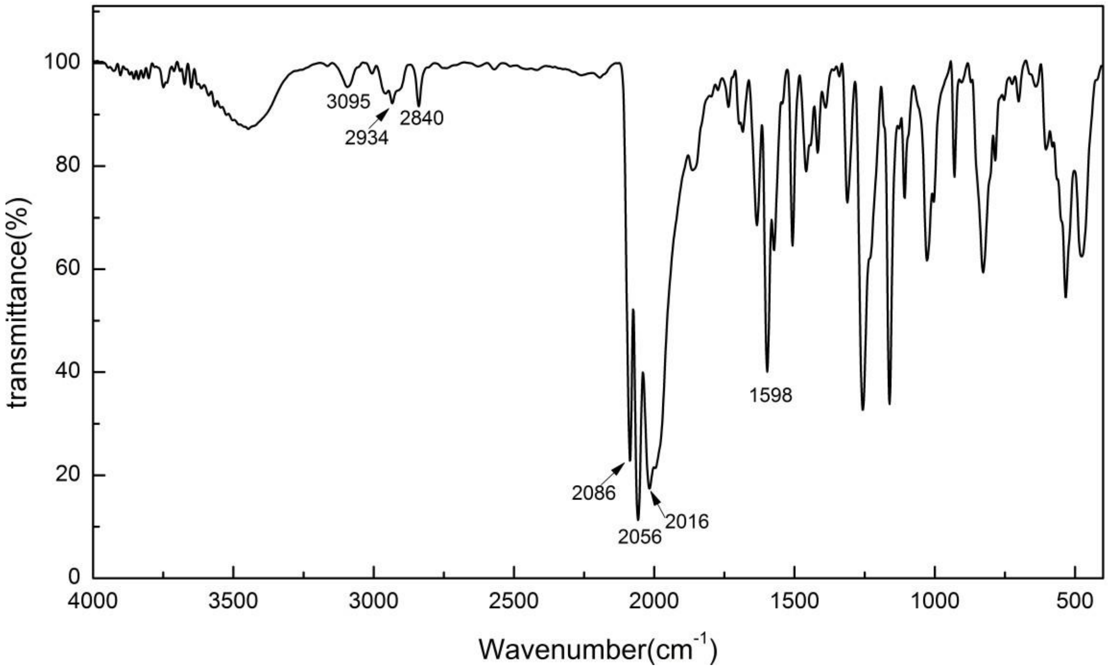

IR spectrum of compound **3d**.

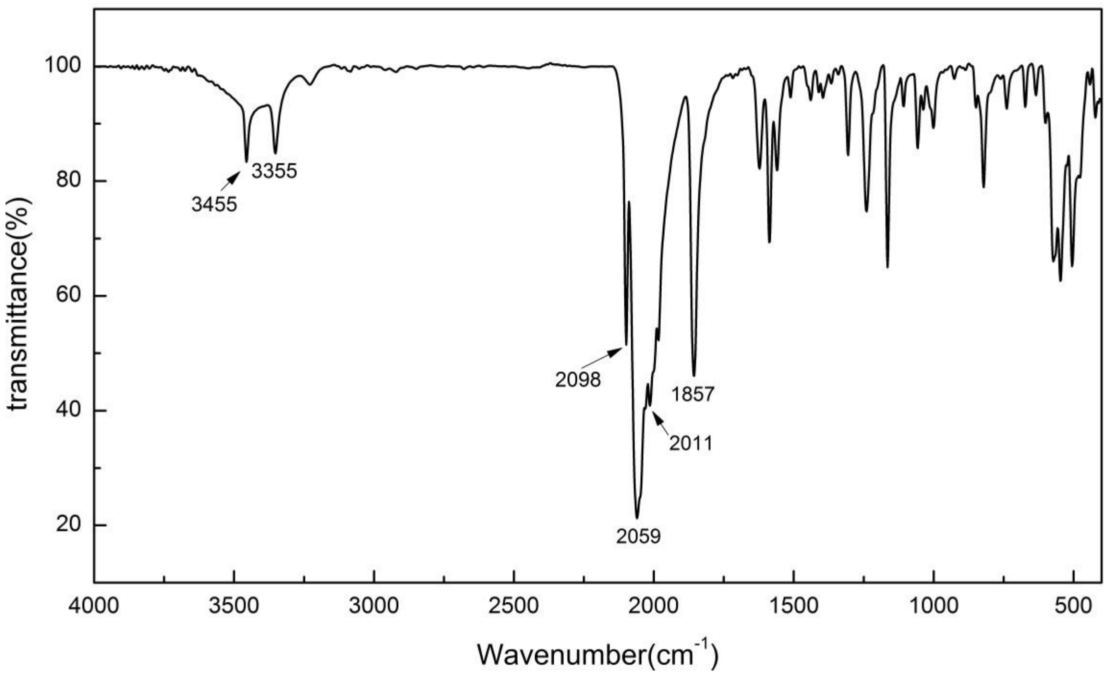

IR spectrum of compound **4a**.

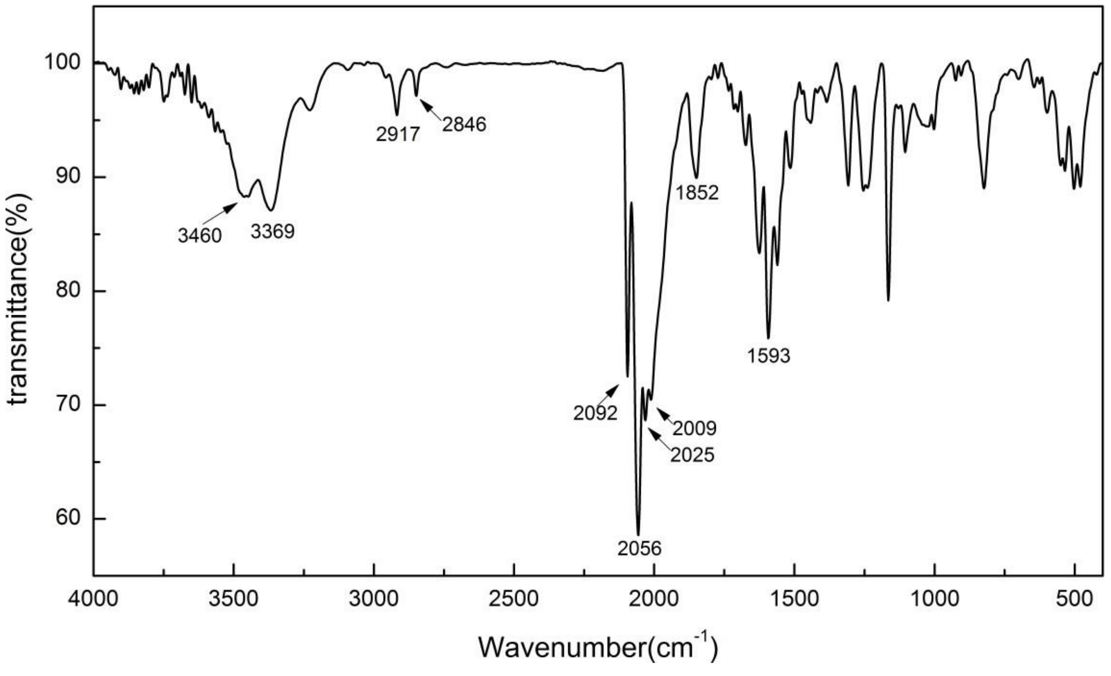

IR spectrum of compound **4b**.

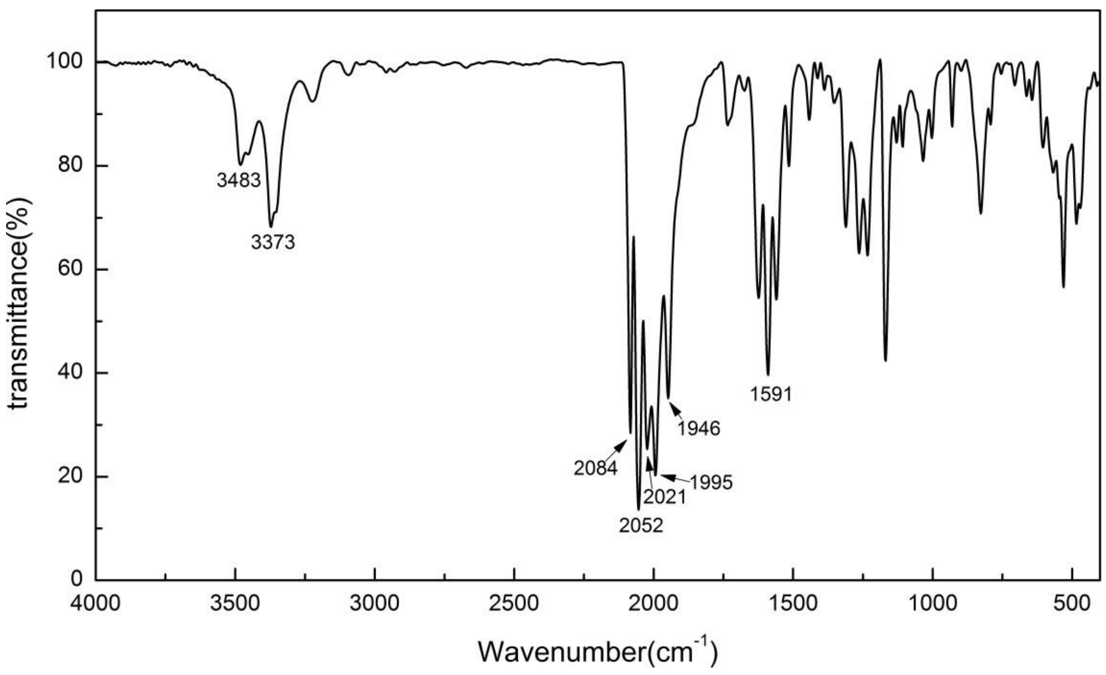

IR spectrum of compound **4c**.

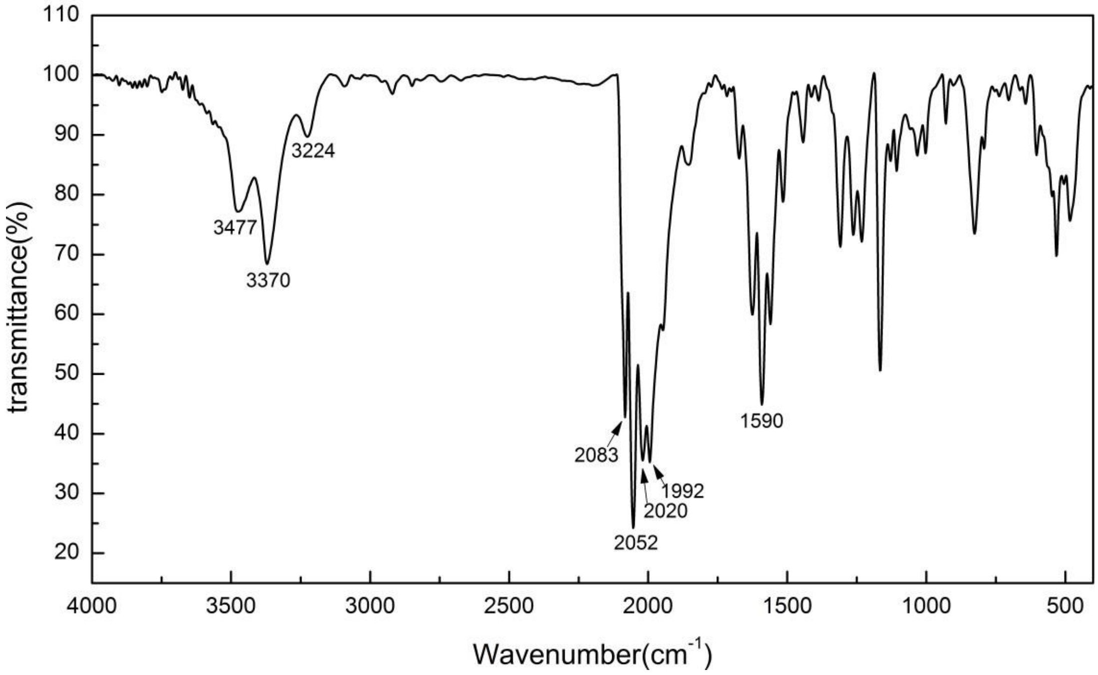

IR spectrum of compound **4d**.

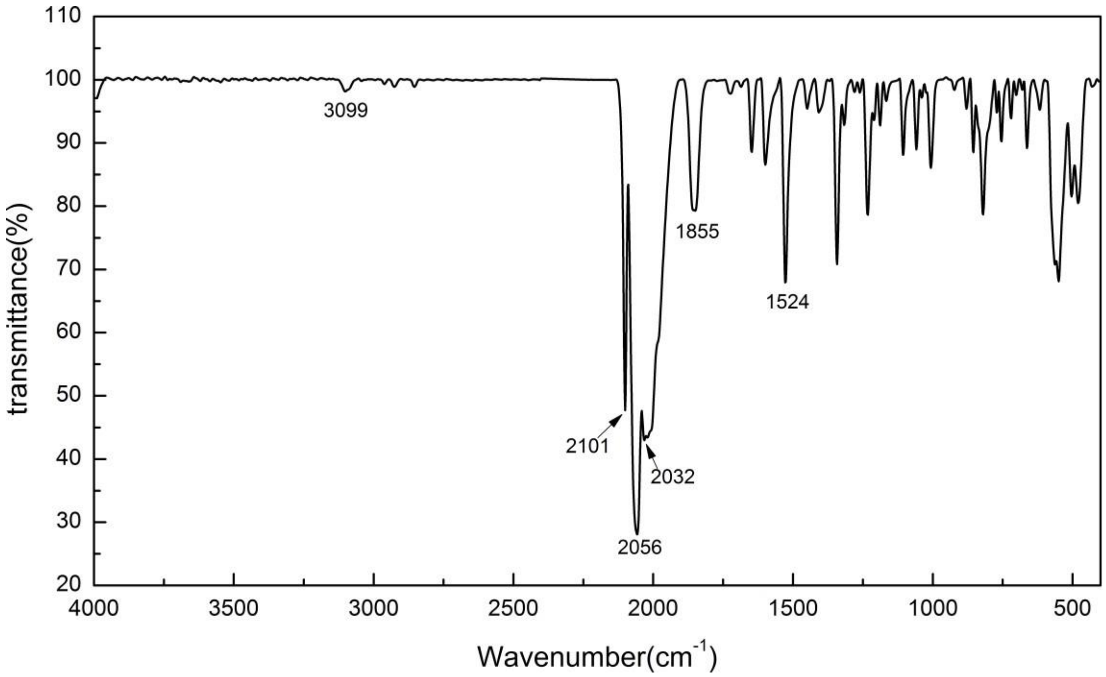

IR spectrum of compound **5a**.

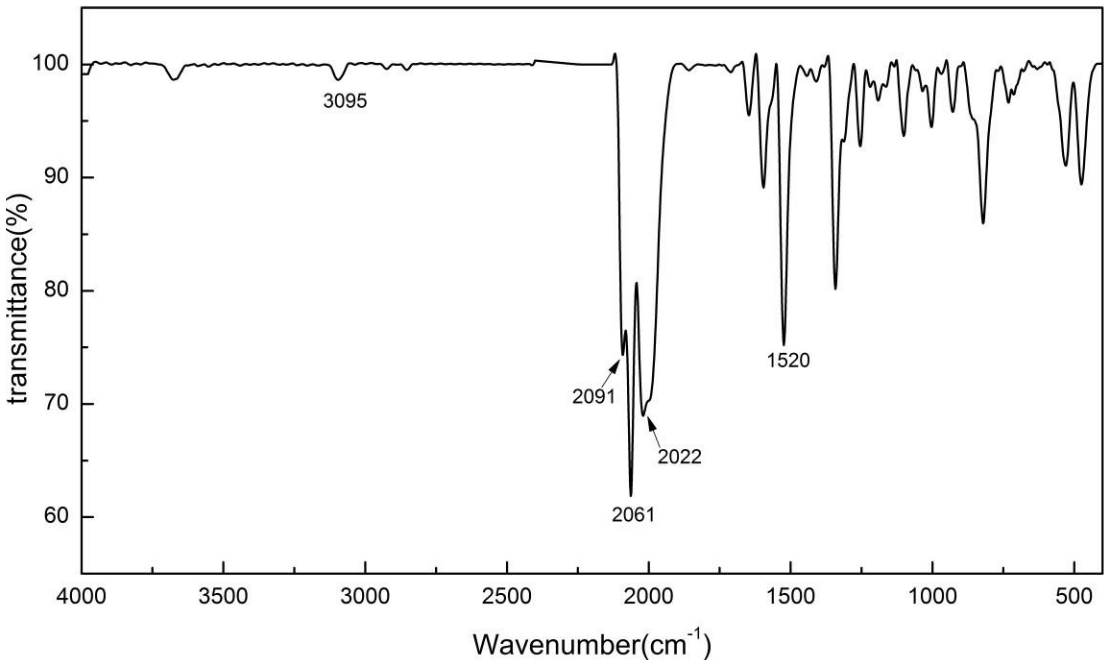

IR spectrum of compound **5b**.

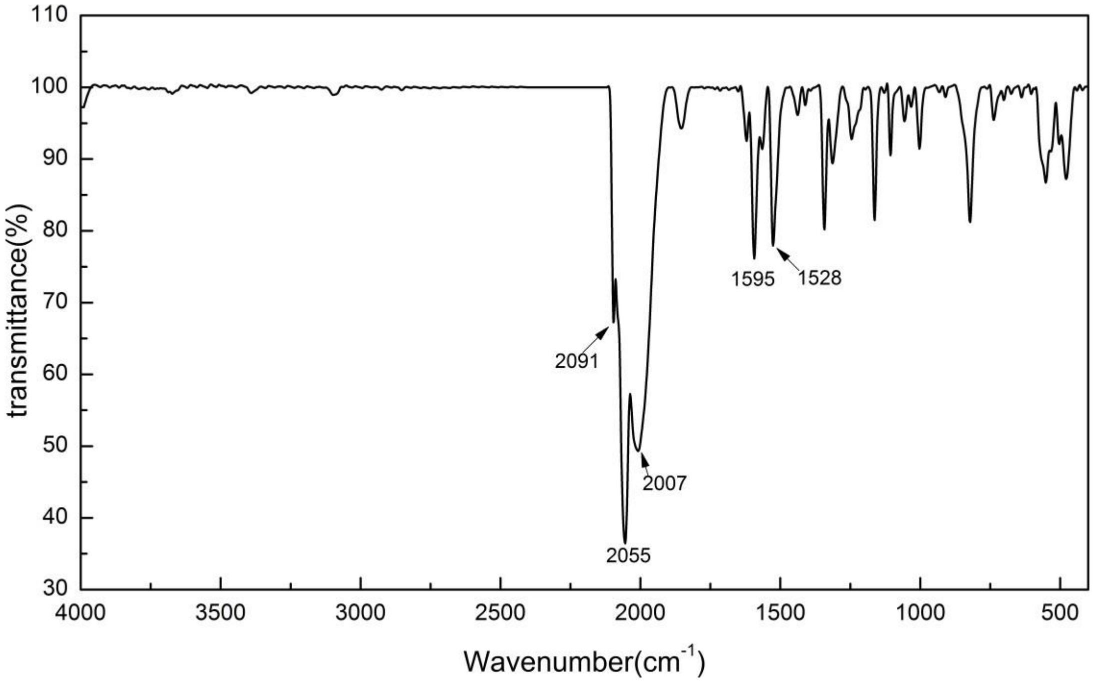

IR spectrum of compound **5c**.

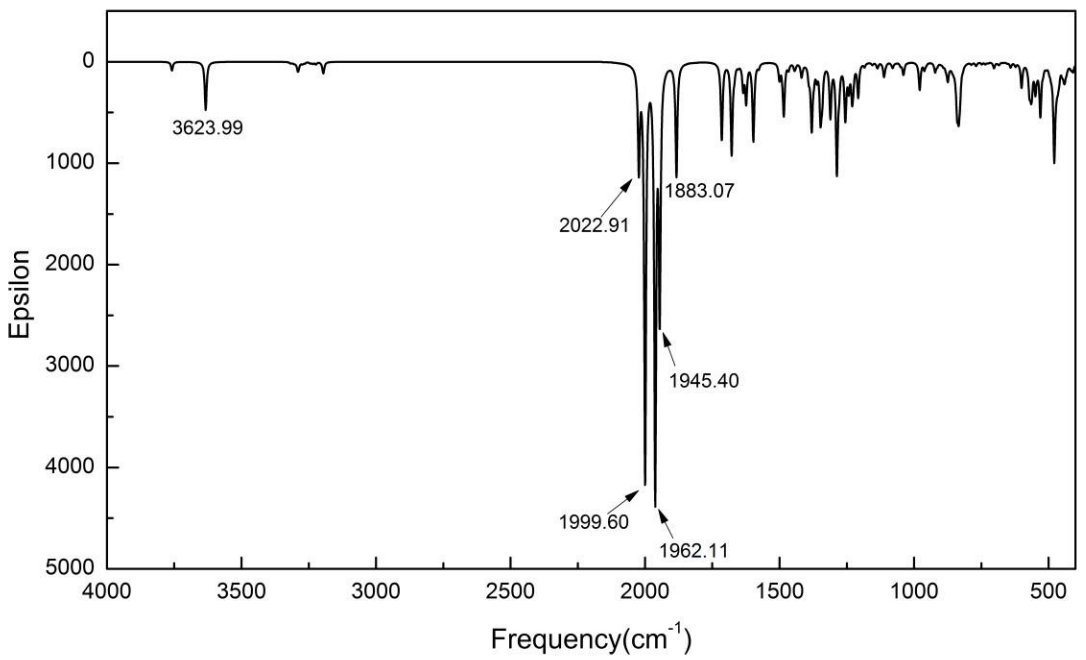

Calculated IR spectrum of compound **5c**.

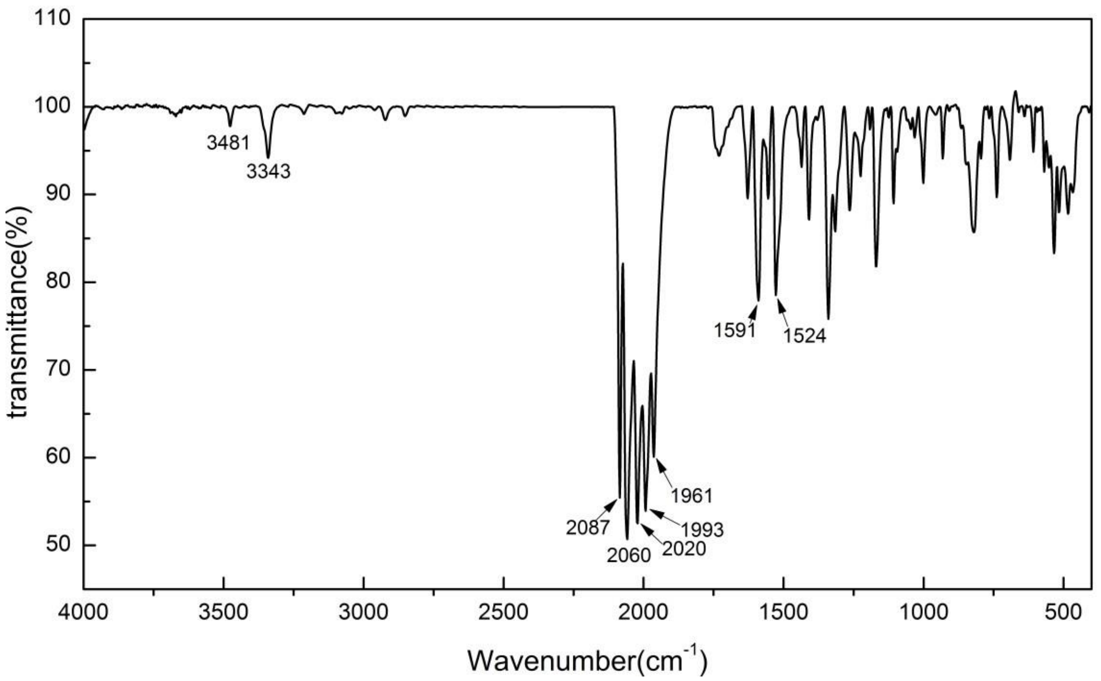

IR spectrum of compound **5d**.

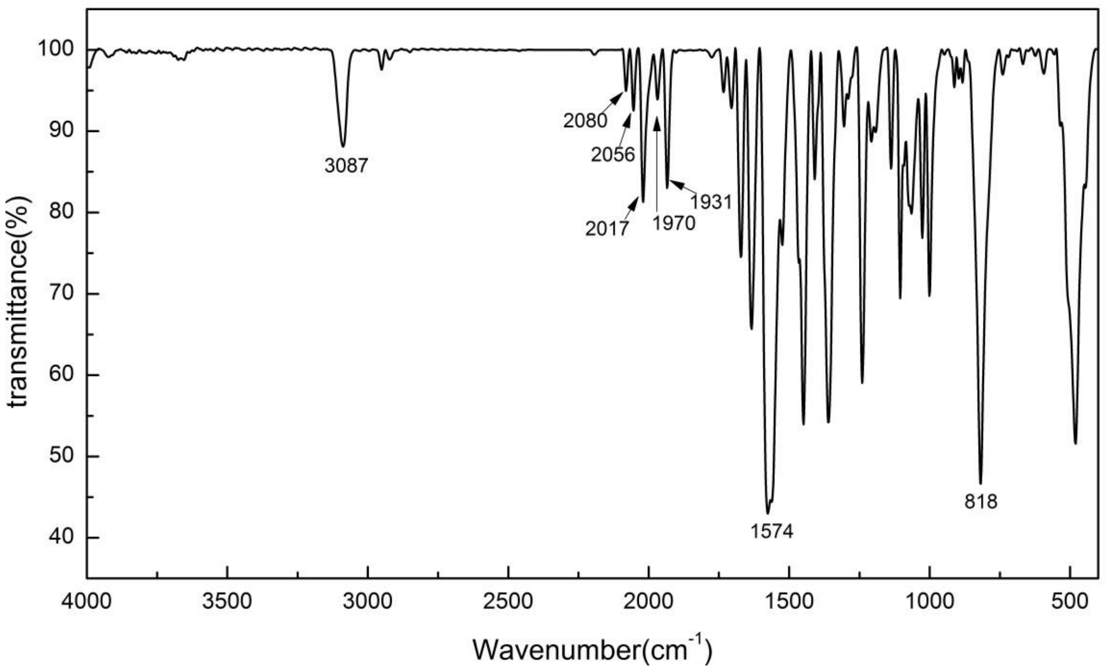

IR spectrum of compound **6a**.
